# Metabolic cooperation supports mutual growth between the protozoan parasite *Trichomonas vaginalis* and dysbiotic vaginal bacteria

**DOI:** 10.1371/journal.ppat.1014620

**Published:** 2026-09-23

**Authors:** Annabel S. Hinderfeld, Raphael Bang, Bailey S. Dickson, Saif Faraj, Augusto Simoes-Barbosa

**Affiliations:** School of Biological Sciences, University of Auckland, Auckland, New Zealand; University of Toronto, CANADA

## Abstract

The protozoan parasite *Trichomonas vaginalis*, the causative agent of trichomoniasis, is frequently associated with dysbiotic vaginal microbial communities characteristic of bacterial vaginosis (BV). While previous studies have shown that vaginal bacteria can modulate parasite pathogenicity, the metabolic basis underlying this ecological association remains unclear. Here, we investigated whether *T. vaginalis* and BV-associated bacteria (BVAB) engage in metabolic cooperation that could support their coexistence. Using in vitro co-culture systems, we examined microbial growth under defined nutrient conditions and integrated metabolomic profiling with targeted transcriptional analysis to characterize metabolic interactions between partners. In serum-free defined medium that does not support proliferation of either microorganism alone, *T. vaginalis* and BVAB exhibited mutualistic growth, indicating that they engage in cooperative metabolic interactions to overcome shared nutritional limitations. Gas chromatography-mass spectrometry revealed a coordinated metabolic shift toward amino acid catabolism during co-culture, with prominent accumulation of ornithine and putrescine linking the arginine dihydrolase (ADH) pathway to polyamine synthesis. Gene expression analyses showed strong upregulation of the parasite carbamate kinase gene during co-culture, implicating enhanced ADH pathway activity in this interaction. Inhibition of polyamine biosynthesis partially impaired the cooperative growth of both microorganisms, further supporting the importance of this metabolic axis. Together, these findings provide experimental evidence that cross-kingdom metabolic cooperation can reciprocally support infection and dysbiosis, highlighting how parasite-microbiome interactions can reshape metabolic networks to overcome environmental constraints and promote their persistence within host-associated microbial ecosystems.

## Author summary

Pathogens rarely occur in isolation. Instead, they interact with other microorganisms that inhabit the same host environment, forming complex communities that can influence disease outcomes. The sexually transmitted protozoan parasite *Trichomonas vaginalis* is strongly associated with bacterial vaginosis (BV), a common imbalance of the vaginal microbiome characterized by the overgrowth of some anaerobic bacteria. However, the biological basis for this association has remained unclear. In this study, we investigated whether the parasite and these bacteria can support each other’s growth through metabolic cooperation. Using laboratory co-culture systems, we found that *T. vaginalis* and BV-associated bacteria can grow together under nutrient conditions that prevent either organism from growing alone. Metabolomic analyses revealed that this cooperative growth is accompanied by a shift toward amino acid metabolism and the accumulation of metabolites involved in alternative energy production and polyamine synthesis. Blocking polyamine production impaired the growth of both organisms in co-culture, indicating that this pathway contributes to their interaction. These findings suggest that metabolic cooperation between pathogen and dysbiotic bacteria may favour infection and help stabilize vaginal dysbiosis. More broadly, our work highlights the importance of studying infections within their microbial context, where interactions between pathogens and bacteria can shape community structure and impact on severity and persistence of disease.

## Introduction

In vaginal trichomoniasis, the extracellular protozoan parasite *Trichomonas vaginalis* is closely associated with a dysbiotic microbial community known as community state type IV (CST-IV) [1]. This community, similar to that found in bacterial vaginosis (BV), is characterized by the depletion of protective *Lactobacillus* species and the overgrowth of anaerobic bacteria, including core BV-associated taxa such as *Gardnerella vaginalis*, *Atopobium vaginae*, and *Prevotella bivia*. BV, the most common dysbiotic condition of the vaginal microbiome, is driven by multiple factors, including alterations in vaginal pH, sexual activity, hormonal changes, and immune responses [2]. Recent studies suggest that *T. vaginalis* may actively contribute to this dysbiotic shift [3,4]. Both trichomoniasis and BV are prevalent among reproductive-age women and are associated with significant inflammation of the cervicovaginal mucosa, leading to discomfort and excessive vaginal discharge. In addition, they are both linked to adverse obstetric outcomes, increased risks of HIV transmission, and development of cervical cancer [2,4].

Vaginal community state types I, II, and V are predominantly dominated by individual species of *Lactobacillus* [5]. These species (*L. crispatus*, *L. gasseri*, and *L. jensenii*, respectively) produce high levels of lactic acid, which lowers vaginal pH and inhibits the colonization of species of BV-associated bacteria (BVAB) [2]. The maintenance of an acidic vaginal environment in women is closely linked to cyclical hormonal fluctuations, particularly the rise of estrogen, which stimulates glycogen production in vaginal epithelial cells being subsequently metabolized into lactic acid by lactobacilli. Additionally, vaginal epithelial cells contribute to luminal acidity through active estrogen-dependent proton secretion. This microbe-host relationship is unique to the genital tract of women, as no other mammal maintains such a low vaginal pH (~3.8-4.5) in association with a high abundance of lactobacilli, reflecting a relatively recent evolutionary adaptation in humans [6].

When this mutualistic relationship with lactobacilli is disrupted, BV-associated bacteria might flourish. This transition is accompanied by a rise in vaginal pH and significant changes in metabolites as measured from cervicovaginal fluid [7] and through 3D epithelial cell models of microbial colonization [8]. These metabolic shifts are typically characterized by the accumulation of amino acid catabolites, polyamines, and other molecules with proinflammatory potential [7,8]. The stability of BV dysbiotic community relies on metabolic cooperation among associated bacteria, particularly within biofilms [8], which are a central to BV resilience and pathogenicity [2].

Beyond bacteria, the awareness of protozoa as integral members of host-associated microbial ecosystems is steady increasing by the microbiome scientific community [9]. Gut protists interact with resident microbial communities and with specificity, influencing both microbial composition and host physiology. Experimental studies have demonstrated that bacterial communities can modulate the pathogenicity of several protozoa while protozoa themselves – commensals or parasites - can reshape microbial communities through nutrient competition, metabolite exchange, and secretion of bioactive molecules [9,10].

Historical precedent from studies with the protozoan parasite *Entamoeba histolytica* shows that the impact of bacteria in parasite biology and pathogenicity has been recognized for more than a century. Early observations suggested that gut bacteria influence the development of amebic dysentery, and subsequent work demonstrated that trophozoites depend on bacterial interactions for optimal growth and virulence [11]. Experimental studies using germ-free and gnotobiotic animal models further showed that the presence of specific bacterial species is required for the development of intestinal lesions and invasive disease, and that reassociation with bacteria restores virulence in otherwise attenuated amoebae [12–15]. Subsequent work demonstrated that *E. histolytica* trophozoites can directly interact with bacteria through attachment and ingestion processes that stimulate parasite virulence traits and metabolic activity [11,15]. The *Entamoeba*-bacteria field resurged in the late 1990s, when molecular studies began to demonstrate how specific bacterial partners modulate parasite virulence pathways and host inflammatory responses [16–18], reinforcing the concept that cross-kingdom interactions are central to amoebic pathogenesis. A similar historical perspective exists for trichomoniasis, with early work reviewed by Jírovec and Petrů describing how bacteria can influence the cultivation, growth, and biological behaviour of *T. vaginalis* [19].

The growing recognition of protists as members of animal-associated microbiomes challenges the traditional dichotomy between commensalism and parasitism and instead suggests a continuum between these states [9]. Experimental studies in mice with commensal gut parabasalids - closely related to *T. vaginalis* - provide compelling examples. In one study, gut trichomonads activated inflammasome-dependent mucosal immune responses that enhanced resistance to enteric bacterial pathogens while also increasing susceptibility to inflammatory disease [20]. In a second study, colonization by gut trichomonads profoundly altered immune homeostasis expanding intestinal Th1 responses and exacerbating colitis [21]. Together, these findings illustrate how trichomonad protists can modulate host physiology and disease susceptibility, highlighting their potential role as ecological drivers within host-associated microbial ecosystems [9].

If protists are not merely inhabitants but ecological drivers that can reshape host-associated microbial ecosystems, the strong epidemiological association between *T. vaginalis* infection and BV-associated microbial communities raises the possibility that similar cross-kingdom interactions may operate in the vaginal niche. Moreover, studies by our group and others have repeatedly described antagonistic and synergistic interactions between this protozoan and non-dysbiotic and dysbiotic vaginal bacteria [22–24] including pathways and molecules that mediate these interactions [3,25–29]. These studies demonstrate that *T. vaginalis* and different members of the vaginal microbiota engage in complex and ecologically meaningful interactions, involving adhesion-modulating factors, bacteriolytic enzymes, and extracellular vesicle-mediated signaling; broader comparative work further suggests that microbiota-targeting mechanisms are conserved across *Trichomonas* species [30]. Despite these mechanistic insights, it remains unclear whether *T. vaginalis* and BVAB cooperate metabolically to support their coexistence and, therefore, to contribute to the ecological stability of dysbiotic vaginal communities. Since vaginal dysbiosis and trichomoniasis are both associated with inflammation, epithelial disruption, treatment failure, and recurrence, defining the metabolic basis of parasite-bacteria coexistence may reveal how microbial ecology contributes to infection persistence and disease outcome.

Here, we investigated whether *T. vaginalis* and BVAB engage in metabolic cooperation that might help explain their persistence within dysbiotic vaginal communities. To address this question, we developed in vitro co-culture systems that allowed us to examine mutual growth under defined nutrient conditions and to characterize metabolic dependencies that may emerge between the protozoan and bacterial partners. Using these experimental systems, we combined microbial co-growth analyses with metabolomic profiling and targeted transcriptional analysis to explore how these interactions shape the metabolic state of the co-cultures.

## Results

### *Trichomonas vaginalis* and BVAB exhibit mutualistic growth

*T. vaginalis* and the three selected BVAB - *G. vaginalis*, *A. vaginae*, and *P. bivia* - are routinely cultivated in nutrient-rich media that provide complex organic nutrients, reducing conditions, and serum-derived growth factors. These media typically include complex organic nutrients (e.g., peptones and yeast extract), reducing agents (e.g., cysteine and ascorbic acid), and serum as a rich source of iron and amino acids. Additionally, *T. vaginalis* and these three species of BVAB share certain metabolic traits, such as they both thrive under anaerobic or microaerophilic conditions and metabolize amino acids and polyamines [4,7].

Although their specific nutritional requirements may differ, these shared features suggest that a single medium could be used to test whether metabolic interactions between *T. vaginalis* and BVAB support their mutual growth. In this study, we hypothesized that *T. vaginalis* and BVAB may engage in metabolic cooperation that provides mutual growth benefits in co-culture. To test this hypothesis, we conducted a series of in vitro co-culture experiments to identify conditions under which metabolic interactions between these microorganisms could be further explored.

First, we compared the growth of *T. vaginalis* in its optimal medium (i.e., TYM with serum) between axenic and mixed cultures in a time-course experiment, where the latter received a joint inoculum of all three BVAB (Fig 1). As controls, the three BVAB were also inoculated together in the same medium without the protozoan and vice-versa. We enumerated the protozoan from the broth culture using flow cytometry and assessed bacterial growth qualitatively by spot plating on agar. It is important to note that, while *T. vaginalis* cannot grow on agar spot plates, the BVAB cannot be differentiated via their standard agar media. In axenic cultures, *T. vaginalis* exhibited a typical growth behavior, progressing through lag, exponential, and stationary phases. BVAB also grew effectively in TYM, as expected from their similar media requirements. However, in co-culture, *T. vaginalis* appeared to control the growth of the bacteria to some extent, as suggested from the agar spot plates. Notably, *T. vaginalis* reached the logarithmic phase earlier when co-cultured with the BV bacteria (Fig 1; p < 0.01 at 24 and 28 h).

**Fig 1:**
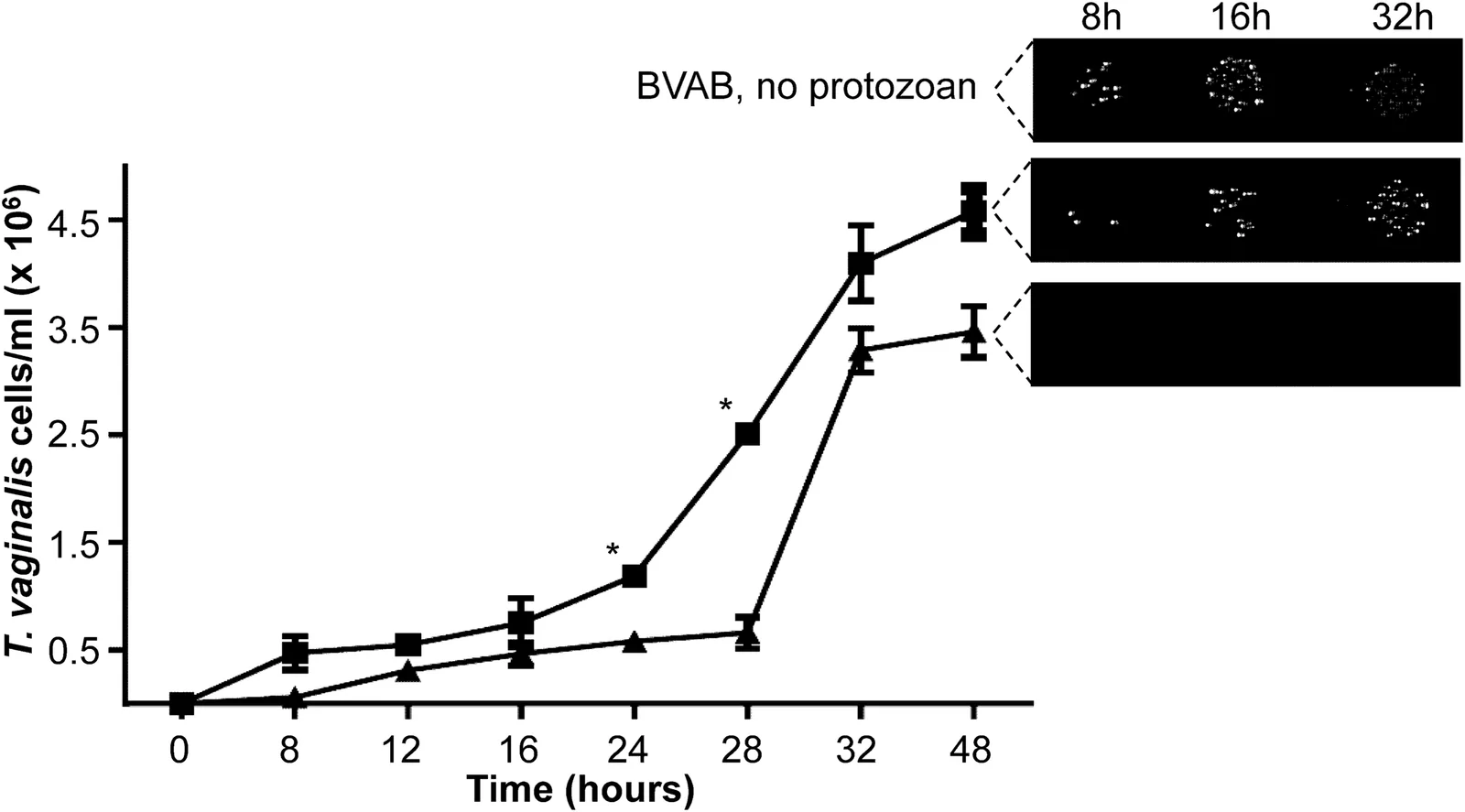
Growth of *T. vaginalis* and BV-associated bacteria in TYM medium. Curve plots represent the anaerobic growth of *T. vaginalis* axenically (triangles) or a with a joint inoculum of the three BV-associated bacteria (BVAB) *P. bivia, A. vaginae* and *G. vaginalis* (square), with an initial inoculum of 2 x 10^4^ protozoal cells per ml. Similarly, spot plating represents the anaerobic growth of the BVAB inoculated together in the same medium with or without the protozoan. At 24 h and 28 h timepoints, *T. vaginalis* cell numbers were higher in mixed than axenic cultures (*p < 0.01; one-way ANOVA) indicating that logarithmic phase was reached earlier when co-cultured with BVAB. Data are shown as mean + /- standard deviation (SD) from at least three independent biological replicates.

Next, we examined the co-culturing of *T. vaginalis* and BVAB in defined DMEM without serum, a condition that would not support growth of either protozoan or bacteria. Without bacteria, *T. vaginalis* viability under anaerobiosis in this medium remained high for up to 48h but, as expected, no growth was observed (Fig 2). However, when co-incubated with the BVAB, *T. vaginalis* populations multiplied over the course of 48 h (Fig 2). To determine if this response was dependent on specific bacteria, we conducted additional co-culture experiments with *T. vaginalis* alongside individual species of BVAB or combinations of two. For comparison, we also co-cultured *T. vaginalis* with *L. gasseri*, representing eubiosis (CST II) of the vaginal microbiome. *L. gasseri* did not support *T. vaginalis* growth; in fact protozoa numbers slightly declined. *G. vaginalis* seemed to provide the best support for *T. vaginalis* growth across the individual species of BVAB, while some additive effects were observed with two bacterial species (Fig 3).

**Fig 2:**
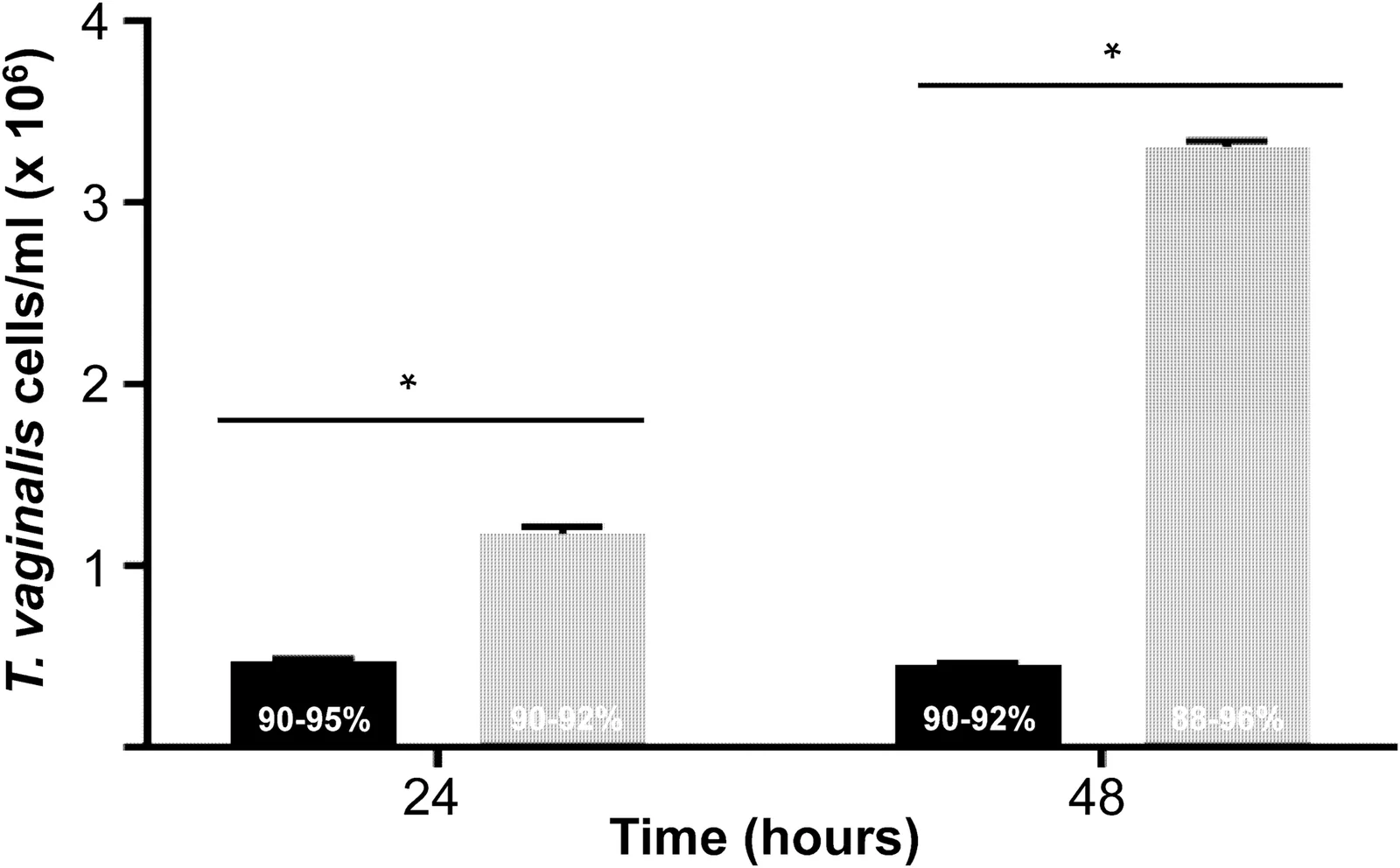
Number and viability of *T. vaginalis* cells following anaerobic incubation in serum-free defined medium DMEM with or without the BV-associated bacteria. From the initial inoculum of 5 x 10^5^ cells/ml, the number of *T. vaginalis* cells remained constant with high viability (%) in the absence of the BV-associated bacteria (BVAB) *P. bivia, A. vaginae* and *G. vaginalis* (black bars) until the end of the incubation period. In contrast, the protozoal cell numbers approximately doubled and quadrupled at 24 and 48h, respectively, when co-cultured with BVAB (gray bars; *p < 0.01; one-way ANOVA). *T. vaginalis* viability was assessed using the TC20 automated cell counter, and parasite enumeration was performed by flow cytometry, as described in Materials and Methods. Data are shown as mean + /- SD from at least three independent biological replicates.

**Fig 3:**
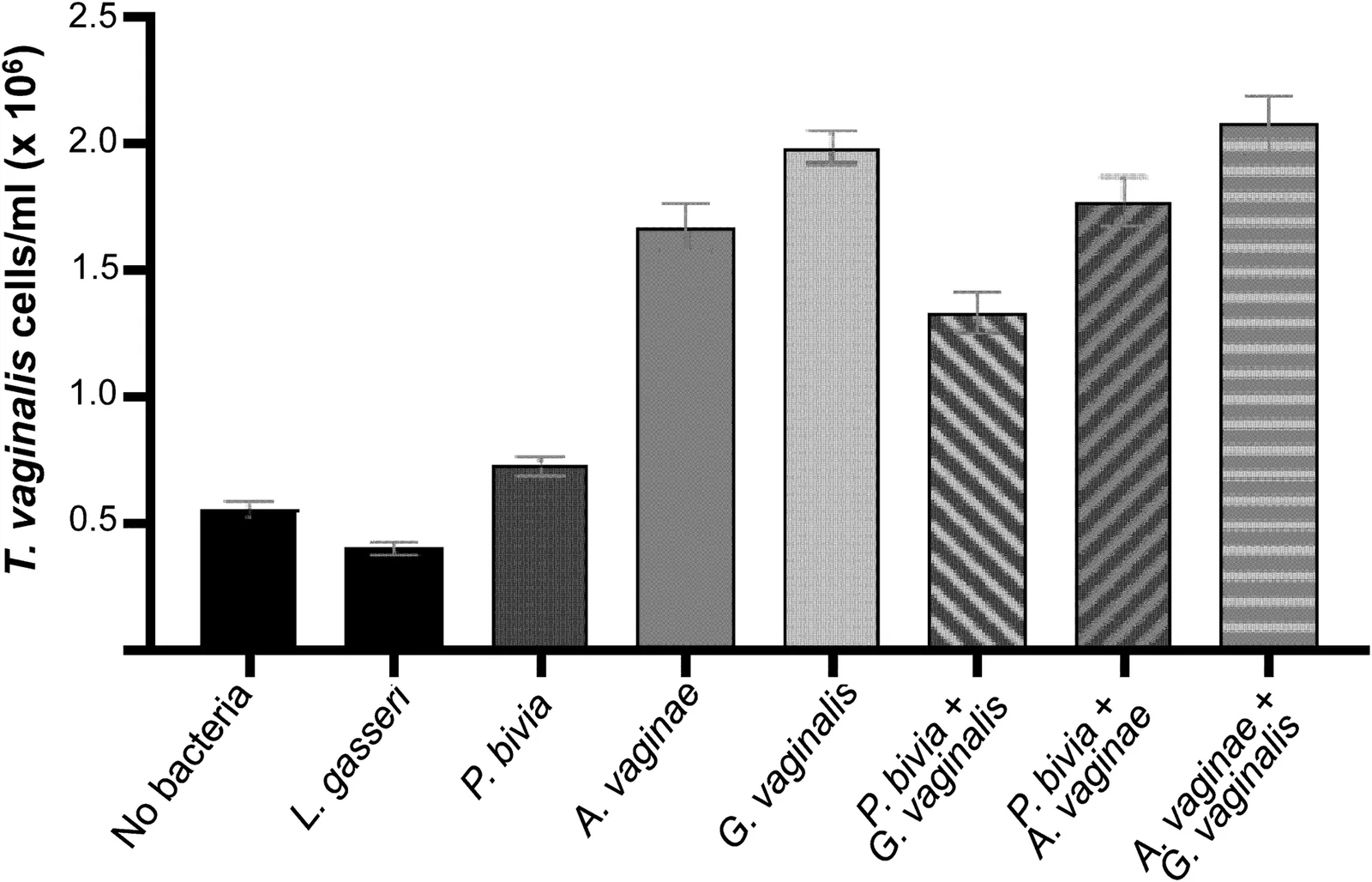
Number of *T. vaginalis* cells following anaerobic incubation for 48h in serum-free defined medium DMEM with or without non-dysbiotic and dysbiotic vaginal bacteria. From the initial inoculum of 5 x 10^5^ cells/ml, non-dysbiotic *L. gasseri* caused a modest but significant reduction in *T. vaginalis* cell numbers compared with the no-bacteria control. Conversely, all single species or combinations of two species of BV-associated bacteria significantly increased *T. vaginalis* cell numbers, reaching up to ~4-fold higher numbers than the no-bacteria control. Bars represent mean + /- SD from at least three independent biological replicates. Statistical significance was assessed by one-way ANOVA followed by Dunnett’s multiple-comparisons test against the no-bacteria control; p < 0.01).

Finally, we compared growth curves of *T. vaginalis* in DMEM without serum between axenic and mixed cultures, where the latter received a joint inoculum of all three BVAB (Fig 4). As control, the three BVAB were inoculated together in the same medium but without *T. vaginalis*. As before, despite being highly viable, *T. vaginalis* did not grow axenically in this condition while BVAB sharply declined in the absence of the protozoan. Surprisingly, in the presence of BVAB, *T. vaginalis* sustained growth over time. Despite apparently delayed as compared to its optimal growth in TYM medium, *T. vaginalis* population reached a substantial cell number between 5–6 X 10^5^ cells per mL (~4–5 times lower than its optimal growth). Also surprisingly, BVAB sustained substantial growth when co-cultured with *T. vaginalis* in this restrictive media. In conclusion, *T. vaginalis* and BVAB demonstrate a unique behaviour of mutual growth in a nutritional condition that is growth-restrictive to the protozoan and bacteria in isolation. This observation indicates that protozoan and BVAB must engage metabolically to support their cooperative growth when in mixed cultures.

**Fig 4:**
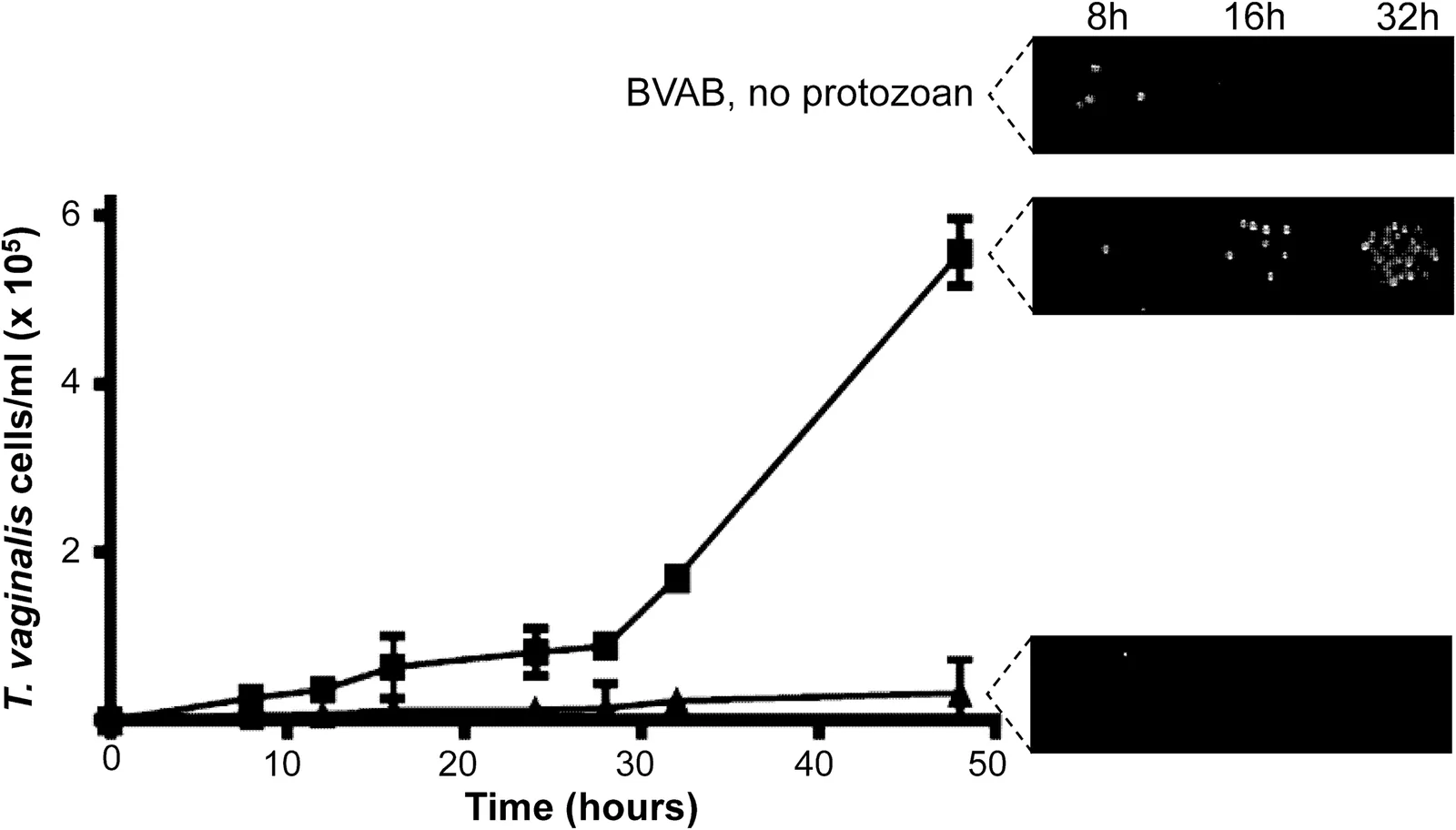
The mutualistic growth behaviour of *T. vaginalis* and BV-associated bacteria in serum-free defined medium DMEM. Curve plots represent the anaerobic growth of *T. vaginalis* axenically (triangles) or a with a joint inoculum of the BV-associated bacteria (BVAB) *P. bivia, A. vaginae* and *G. vaginalis* (square), with an initial inoculum of 2 x 10^4^ protozoal cells per ml. Protozoal cell viability remained high (> 92%) for both conditions and across time points. Spot plating represents the anaerobic growth of the BVAB inoculated together in the same medium with or without the protozoan. Microbial co-growth is mutual, as both the protozoan and bacteria grow under conditions that do not support the proliferation of either organism alone. Data are shown as mean + /- SD from at least three independent biological replicates.

### Mutualistic growth of protozoan and BVAB is accompanied by a shunt toward amino acid metabolism

Shifts in metabolite production are a hallmark of BV [7]. *T. vaginalis* is known for engaging in metabolic cooperation with its endosymbiont, *Mycoplasma hominis* [31,32]. *M. hominis*, but not “*Ca. M. girerdii*” was detected in the G3 cultures used for these experiments (S1 Fig). With these precedents, we wanted to further investigate the metabolic interactions between *T. vaginalis* and BVAB by examining changes in metabolite profiles and gene expression during co-culture experiments. These analyses could help us identify specific metabolic pathways and regulatory mechanisms that may facilitate their mutual growth in the defined medium (Fig 4). To achieve this, we applied gas chromatography-mass spectrometry (GC-MS) to compare the relative abundances of individual metabolites between *T. vaginalis*-BVAB mixed cultures and *T. vaginalis*-axenic cultures in serum-free DMEM. The mixed cultures provided the metabolic conditions for mutualistic growth between *T. vaginalis* and BVAB (Fig 4).

Metabolites, obtained from these cultures at three specified timepoints, were run through GC-MS and data were collected for analysis. After processing the metabolomics data from the time-course experiment (S1 Data), principal component analysis conducted on both MCF and TMS derivatization datasets revealed consistent trends across timepoints (S2 Fig). At the earliest timepoint (8h), primary variation in metabolite profiles (PC1) was not associated with the experimental conditions, indicating minimal metabolic differences. The association of metabolite profiles with experimental conditions became apparent in later timepoints, 16h and 32h for MCF and TMS respectively, suggesting that significant metabolic changes between conditions occur during these stages of growth at least for the timepoints that were subjected to this analysis (S2 Fig).

Next, we analysed the normalized peak responses of over 200 individual metabolites across culture conditions (S2 Data). Metabolites from the MCF and TMS datasets showing the greatest fold-difference between mixed and axenic cultures were ranked and selected as key metabolites of interest (S1 and S2 Tables, respectively). Based on this list and where else possible, the selected ranked metabolites were mapped onto four well-described metabolic pathways: glycolysis, amino acid metabolism, arginine dihydrolase pathway, and polyamine synthesis. Distinct changes in metabolite levels, observed over the three incubation timepoints, were color-coded to compose an integrative metabolic map (Fig 5).

**Fig 5:**
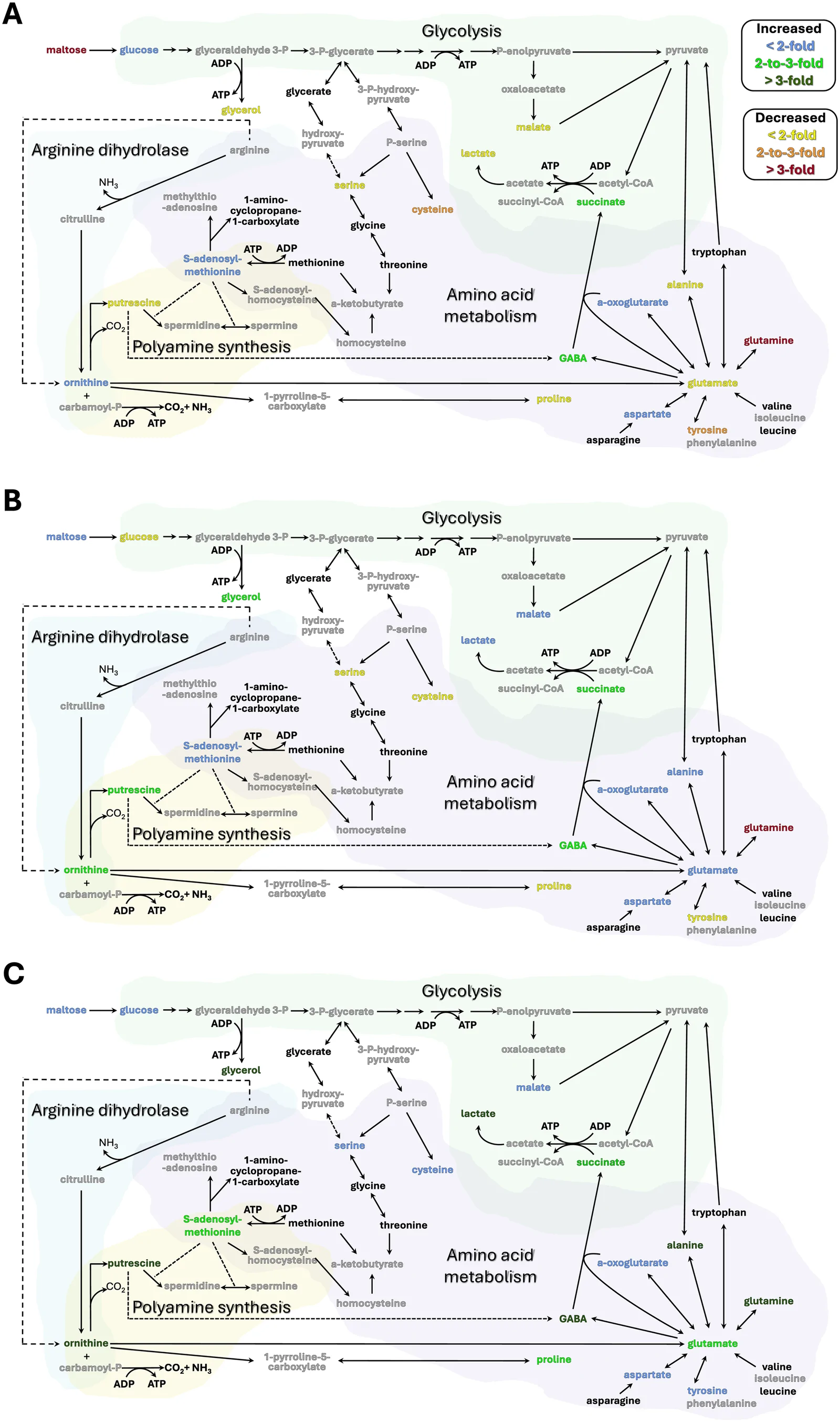
The integrative metabolic map depicting alterations in selected metabolites across glycolysis, amino acid metabolism, the arginine dihydrolase pathway, and polyamine synthesis. *T. vaginalis* was cultured with or without the BV-associated bacteria *P. bivia, A. vaginae* and *G. vaginalis*, as described in Fig 4, and the relative metabolite concentrations were captured by gas chromatography-mass spectrometry (GC-MS) at timepoints of 8h (A), 16h **(B)** and 32h (C). Color-coded metabolites represent increase (from blue to light and dark green) or decrease (from yellow to orange and red) in their relative concentrations between conditions. Metabolites that were not detected in our analysis are shown in gray, otherwise in black (except for ADP, ATP, CO_2_ and NH_3_, not captured by the GC-MS). Arrows indicate connections between enzymatic reactions known for the interconversion of these metabolites, with dotted arrows pointing to connections which enzymes may not exist in *T. vaginalis* based on genome annotation.

In mixed cultures, we detected the accumulation of glycolytic intermediates across the time course, such as malate and succinate, alongside lactate, a major end-product of glucose fermentation in these organisms (Fig 5). Elevated polyamine synthesis, as indicated by the accumulation of putrescine, is thought to counterbalance the acidification caused by lactate production [7]. We also observed accumulation of glycerol, a key intermediate linking sugar and fatty acid metabolism. Glycerol is an unusual metabolite in glycolysis for most eukaryotes, with trichomonads being one notable exception [33].

The most pronounced changes, however, were observed in amino acid metabolism which were consistent across different metabolites and incremental with time overall (Fig 5). This observation suggests a metabolic shift - or shunt - toward amino acid metabolism in the mixed cultures, which may reflect a cooperative metabolic strategy employed by *T. vaginalis* and BVAB to support their mutualistic growth. Corroborating this hypothesis, we found putrescine and ornithine as the top two metabolites showing the greatest fold difference between conditions (S1 and S2 Tables), and they accumulated by up to 3.3- and 15-fold respectively in mixed cultures (Fig 6). These metabolites bridge the arginine dihydrolase (ADH) and polyamine synthesis pathways. An increased flux through arginine metabolism, with similarly high levels of these two metabolites, has been reported when *T. vaginalis* is associated with its endosymbiont *M. hominis* [32]. The ADH pathway can generate ATP through carbamate kinase, suggesting that enhanced arginine metabolism may contribute to the energetic adaptation of co-cultures under nutrient-restricted conditions.

**Fig 6:**
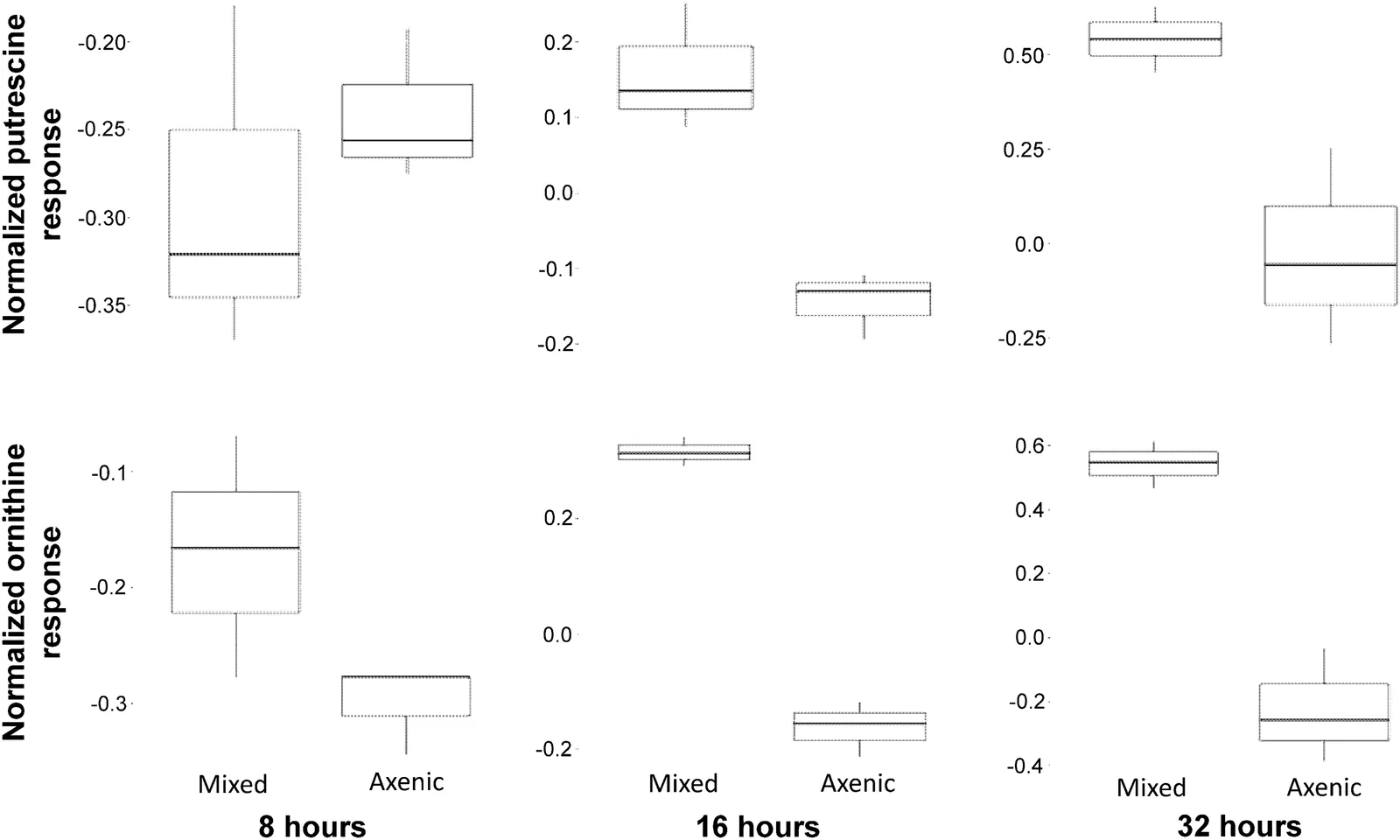
Accumulation of putrescine and ornithine during co-growth of *T. vaginalis* and BV-associated bacteria. Box plots represent normalized metabolite responses captured by GC-MS from mixed cultures of *T. vaginalis* with the BV-associated bacteria *P. bivia*, *A. vaginae* and *G. vaginalis*, compared to axenic cultures of *T. vaginalis* under the same serum-free defined media conditions as in Fig 4. The top and bottom panels show putrescine and ornithine respectively captured at 8h, 16h and 32h of anaerobic incubation. Both metabolites exhibited time-dependent accumulation in mixed cultures relative to axenic cultures, with putrescine and ornithine reaching ~3.3- and ~15-fold higher levels, respectively. Box plots show the median and interquartile range, with whiskers extending to the most extreme non-outlier values within 1.5 × the interquartile range.

### Other amino acid-derived metabolites accumulate during microbial co-growth

In addition to the metabolites displayed in the integrative metabolic map (Fig 5), several other metabolites of interest were found in altered concentrations in co-cultures of *T. vaginalis* and BVAB (S2 Data, and S1 and S2 Tables). Many of these metabolites are derived from amino acid catabolism and provide further insights into the metabolic interactions between these microorganisms. Hippurate, 2-hydroxybutyric acid (2HB), and 4-hydroxyphenylacetic acid (4-HPCA) are notable examples of amino acid-derived metabolites. Hippurate, a conjugate of benzoate and glycine, showed an inverse relationship with benzoate in our data, with the difference between the two increasing over time (S1 Table). This observation suggests that the conversion of hippurate to benzoate may be synergistically enhanced in mixed cultures. Hippurate hydrolysis has long been used as a diagnostic test for *Gardnerella vaginalis*. Consistent with our data, women with BV often exhibit sharply reduced levels of hippurate [34].

2HB is a metabolite produced by *G. vaginalis*, recognized as a biomarker of BV, that was shown to increase with the colonization of a cervicovaginal 3D tissue model by BV-associated bacteria [35,36]. In our data, 2HB levels increased steadily in co-cultures (S1 and S2 Tables) supporting its association with metabolic activity in dysbiosis. 4-HPCA is derived from the conversion of tyrosine, a reaction carried by many microorganisms. Although this enzymatic activity has not been reported for BVAB or *T. vaginalis*, 4-HPCA accumulated in mixed cultures (S1 Table). This metabolite is notable for its cytotoxicity to epithelial cells [37] as well as antimicrobial activity against lactobacilli [38] highlighting its potential role in dysbiosis and infection.

Gamma-aminobutyric acid or GABA, displayed in the integrative metabolic map (Fig 5), is synthesized from glutamate or putrescine by various microorganisms. GABA transaminase converts GABA and 2-oxoglutarate to glutamate and succinic semialdehyde, with the latter oxidized to succinate. *T. vaginalis* possesses the enzymatic machinery for this pathway and may utilize it to produce acetate and ATP in hydrogenosomes. While it is unclear if *T. vaginalis* can synthesize GABA from putrescine, intermediates of this pathway, such as N-acetylputrescine, are known to accumulate in women with BV suggesting that these bacteria may produce GABA via this pathway. N-acetylputrescine is associated with the typical amine odor and elevated pH of the BV vaginal discharge [7]. These findings suggest a cooperative mechanism where putrescine accumulation in co-cultures may be at least partially converted to GABA by BVAB, which could then be metabolized by *T. vaginalis* for energy production.

Lastly, a few metabolites containing aromatic rings derived from the catabolism of aromatic amino acids (e.g., phenylalanine, tyrosine and tryptophan) were found among metabolites which relative concentrations were altered between conditions (S1 and S2 Tables). In addition to benzoate and 4-HPCA, these include nicotinic acid, trans-cinnamic acid and 1-phenylethanol. As a precursor of NAD + , nicotinic acid or niacin may contribute to the cooperative metabolism of *T. vaginalis* and BVAB by help maintaining the NAD + /NADH redox balance. Trans-cinnamic acid and 1-phenylethanol, on the other hand, are volatile compounds produced via microbial secondary metabolism. These metabolites act as signalling molecules in microbial communities and exhibit antimicrobial activity [39,40].

### Arginine metabolism and polyamine synthesis contribute to mutual growth in protozoan–bacteria co-cultures

Considering the fluctuating environment of the urogenital tract, metabolic adaptability has long been thought to be an important physiological trait for the survival and pathogenicity of *T. vaginalis* [4,41]. Recently, glucose restriction was shown to reprogram *T. vaginalis* metabolism profoundly [42,43] while promoting pathogenicity [44]. Under glucose restriction, this parasite exhibits downregulation of general energy metabolism and upregulation of glutamate metabolism-related aminotransferases [43], alongside significant adaptive responses in hydrogenosomal amino acid metabolism [42]. The presence of the endosymbiont *M. hominis* has been shown to enhance arginine metabolism in *T. vaginalis*, improving energy generation and simultaneously reducing host nitric oxide production by macrophages [32]. Our metabolomics analysis suggests both upregulation of aminotransferases and enhancement of arginine metabolism during co-growth of protozoan and bacteria (Fig 5). Notably, the mixed cultures exhibited accumulation of ornithine and putrescine in a time-course manner (Fig 6) to levels comparable to those previously reported for *T. vaginalis* in association with its bacterial endosymbiont [32]. Therefore, these findings suggest that the ADH pathway may play a central role in supporting the mutualistic growth of *T. vaginalis* and BVAB.

Given these precedents, we sought to determine whether the observed changes in arginine metabolism result from direct bacteria-protozoa interactions rather than merely nutritional differences between the culture media (Fig 7). To address this, we examined the expression of key enzymes in the ADH pathway, a central metabolic route for arginine catabolism in *T. vaginalis*. This metabolic pathway converts arginine to ornithine and ammonia via arginine deiminase (ADI), ornithine carbamyltransferase (OCT) and carbamate kinase (CK) producing ATP (Fig 7A). After confirming that the most recent annotation of the *T. vaginalis* genome has homologues of these three enzymes, as previously reported [32], we specifically chose TVAG_261970 (Tv_CK1) as the single homologue of CK, and TVAG_041310 (Tv_OCT1) and TVAG_368740 (Tv_OCT2) as the two paralogues of OCT. For ADI, TVAG_344520 (Tv_ADI1) was chosen for being the only gene showing upregulation in response to glucose restriction [43].

**Fig 7:**
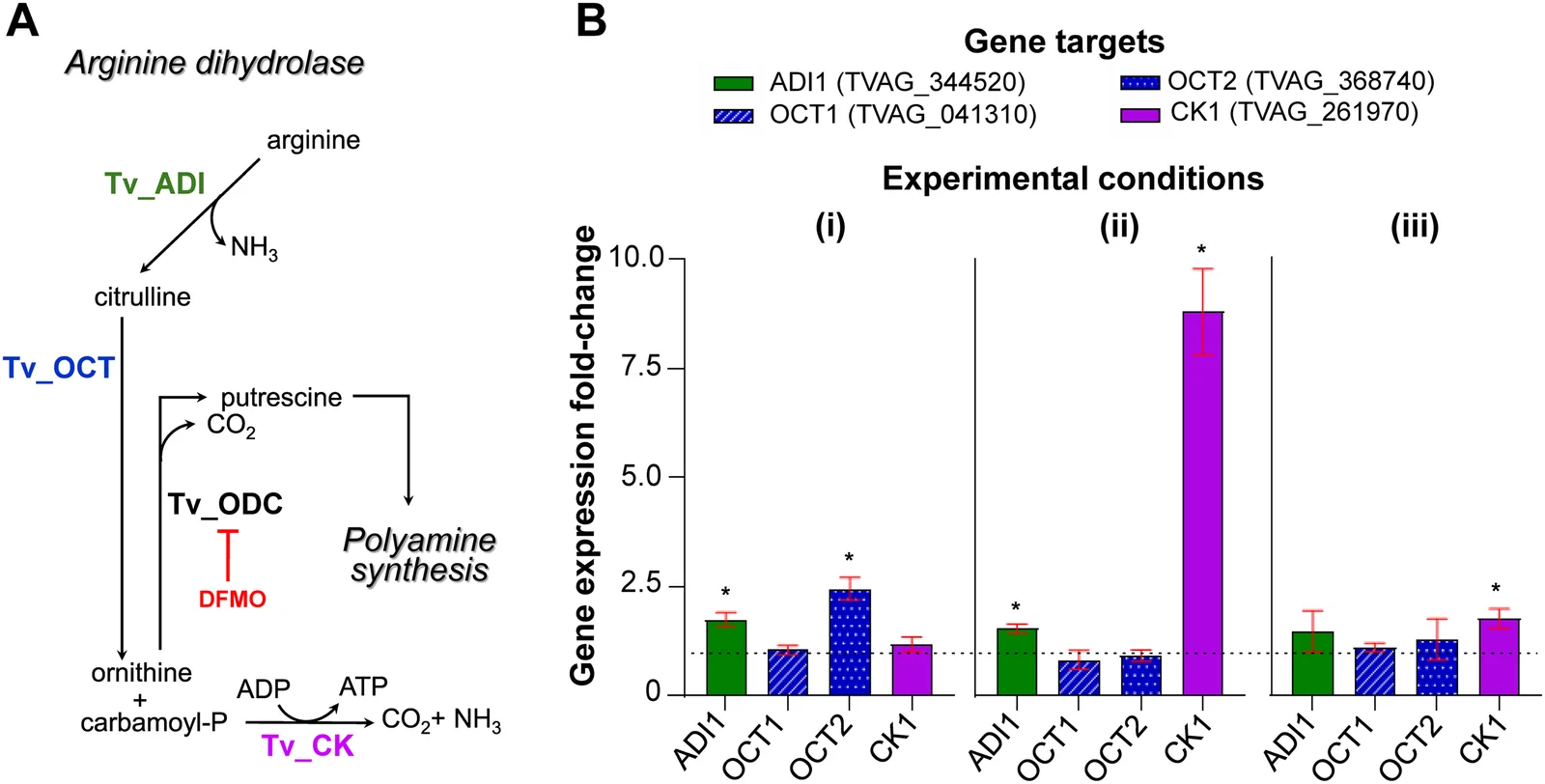
Relative expression levels of key arginine dihydrolase (ADH) pathway genes in *T. vaginalis* under different media and co-culture conditions. (A) Schematic representation of the ADH pathway in *T. vaginalis*, showing the conversion of arginine to citrulline and ammonia via arginine deiminase (ADI), followed by conversion to ornithine and carbamoyl-P by ornithine carbamyltransferase (OCT), and generation of ATP through carbamate kinase (CK). Ornithine can subsequently enter polyamine synthesis via ornithine decarboxylase (ODC), producing putrescine. Difluoromethylornithine (DFMO) inhibits ODC activity. Gene targets selected for expression analysis are color-coded as green, blue and purple. (B) Gene expression fold-change of Tv_ADI1 (TVAG_344520), Tv_OCT1 (TVAG_041310), Tv_OCT2 (TVAG_368740), and Tv_CK1 (TVAG_261970) after 8 hours of incubation comparing: (i) axenic *T. vaginalis* cultured in serum-free defined media relative to the optimal TYM medium; (ii) mixed cultures of *T. vaginalis* with BV-associated bacteria (BVAB) - *P. bivia*, *A. vaginae* and *G. vaginalis* - relative to axenic *T. vaginalis* cultures in serum-free DMEM; (iii) mixed cultures of *T. vaginalis* with BVAB relative to *T. vaginalis* axenic cultures in the optimal TYM medium. Bars represent mean + /- SD from six independent biological replicates. The dashed line indicates no-change 1-fold expression. Significant differences from 1-fold expression were assessed by two-tailed one-sample Student’s t-test; *p < 0.05.

Gene expression analysis of *T. vaginalis* enzymes was conducted to compare the responses to media and BVAB at 8 hours of incubation (Fig 7B). Modest upregulation of Tv_ADI1 and Tv_OTC2 was observed in *T. vaginalis* incubated axenically in DMEM compared to TYM media, indicating some metabolic response to the nutrient limitations in DMEM (Fig 7B). The relative expression of *T. vaginalis* ADH enzymes in mixed versus axenic cultures in DMEM was contrastingly different to the response to the nutrient limitations of this medium *per se*. Culturing *T. vaginalis* with BVAB induced a dramatic upregulation of the expression of Tv_CK1 (Fig 7B). This finding indicates that protozoan-bacteria interactions drive differential expression of these genes in *T. vaginalis*. Conversely, when *T. vaginalis* was incubated in its optimal TYM medium, presence or absence of BV-associated bacteria did not produce significant changes on the expression of these enzymes other than a modest upregulation of Tv_CK1 (Fig 7B). In conclusion, these findings together indicate that nutrient availability and BVAB distinctly modulate the expression of ADH enzymes in *T. vaginalis*. Most importantly, the massive upregulation of Tv_CK1 in mixed culture under defined media – a condition which neither microorganism can thrive or grow alone (Fig 4) - indicates that this pathway and enzyme might play a central role in supporting the co-growth of *T. vaginalis* and BVAB.

The ADH pathway generates ATP and ornithine, with the latter being converted to various amino acids and putrescine. Our time-course metabolomics data showed that ornithine accumulates early, and putrescine accumulation follows before most of other amino acids (Fig 5), with both ornithine and putrescine reaching the highest increased levels among all metabolites (Fig 6). These observations suggest that ADH-linked metabolism and polyamine synthesis contribute to the joint metabolism and co-growth of *T. vaginalis* and BVAB in mixed cultures (Fig 4).

To test this hypothesis, we sought to interfere with theses pathways (Fig 8). Firstly, we attempted to knockout TV_CK1 for being a single-copy gene that is significantly upregulated in mixed cultures (Fig 7). We recover neomycin-resistant parasites when targeting TV_CK1 with gRNA1 or gRNA2 (S4 Table), or the control for the ferredoxin (Fd) gene, using CRISPR/Cas9 in every attempt. However, contrary to the Fd control, the Tv_CK1 locus was intact and not disrupted by neomycin (Neo) expression cassette (Fig 8A). Along with the detection of the Neo amplicon, this result suggests off targeting when employing CRISPR/Cas9 to knockout Tv_CK1. This gene was claimed to be essential in *Giardia lamblia* after RNA interference [45] potentially explaining the unsuccessful attempts in *T. vaginalis*.

**Fig 8:**
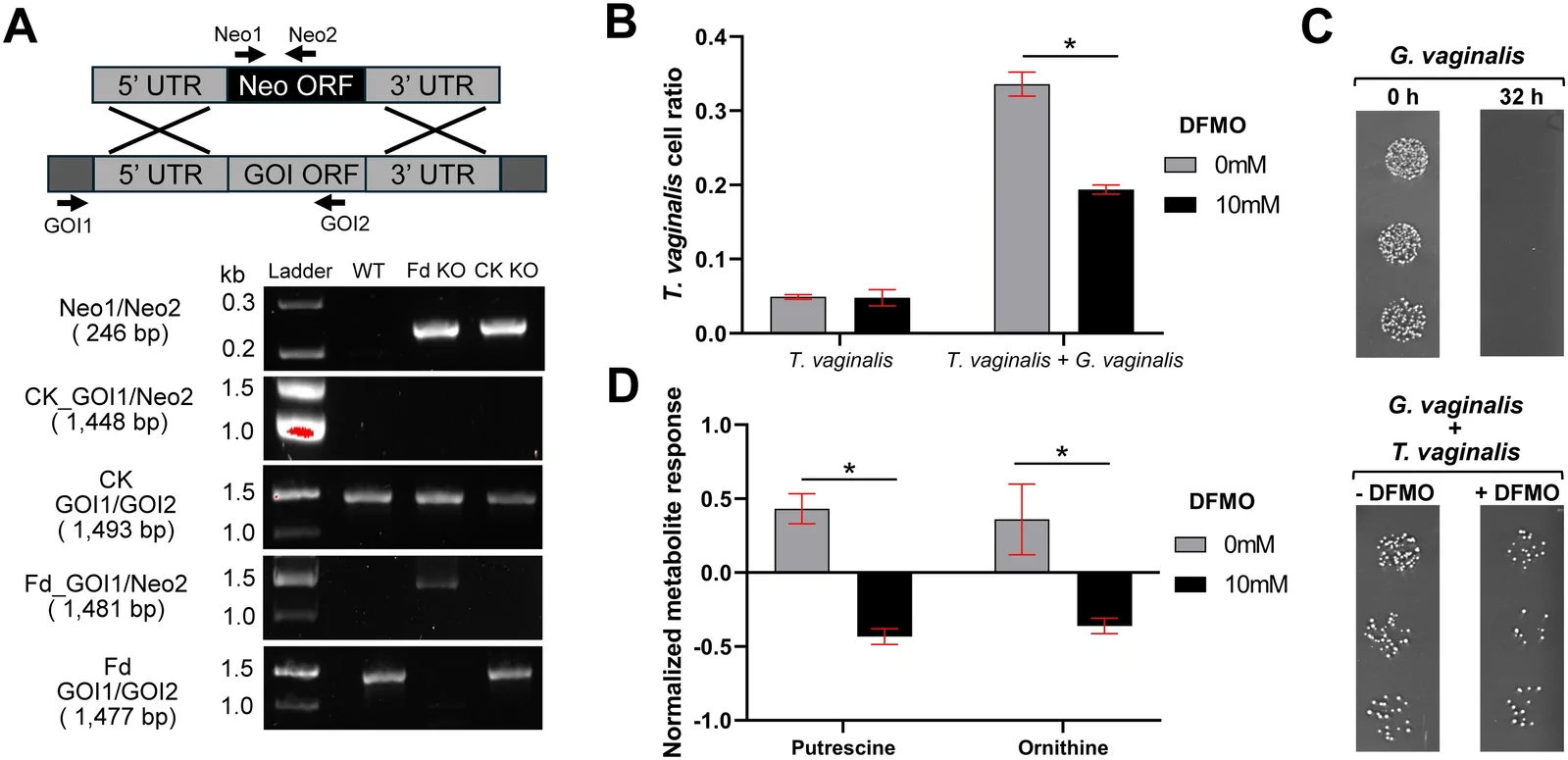
Functional interrogation of the arginine dihydrolase (ADH) and polyamine synthesis pathways during co-growth of *T. vaginalis* and the BV-associated bacterium *G. vaginalis.* (A) CRISPR/Cas9-mediated targeting of the gene of interest (GOI), either Tv_CK1 (TVAG_261970) or the control Tv_Fd (TVAG_003900). Top. Schematic representation depicting CRISPR-Cas9 mediated homologous recombination of the neomycin-resistance expression cassette (Neo) to disrupt the GOI locus. Bottom. Diagnostic PCRs for detection of gene knockout in transfected (Fd KO and CK KO) and wild-type non-transfected parasites (WT). Primers were positioned on the open reading frame (ORF) of the GOIs or adjacent of integration cassette, i.e., upstream of the ~ 1 kb-long 5’ untranslated region (UTR) used for guiding homologous recombination (S4 Table). PCR analysis after gel electrophoresis shows that Tv_Fd, but not Tv_CK1, was successfully knocked out. **(B)** Effect of difluoromethylornithine (DFMO) on *T. vaginalis* growth in axenic and in mixed culture with *G. vaginalis* in serum-free DMEM, with an initial inoculum of 5 x 10^5^ protozoal cells per ml. *T. vaginalis* viability was assessed using the TC20 automated cell counter, and parasite enumeration was performed by flow cytometry, as described in Materials and Methods. *T. vaginalis* growth was observed only in mixed culture, and was significantly reduced in the presence of 10 mM DFMO. Bars represent mean + /- SD from at least three independent biological replicates. Statistical significance was assessed by two-way ANOVA followed by Sidak’s multiple-comparisons test (*p < 0.05). **(C)** Effect of DFMO on *G. vaginalis* recovery in serum-free DMEM. Spot plating of three 2-fold dilutions of *G. vaginalis* grown alone or in mixed culture with *T. vaginalis* (5 x 10^5^ protozoal cells per ml) was performed as a qualitative readout of bacterial recovery and growth. In the absence of *T. vaginalis*, *G. vaginalis* population crashed becoming virtually undetectable after 32 h of incubation (top). In mixed culture with *T. vaginalis*, *G. vaginalis* growth was visually reduced in the presence of 10 mM DFMO (bottom). **(D)** Normalized metabolite responses for putrescine and ornithine in supernatants from *T. vaginalis*-*G. vaginalis* mixed cultures treated with 0 or 10 mM DFMO. Metabolites were analysed following MCF derivatization and GC-MS. DFMO treatment reduced the abundance of both putrescine and ornithine, supporting pharmacological perturbation of the ornithine-putrescine axis. Bars represent mean + /- SD from three independent biological replicates. Statistical significance was assessed by two-way ANOVA followed by Sidak’s multiple-comparisons test comparing 0 mM and 10 mM DFMO within each metabolite (*p < 0.01).

Lastly, we tested the necessity of polyamine supply on protozoa-bacteria interactions by treating cultures with difluoromethylornithine (DFMO) (Figs 8B–D). DFMO irreversibly inhibits ornithine decarboxylase (ODC) blocking polyamine biosynthesis (Fig 7A). ODC is the first and rate-limiting enzyme in the polyamine biosynthesis pathway converting ornithine to putrescine, the top two metabolites showing the greatest fold difference between conditions (S1 and S2 Tables, and Figs 5 and 6), suggesting that polyamine synthesis is highly enhanced in mixed cultures.

Treatment with DFMO did not impact the final number of *T. vaginalis* cells when grown axenically in its optimal medium; however, its growth was impacted by DFMO from mixed cultures in serum-free DMEM with a notable ~40% reduction in the final cell number at 32h (Fig 8B). Concomitantly, while *G. vaginalis* populations crashed in the absence of *T. vaginalis* after 32h of incubation as previously observed (Fig 4), DFMO also had a noticeable impact on the growth of this bacterium from mixed cultures in serum-free DMEM (Fig 8C). To confirm that DFMO perturbed the targeted metabolic axis under these co-culture conditions, we analysed supernatants from the mixed cultures by GC-MS following MCF derivatization. DFMO significantly reduced the normalized metabolite responses of both putrescine and ornithine in *T. vaginalis-G. vaginalis* co-cultures (Fig 8D), supporting inhibition of the ornithine-putrescine branch associated with polyamine biosynthesis (Fig 7A).

## Discussion

Microbial interactions are key determinants of ecological fitness, community structure, and disease outcome across diverse host environments [46,47]. Rather than acting in isolation, many microorganisms rely on metabolic exchanges with co-inhabiting species to overcome nutritional limitations, tolerate environmental stress, and colonize host niches [48–50]. Protozoa, including both commensals and parasites, are increasingly recognized as important players of the human microbiome [9,51]. The protozoan parasite *T. vaginalis* frequently coexists with dysbiotic bacterial communities characteristic of bacterial vaginosis (BV), as repeatedly reported by epidemiological and clinical studies [4]. However, the mechanistic basis underlying this association remains poorly understood.

In this study, we show that *T. vaginalis* and BV-associated bacteria (BVAB) engage in metabolic cooperation promoting mutual growth under nutrient conditions that do not support the proliferation of either partner alone. By integrating co-culture experiments, metabolomics, gene expression analysis, and targeted pathway perturbation, we identify a coordinated metabolic shift toward amino-acid catabolism, with the arginine dihydrolase (ADH) pathway and downstream polyamine synthesis as central components of this interaction. Together, these findings provide experimental evidence that cross-kingdom metabolic cooperation can directly support the survival and growth of polymicrobial communities relevant to trichomoniasis and vaginal dysbiosis. By promoting the persistence of both the parasite and dysbiosis-associated bacteria, this interaction provides a plausible ecological mechanism through which microbial cooperation may influence inflammatory potential, infection chronicity and recurrence.

A defining observation of this study is the ‘no growth alone, growth together’ phenotype observed in serum-free defined media. While *T. vaginalis* populations remained stable and viable but did not proliferate axenically, BVAB populations declined rapidly in the same medium. In sharp contrast, when in co-culture, both microbial partners proliferated, indicating that their coexistence alleviates nutritional or energetic constraints that neither organism is capable to overcome alone. Importantly, this metabolic compatibility showed ecological specificity: cooperative growth was reproducible across core BV-associated taxa, being somewhat redundant and with additive effects, but not observed with the non-dysbiotic vaginal bacterium *L. gasseri*. These findings provide functional support for a metabolic basis underlying the long-recognized epidemiological association between bacterial vaginosis and trichomoniasis, suggesting that protozoan and dysbiotic bacterial communities may directly support each other’s persistence in the vaginal niche.

This phenotype is characteristic of syntrophic interactions, which are common in anaerobic ecosystems where partners exchange metabolites to enable otherwise unfavorable or limiting reactions [52]. Cooperative metabolism among BV-associated taxa has long been proposed, exemplified by *G. vaginalis*-*P. bivia* partnership in which ammonia exchange might support bacterial growth and community expansion [53]. BV is now understood as a polymicrobial state structured by biofilm formation, where *Gardnerella* acts as an early colonizer facilitating persistence of additional anaerobes. In this context, our findings suggest that *T. vaginalis* could integrate into these metabolically interdependent bacterial communities, with dysbiosis and infection potentially reinforcing each other.

Furthermore, metabolomic analyses indicate that the *T. vaginalis*-BVAB association profoundly re-shapes the extracellular metabolic environment. Mixed cultures consistently accumulated metabolites derived from amino-acid catabolism and amine production, most notably ornithine and putrescine. These metabolites are directly connected to the ADH pathway, in which carbamoyl phosphate is converted into ATP via carbamate kinase (CK), while ornithine can be further converted to glutamate or decarboxylated to putrescine. As a central hub in amino-acid metabolism, glutamate can feed into the GABA shunt, linking amino-acid degradation to anaerobic energy generation.

Accordingly, gene expression analyses revealed CK as an important gene likely contributing to this mutual growth phenotype. Although mixed cultures appear to recapitulate features of a low-carbohydrate and high-amino-acid metabolic state [42,43], CK was strongly responsive to BVAB and not solely to differences in nutrient availability. Despite unsuccessful attempts to disrupt the CK gene using CRISPR/Cas9, this observation suggests that this enzyme may have a central role further supporting the ADH pathway as a key energetic axis in this microbial interaction. Given the extensive gene family expansion in *T. vaginalis*, this analysis was limited to selected ADH paralogues and did not capture other ADH/polyamine gene components or bacterial transcriptional responses. Future work using dual RNA-seq or meta-transcriptomics would enable simultaneous profiling of parasite and bacterial gene expression, providing a systems-level view of coordinated pathway regulation during cooperative growth.

The connection between ADH metabolism and polyamine synthesis further highlights the metabolic integration of this microbial partnership. Polyamines are ubiquitous metabolites required for nucleic-acid stabilization, ribosome function, stress tolerance and cell proliferation in bacteria and eukaryotic microbes [54]. Our observation that mixed cultures in serum-free media accumulated extracellular putrescine, together with the selective inhibition of co-culture growth by DFMO, strongly supports the idea that polyamines function as shared metabolic currency within this consortium.

While the contribution of CK-mediated ATP generation remains solely inferred from pathway structure and Tv_CK1 transcriptional upregulation, the reduction of putrescine following DFMO treatment further supports the functional involvement of the ornithine-putrescine branch in this microbial interaction. Polyamine biosynthesis is tightly linked to ADH-derived ornithine in *T. vaginalis*, and BVAB are known producers of biogenic amines and polyamines, which accumulate during dysbiosis and contribute to pH elevation and community restructuring [2,7]. Inhibition of ornithine decarboxylase (ODC) by difluoromethylornithine (DFMO) has long been shown to reduce intracellular polyamine pools and impair *T. vaginalis* growth under nutrient-limited conditions [55]. Therefore, our findings suggest that polyamines become critical when *T. vaginalis*-BVAB metabolic relationships emerge in the nutrient-defined condition.

In this context, the metabolic partnership established between *T. vaginalis* and its endosymbiont *M. hominis* provides a useful parallel [31,32]. In this association, both organisms exploit the ADH pathway to generate ATP under anaerobic conditions, similarly leading to enhanced ornithine and putrescine production in *M. hominis*-associated parasites. This shared metabolism has been proposed to increase parasite fitness and reshape nitrogen and polyamine flux in the vaginal niche. Recent work has shown that both *M. hominis* and “*Candidatus Mycoplasma girerdii*” can influence *T. vaginalis* pathophysiology [56], reinforcing the importance of defining the symbiont status of parasite strains used in mechanistic studies. The similarities observed here suggest that ADH-linked energetics and polyamine metabolism represent recurring metabolic nodes through which *T. vaginalis* integrates bacterial partnerships, extending from intracellular bacterial symbionts to extracellular polymicrobial communities.

Beyond central metabolism, our analysis revealed secondary metabolites that might be relevant to reshaping the vaginal niche in support of dysbiosis. These include reciprocal changes in hippurate and benzoate, steady accumulation of 2-hydroxybutyrate (2HB) and 4-hydroxyphenylacetic acid (4-HPCA). The microbiota contributes to the accumulation of 2HB, a metabolic marker of proteolytic and redox-stress states that is associated with multiple disease contexts, including metabolic disorders and cancers [57]. The dysbiosis-associated bacterium *Fusobacterium nucleatum* promote an increase of 2HB levels in the colon [58]. Consistent with this, 2HB has been identified among metabolomic signatures associated with increased vaginal bacterial diversity and BV-like states [59] and has also been linked to redox adaptation in cellular proliferation driven by high-risk human papillomaviruses [60].

The accumulation of glycerol may represent an additional mechanism for redox balancing during cooperative growth. Glycerol has been identified as a major fermentation product of *T. vaginalis*, formed from glycolytic triose phosphates to support redox balance [33]. This strategy allows excess NADH to be re-oxidized, maintaining glycolytic flux and continued ATP generation through substrate-level phosphorylation. In *Entamoeba histolytica,* a protozoan parasite of the intestinal mucosa, oxidative stress redirects glycolytic flux towards glycerol production [61] and functional studies demonstrate that glycerol metabolism is essential for parasite proliferation and oxidative-stress defence [62]. Together, these observations suggest that glycerol production may represent an emerging metabolic strategy among anaerobic protozoa lacking canonical antioxidant systems, supporting ATP generation, growth, and the cellular antioxidant response.

Taken together, concordant metabolite signatures allow us to infer that *T. vaginalis* and BVAB may engage in a syntrophic interaction. Since the G3 strain used here carry *M. hominis*, we cannot exclude that this endosymbiotic association may also have had an impact on the cooperative growth phenotype observed in this study. Future work using isogenic *Mycoplasma*-free and *Mycoplasma*-associated parasite lines will be needed to determine whether the endosymbiont could contribute additively, redundantly, or independently to the metabolic cooperation described here. Because *T. vaginalis* has been shown to phagocyte bacteria [63,64], it would be also interesting to investigate whether bacterial internalization via phagocytosis could contribute to the metabolic phenotype observed in co-cultures.

Furthermore, our experiments cannot assign the cellular origin of individual metabolites or directly measure bidirectional metabolite exchange or partner-specific fluxes. Future work would be needed to address these limitations, such as employing stable-isotope tracing and isotope-resolved metabolomics or single-cell stable-isotope probing approaches (e.g., NanoSIMS or Raman microspectroscopy) to track carbon and nitrogen transfer between partners and resolve species-specific metabolic fluxes in co-culture [65–68].

Notably, these experiments were performed in a simplified co-culture system that does not reproduce the full complexity of the vaginal environment. In particular, this system does not include host epithelial cells, immune mediators, mucus, or the fluctuating physicochemical conditions of the vaginal niche. While ex vivo models of the vaginal mucosa may help address some of these limitations, in vivo validation remains challenging because available animal models do not fully recapitulate the distinctive low-pH, *Lactobacillus*-associated ecology of the human vagina.

In conclusion, our study supports a model in which *T. vaginalis*-BVAB mixed cultures give rise to a high-flux, nutrient-limited and redox-challenged metabolic state. Under these conditions, central carbon metabolism appears to be rewired away from carbohydrate reliance and towards amino-acid catabolism linked to anaerobic energy metabolism, accompanied by increased glycerol production as a redox sink and with the accumulation of 2HB as a marker of oxidative stress. Concurrent changes in polyamine metabolism further suggest enhanced demands for growth and stress tolerance within this consortium.

While many previous studies on interactions between *T. vaginalis* and vaginal bacteria, including those from our group, have focused on changes in pathogenicity [4,22,23,25,27,28], the present study highlights how the parasite and dysbiotic bacteria can rewire their metabolism to overcome nutritional and redox limitations that neither partner can overcome in isolation. These findings underscore the importance of viewing infection as an ecological process emerging from host-microbiome-pathogen interactions. In the context of the vaginal ecosystem, our results suggest that metabolic cooperation may help stabilize dysbiosis and promote persistence of both parasite and bacteria, with implications for disease risk, treatment response, and recurrence.

## Materials and methods

### Microbial cultivation and growth assessments

*Trichomonas vaginalis* - reference strain G3 - was maintained axenically at 37 °C in TYM medium pH 6.2 [69] supplemented with 10% heat-inactivated horse serum, 10 U/mL penicillin, and 10 μg/mL streptomycin (Thermo Fisher, New Zealand) under static and microaerophilic conditions. *T. vaginalis* viability was assessed during cultivation both axenically and in co-cultures with bacteria using the TC20 automated cell counter, as per manufacturer’s recommendation (Bio-Rad). The working stock of the G3 strain used in this study, along with DNA from other strains, were screened by PCR and Sanger sequencing for *Mycoplasma hominis* and “*Candidatus Mycoplasma girerdii*” using the previously reported primers (Mh16S2S/Mh16SAS and Mg16S-426F/Mg16S-736R) and conditions [70]. The dsRNA virus (TVV) status of the working stock was not determined in this study, although TVV-positive G3 stocks have been reported previously [71]. *T. vaginalis* was recovered from frozen stocks, passaged daily without antibiotics for a minimum of three days prior to mixing in cultures with bacteria.

*Atopobium vaginae* (ATCC BAA-55), *Gardnerella vaginalis* (ATCC 14018) and *Prevotella bivia* (ATCC 29303) were revived, following ATCC guidelines from freeze-dried microorganisms, and cultured axenically in NYC-III medium. *Lactobacillus gasseri* (ATCC 9857) was grown axenically on de Man, Rogosa and Sharp (MRS; BD Biosciences). Both media were supplemented with 10% horse serum, and all bacteria were grown at 37 °C. *L. gasseri* was grown under static and microaerophilic conditions for 20–24 h, and the other three bacteria were grown in a Coy anaerobic chamber for 36–48 h. Bacterial cell counts and viability were determined by flow cytometry, as previously described [28], prior to mixing in cultures with *T. vaginalis*.

For co-culture experiments, *T. vaginalis* cells were pre-stained with CellTracker Orange CMTMR as previously described [72]. Pre-stained *T. vaginalis* and unstained bacteria were co-incubated either in serum-supplemented TYM medium or serum-free Dulbecco’s Modified Eagle’s Medium pH 7.2 (DMEM, Thermo Fisher, New Zealand) without antibiotics, as specified for each experiment. Pre-stained *T. vaginalis* and unstained bacteria were mixed at a 1:10 ratio and diluted to the indicated concentrations in either medium, as specified for each experiment. When multiple bacterial species were used, the total bacterial inoculum represented the combined population divided equally among the indicated species. *T. vaginalis* axenic cultures were prepared simultaneously in identical conditions, and both axenic and mixed cultures were grown in multi-well plates which were incubated in a Coy anaerobic chamber at 37 °C. At the end of the incubation periods, *T. vaginalis* cells were enumerated by flow cytometry based on fluorescence-positive events and FSC/SSC properties, as previously optimized and validated for *T. vaginalis* detection on a BD FACSCalibur (BD Biosciences) flow cytometer [72] and applied in previous *T. vaginalis*-bacteria co-incubation studies [23,28]. Where indicated, bacterial growth was qualitatively assessed by spotting 3 ml of culture dilutions onto NYC-III agar plates and incubated anaerobically for 24-48h. Spot plating was used as a qualitative assessment of bacterial survival and growth, as previously applied in *T. vaginalis*-bacteria interaction studies [3,29]. Experiments were performed in a minimal of three independent biological replicas, and one- or -two way ANOVA was employed to compare the mean *T. vaginalis* cell numbers between mixed and single cultures.

### Metabolomics, data processing and analysis

Culture supernatants from single and mixed microbial cultures were collected at 8, 16, and 32 hours of incubation. A volume of 0.6 mL from each sample was centrifuged, and the clarified supernatant was stored at -80 °C until analysis. Metabolites were derivatized using two complementary chemical derivatization methods: methyl chloroformate (MCF) and trimethylsilyl (TMS), targeting a broad range of amino acids, organic acids, alcohols, and amines, as previously described [73,74]. For MCF derivatization, 20 mL of 10 mM 2,3,3,3-d_4_-DL-alanine was added to each sample as an internal standard prior to derivatization, while for TMS derivatization, 20 µL of a 0.5 mg/mL solution of Ribitol was used as the internal standard. The MCF protocol followed a previously published procedure [75], with minor modifications: freeze-dried samples were resuspended in 1% NaOH, mixed sequentially with pyridine, methanol, and MCF, and extracted into chloroform following sodium bicarbonate addition. For TMS derivatization, freeze-dried samples were extracted in methanol, dried, and treated sequentially with methoxyamine hydrochloride in pyridine and N-methyl-N-(trimethylsilyl)trifluoroacetamide (MSTFA). Gas chromatography–mass spectrometry (GC-MS) analyses were performed using an Agilent 7890B GC system coupled to a 5977A mass selective detector. Derivatized samples (1 mL) were injected in splitless mode onto a ZB-1701 column (Phenomenex), with oven temperature programs optimized for each derivatization method. Mass spectra were acquired in scan mode (38–550 m/z) under electron impact ionization at 70 eV. Metabolite identification and deconvolution were performed using the automated mass spectral deconvolution and identification system or AMDIS software and an in-house mass spectral library, with retention indices and reference ions confirmed against standards where available, as done previously [74,76].

Raw GC-MS spectra from MCF- and TMS-derivatized samples were processed using an in-house R package, MassOmics, for automated peak integration and preliminary metabolite identification against dedicated in-house spectral libraries for each derivatization method [73,74]. For TMS data, a subset of the 2014 National Institute of Standards and Technology (NIST) library was additionally employed to complement compound identification not represented in the in-house database. Peak integrations were manually curated for retention time shifts, with false positives and duplicate entries removed as previously described [74,76]. Metabolite peak responses were normalized to internal standards to correct for technical variation in sample preparation [74]. Pre-processing involved log transformation and range scaling of metabolite responses. Compounds with low variability across conditions (i.e., relative standard deviation or RSD > 20%) were considered uninformative and excluded from downstream analyses. Exploratory data analysis was performed via principal component analysis (PCA) using both MetaboAnalyst (http://www.metaboanalyst.ca) and MassOmics, following metabolomics workflows previously applied in related studies [74]. Metabolites showing the largest fold-differences between mixed and axenic cultures at each timepoint were identified and ranked, and those with the most consistent differences were selected as key metabolites for biological interpretation. Pathway mapping of discriminant metabolites was conducted using information from the KEGG database and available metabolic maps for *T. vaginalis* and bacteria, alongside relevant literature. An integrative pathway diagram was manually assembled based on this mapping, incorporating metabolite fold-difference data to visualize metabolic shifts between experimental conditions. Full analytical workflows and quality control metrics for both MCF and TMS datasets are detailed (S1 Data).

### Gene expression analysis

Gene expression analyses were performed on *T. vaginalis* cultures after 8 h of incubation under the following three experimental conditions: axenic *T. vaginalis* in serum-free DMEM or in its optimal TYM medium, and mixed cultures of *T. vaginalis* with BV-associated bacteria (*P. bivia*, *A. vaginae*, *G. vaginalis*) in serum-free DMEM. Total RNA was extracted with TRIzol reagent (Thermo Fisher Scientific) according to the manufacturer’s instructions after cells were harvested washed in PBS by centrifugation (3,000 × g, 20 min, 4 °C). To remove genomic DNA contamination, RNA samples were subjected to LiCl precipitation followed by DNase I digestion (NEB) according to the manufacturer’s protocol. Following phenol-chloroform purification and ethanol precipitation, RNA was resuspended in RNase-free water and quantified using a NanoPhotometer (Implen).

First-strand cDNA synthesis was performed using 500 ng of purified RNA and SuperScript IV Reverse Transcriptase (Thermo Fisher Scientific) and poly(d)T primer, following the manufacturer’s instructions. Reverse transcriptase-negative reactions were included as controls for residual genomic DNA contamination, and this was assessed along with primer specificity by conventional end-point PCR and agarose gel electrophoresis prior to digital droplet PCR (ddPCR). Expression of arginine dihydrolase (ADH) pathway genes was quantified by ddPCR using the QX200 Droplet Digital PCR system, following manufacturer’s recommendation (Bio-Rad). Target genes included Tv_ADI1 (TVAG_344520), Tv_OCT1 (TVAG_041310), Tv_OCT2 (TVAG_368740), and Tv_CK1 (TVAG_261970), and the housekeeping gene α-tubulin (TVAG_206890) was used as an internal normalizer (S3 Table).

ddPCR reactions were prepared using EvaGreen Supermix (Bio-Rad) with 150 nM final primer concentration and 1 µL of diluted cDNA template per reaction. Droplets were generated using the QX200 Droplet Generator, PCR amplification was performed under default EvaGreen ddPCR cycling conditions, and droplets were read using the QX200 Droplet Reader. Data were analysed using QuantaSoft Analysis Pro software (Bio-Rad) to obtain absolute transcript copy numbers (copies/µL). Expression ratios for each gene of interest (Ei) were calculated as Ei = (copies/µL in sample of interest) / (copies/µL in control sample). After the expression ratio of the normalizer (En) was calculated, fold-change in gene expression was expressed as Ei/En. Gene expression ratios were compared across the three experimental conditions. Experiments were performed with independent biological replicates (n = 6), and results presented as mean + /- standard error of the mean. Statistical significance was assessed using a two-tailed one-sample Student’s t-test comparing fold-change values against a theoretical mean of 1 (i.e., no change).

### Genetic and pharmacological approaches for interfering with metabolic pathways

CRISPR/Cas9-mediated gene disruption was performed following the original established system, which is based on co-nucleofecting parasites with a plasmid expressing FKBP-destabilized Cas9 and U6-driven transcription of gene-specific guide RNAs (gRNAs), and a homology-directed repair PCR amplicon consisted of the neomycin (Neo) resistance cassette flanked by ~1 kb of 5′ and 3′ untranslated regions (UTRs) corresponding to the target locus [77]. The ferredoxin gene (Tv_Fd-1; TVAG_003900) was used as a positive control [77], and the system was customized to target the single copy of *T. vaginalis* creatine kinase gene (Tv_CK1; TVAG_261970) using two gene-specific gRNAs (S4 Table). Nucleofection was perfomed three independent times for each gRNA. Neo-selected parasites underwent diagnostic PCR analyses to evaluate if gene locus was disrupted by the integration of the Neo resistance cassette. All gRNA target sequences, recombination-arm primers, and diagnostic PCR primers are listed (S4 Table).

To assess the contribution of polyamine biosynthesis to the mutual microbial growth, cultures were treated with difluoromethylornithine prepared in sterile water (DFMO; Abcam), an irreversible inhibitor of ornithine decarboxylase (ODC). DFMO was added at a final concentration of 10 mM at the time of inoculation of *T. vaginalis*, with or without *G. vaginalis*, in serum-free DMEM. Parallel untreated cultures (0 mM DFMO, water volume instead) served as controls. Incubation and microbial growth (*T. vaginalis* and *G. vaginalis*) proceeded as previously described at 32-hour post-incubation. Experiments were done in triplicates, and statistical analysis was performed using two-way ANOVA to evaluate the effects of the DFMO treatment on the mixed cultures.

## Supporting information

S1 Fig Diagnostic PCR for the detection of mycoplasmas in *T. vaginalis* cultures. Primers were designed to target the 16S rRNA gene of *M. hominis* and “*Ca. M. girerdii*”, as previously described [70], and *T. vaginalis* alpha-tubulin [3]. Water (H_2_O) or genomic DNA from strains G3 (this study), T1, B7RC2 and CDC1132 were used for amplification. The presence of *M. hominis* amplicons detected by gel electrophoresis was further confirmed by Sanger sequencing and BLAST analysis, which identified the G3 and CDC1132 sequences as *M. hominis* 16S rRNA, with 98.6–99.3% identity and 96–100% query coverage.
(TIF)

S2 Fig Principal component analysis of time-course metabolomics datasets derived from MCF and TMS derivatizations. Unsupervised principal component analysis (PCA) of GC-MS metabolite profiles from mixed (*T. vaginalis* with BV-associated bacteria) and axenic *T. vaginalis* cultures at 8 h, 16 h, and 32 h. Data were log-transformed and range-scaled prior to analysis. (A) PCA score plots from the MCF-derivatized dataset. (B) PCA score plots from the TMS-derivatized dataset. Variance explained by each principal component is indicated on the respective axes. Condition-dependent separation becomes evident at later timepoints, 16 h for MCF and 32 h for TMS, suggesting progressive metabolic differentiation during mutual microbial growth.
(PDF)

S1 Table Ranked metabolites from the MCF dataset showing the greatest fold-difference between mixed and axenic cultures across timepoints. (PDF)

S2 Table Ranked metabolites from the TMS dataset showing the greatest fold-difference between mixed and axenic cultures across timepoints. (PDF)

S3 Table Primer sequences used for digital droplet PCR (ddPCR) analysis of ADH pathway genes and reference gene. (PDF)

S4 Table gRNA target sequences and primer sets used for building Tv_CK1 homologous recombination cassette and for diagnostic PCR of CRISPR/Cas9 gene targeting. (PDF)

S1 Data GC–MS metabolomics data processing and exploratory analysis reports. This file details the analytical workflow, preprocessing, normalization, and exploratory analyses for the original time-course metabolomics experiment using TMS (Part A) and MCF (Part B) derivatization, including PCA quality assessment and metabolite boxplots. It also includes the additional MCF-only analysis of DFMO-treated and untreated cultures used to assess changes in putrescine and ornithine abundance (Part C).
(PDF)

S2 Data Normalized metabolite peak responses for MCF and TMS datasets across time-course and culture conditions. (PDF)

S1 Raw Images Uncropped gel images related to Fig 8A (A) and S1 Fig (B). (PDF)
