# Supplementary material for: Metabolic cooperation supports mutual growth between the protozoan parasite Trichomonas vaginalis and dysbiotic vaginal bacteria
Source: PLoS Pathog. 2026 Sep 23;22(9):e1014620. doi: 10.1371/journal.ppat.1014620 (PMC13626468; doi:10.1371/journal.ppat.1014620)
Supplement: Supplementary file 1 [file ppat.1014620.s002.pdf]

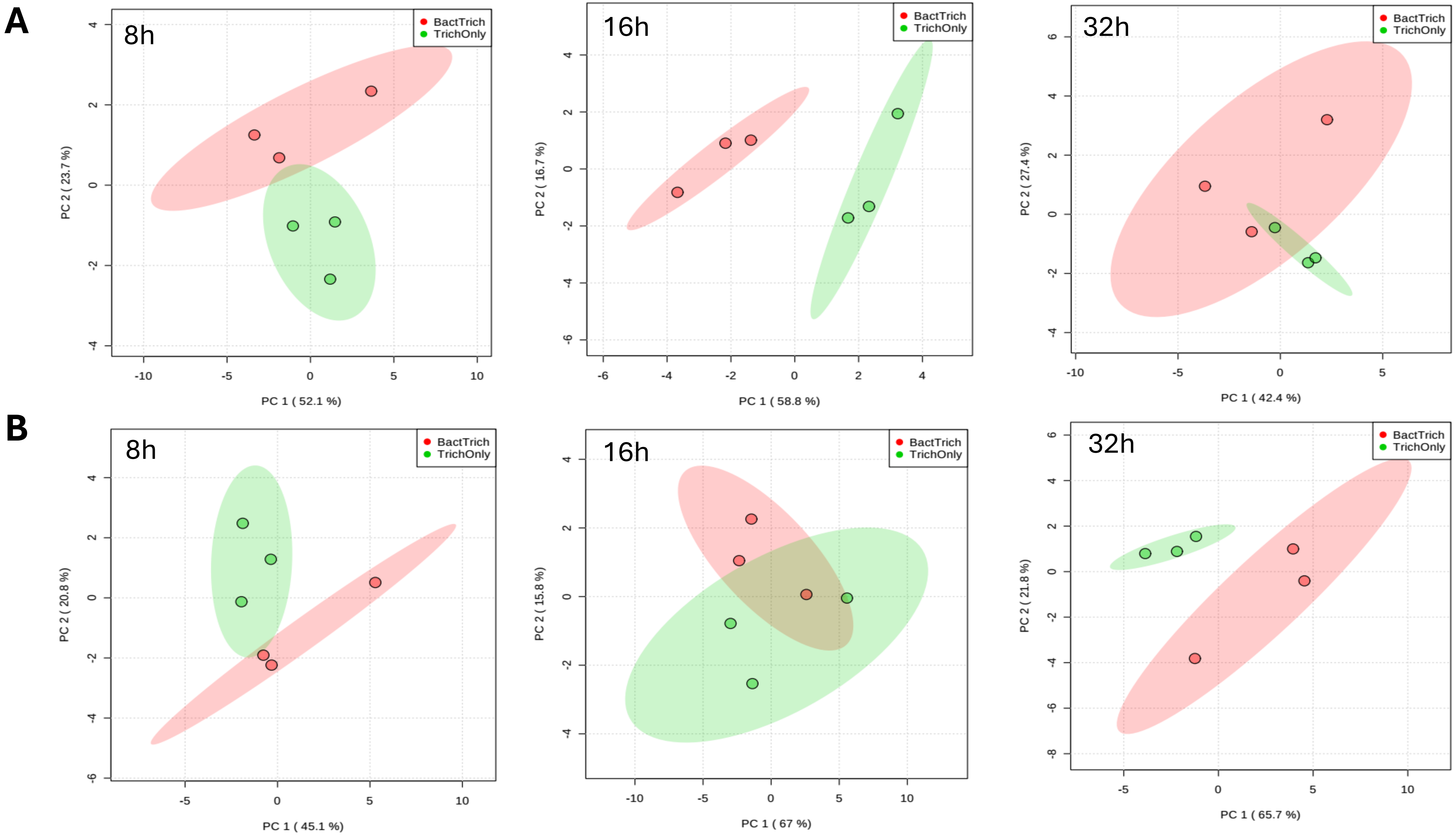
A
8h
BactTrich
TrichOnly
PC 1 (52.1 %)
PC 2 (23.7 %)
16h
BactTrich
TrichOnly
PC 1 (58.8 %)
PC 2 (16.7 %)
32h
BactTrich
TrichOnly
PC 1 (42.4 %)
PC 2 (27.4 %)
B
8h
BactTrich
TrichOnly
PC 1 (45.1 %)
PC 2 (20.8 %)
16h
BactTrich
TrichOnly
PC 1 (67 %)
PC 2 (15.8 %)
32h
BactTrich
TrichOnly
PC 1 (65.7 %)
PC 2 (21.8 %)
