# Supplementary material for: Metabolic cooperation supports mutual growth between the protozoan parasite Trichomonas vaginalis and dysbiotic vaginal bacteria
Source: PLoS Pathog. 2026 Sep 23;22(9):e1014620. doi: 10.1371/journal.ppat.1014620 (PMC13626468; doi:10.1371/journal.ppat.1014620)
Supplement: Supplementary file 2 [file ppat.1014620.s003.pdf]

Table S1. Ranked metabolites from the MCF dataset showing the greatest fold-differences between mixed and axenic cultures across timepoints. Values shown for individual samples are normalized GC-MS metabolite responses after methyl chloroformate (MCF) derivatization. 'AllM' indicates mixed cultures containing *T. vaginalis* and all three BV-associated bacteria; 'TM' indicates axenic *T. vaginalis* cultures. Numbers 8, 16 and 32 indicate incubation timepoints in hours, and letters a-c indicate biological replicates. 'allvstrich' represents the fold-change calculated as mixed culture / axenic *T. vaginalis*. 'trichvsall' represents the reciprocal comparison.

Name	AllM8a	AllM8b	AllM8c	TM8a	TM8b	TM8c	allvstrich	trichvsall	Foldchange8h
2-Hydroxybutyric acid	0.001249	0.001752	0.001370856	0.001317	0.001421284	0.001585211	1.011114	0.989008	1.011114
2-ketoglutamarate	0.006583	0.004415	0.003656094	0.004102	0.00385009	0.004362282	1.189957	0.840366	1.189957
2-Oxoglutaric acid	0.000584	0.000551	0.000425504	0.000358	0.000366241	0.000345203	1.458799	0.685495	1.458799
4-Aminobutyric acid (GABA)	0.000635	0.000557	0.000486971	0.000266	0.00018672	0.000183957	2.636552	0.379283	2.636552
4-Hydroxyphenylacetic acid	0.000964	0.001068	0.000816224	0.000759	0.000737282	0.000753543	1.265864	0.789974	1.265864
Aspartic acid	0.029897	0.030289	0.023412723	0.023411	0.023124539	0.023655523	1.191004	0.839628	1.191004
Benzoic acid	0.010137	0.010523	0.007321241	0.010483	0.006217456	0.008303052	1.119071	0.893599	1.119071
Creatinine	0.065701	0.06683	0.069712125	0.095294	0.094832883	0.091656977	0.717724	1.393292	-1.393292
Hippuric acid	0.021429	0.020345	0.015220938	0.019558	0.018996529	0.017856105	1.010356	0.98975	1.010356
Lactic acid	0.239604	0.219356	0.203342279	0.237708	0.215378471	0.211395349	0.996719	1.003292	-1.003292
Malic acid	0.000728	0.00067	0.000625152	0.000709	0.000628802	0.000649073	1.018313	0.982016	1.018313
Nicotinic acid	0.002034	0.002954	0.002311444	0.002083	0.001672804	0.002201126	1.225243	0.816164	1.225243
Ornithine	0.069238	0.08515	0.054514058	0.047319	0.05461646	0.054643895	1.33416	0.749535	1.33416
Putrescine	0.000214	0.000297	0.000191072	0.000288	0.000237601	0.000248456	0.9069	1.102657	-1.102657
S-Adenosylmethionine	0.006363	0.00521	0.00377188	0.004926	0.004916173	0.003929415	1.114279	0.897441	1.114279
Succinic acid	0.005515	0.005349	0.004930223	0.002646	0.002215686	0.002770258	2.069569	0.483192	2.069569
trans-Cinnamic acid	0.000107	0.000156	8.86268E-05	0.000128	8.16002E-05	7.04033E-05	1.255605	0.796429	1.255605

Name	All16a	AllM16b	AllM16c	TM16a	TM16b	TM16c	allvstrich	trichvsall	Foldchange16h
2-Hydroxybutyric acid	0.003446	0.004802466	0.00502795	0.002332201	0.002478	0.002506	1.81454	0.551104	1.81454
2-ketoglutamarate	0.004213	0.002578643	0.005108556	0.00353643	0.004374	0.00367	1.027557	0.973182	1.027557
2-Oxoglutaric acid	0.000645	0.000616351	0.000538887	0.00033632	0.000396	0.000384	1.613408	0.619806	1.613408
4-Aminobutyric acid (GABA)	0.000521	0.000441637	0.000609984	0.000234058	0.000329	0.000242	1.954438	0.511656	1.954438
4-Hydroxyphenylacetic acid	0.001573	0.002142937	0.001905123	0.001054361	0.001182	0.001264	1.605856	0.622721	1.605856
Aspartic acid	0.018026	0.018791414	0.01977307	0.016637046	0.017715	0.017747	1.086195	0.920645	1.086195
Benzoic acid	0.012796	0.012268774	0.0108357	0.008434891	0.008485	0.008148	1.432133	0.698259	1.432133
Creatinine	0.071165	0.086485367	0.084106191	0.094166102	0.05609	0.091818	0.998686	1.001316	-1.001316
Hippuric acid	0.009087	0.008219086	0.009517013	0.013953538	0.015447	0.015848	0.59278	1.686968	-1.686968
Lactic acid	0.251326	0.282095527	0.243479017	0.189488829	0.177763	0.196091	1.379092	0.725115	1.379092
Malic acid	0.000563	0.000554338	0.000509905	0.000536878	0.000641	0.000464	0.991773	1.008296	-1.008296
Nicotinic acid	0.005371	0.004669274	0.005926396	0.003492792	0.004429	0.003609	1.384777	0.722138	1.384777
Ornithine	0.183016	0.203789343	0.191398099	0.062542316	0.076624	0.070687	2.755266	0.362941	2.755266
Putrescine	0.000548	0.000801849	0.000612248	0.000332353	0.000349	0.000286	2.027173	0.493298	2.027173
S-Adenosylmethionine	0.004868	0.004972326	0.004958211	0.003936664	0.003932	0.003781	1.270251	0.787246	1.270251
Succinic acid	0.006266	0.006269197	0.005680954	0.002466641	0.00247	0.002544	2.435094	0.410662	2.435094
trans-Cinnamic acid	0.000117	4.09822E-05	5.93228E-05	7.05258E-05	0.000123	0.000112	0.711956	1.404581	-1.404581

Name	AllM32a	AllM32b	AllM32c	TM32a	TM32b	TM32c	AllvsTrich	trichvsAll	Foldchange32h
2-Hydroxybutyric acid	0.018617302	0.011239682	0.009577396	0.002956	0.006076	0.001991643	3.577148	0.279552	3.577148
2-ketoglutamarate	0.012997399	0.010237323	0.007300437	0.004585	0.005283	0.002671616	2.435172	0.410649	2.435172
2-Oxoglutaric acid	0.000455326	0.00050901	0.00034711	0.000382	0.000408	0.000358475	1.142145	0.875546	1.142145
4-Aminobutyric acid (GABA)	0.000617354	0.000511313	0.000351158	0.000227	0.000249	0.000146882	2.376749	0.420743	2.376749
4-Hydroxyphenylacetic acid	0.002588055	0.001971551	0.001865589	0.001118	0.001793	0.000877186	1.696078	0.589596	1.696078
Aspartic acid	0.060034976	0.039548939	0.033116095	0.020866	0.03122	0.018369544	1.883461	0.530938	1.883461
Benzoic acid	0.016587282	0.019089033	0.010556995	0.005356	0.00498	0.006482341	2.749051	0.363762	2.749051
Creatinine	0.060738013	0.040838738	0.10236399	0.070265	0.129085	0.114186169	0.650453	1.537389	-1.537389
Hippuric acid	0.010941015	0.007911999	0.005211707	0.012253	0.013363	0.011861524	0.642104	1.557381	-1.557381
Lactic acid	0.586895388	0.408195755	0.381282383	0.205498	0.290063	0.206054431	1.961722	0.509756	1.961722
Malic acid	0.000878247	0.000679909	0.000573794	0.000513	0.000678	0.000554147	1.221818	0.818452	1.221818
Nicotinic acid	0.016064398	0.011125442	0.011180376	0.00587	0.009505	0.003727012	2.008661	0.497844	2.008661
Ornithine	0.364067773	0.316067217	0.265511658	0.056998	0.092048	0.043341605	4.915328	0.203445	4.915328
Putrescine	0.00192017	0.001275059	0.001563006	0.000394	0.000806	0.000242407	3.300636	0.302972	3.300636
S-Adenosylmethionine	0.01465393	0.010451061	0.008897345	0.004666	0.00528	0.003768098	2.479433	0.403318	2.479433
Succinic acid	0.006525063	0.006835477	0.005088751	0.002312	0.003223	0.002103089	2.415366	0.414016	2.415366
trans-Cinnamic acid	8.51334E-05	9.58137E-05	2.98535E-05	0.000132	0.000113	9.14163E-05	0.627539	1.593526	-1.593526

Name	8h	16h	32h	Method
2-Hydroxybutyric acid	1.011114	1.81454	3.577148	MCF
2-ketoglutamarate	1.189957	1.027557	2.435172	MCF
2-Oxoglutaric acid	1.458799	1.613408	1.142145	MCF
4-Aminobutyric acid (GABA)	2.636552	1.954438	2.376749	MCF
4-Hydroxyphenylacetic acid	1.265864	1.605856	1.696078	MCF
Aspartic acid	1.191004	1.086195	1.883461	MCF
Benzoic acid	1.119071	1.432133	2.749051	MCF
Creatinine	-1.39329	-1.00132	-1.53739	MCF
Hippuric acid	1.010356	-1.68697	-1.55738	MCF
Lactic acid	-1.00329	1.379092	1.961722	MCF
Malic acid	1.018313	-1.0083	1.221818	MCF
Nicotinic acid	1.225243	1.384777	2.008661	MCF
Ornithine	1.33416	2.755266	4.915328	MCF
Putrescine	-1.10266	2.027173	3.300636	MCF
S-Adenosylmethionine	1.114279	1.270251	2.479433	MCF
Succinic acid	2.069569	2.435094	2.415366	MCF
trans-Cinnamic acid	1.255605	-1.40458	-1.59353	MCF
