# Supplementary material for: Metabolic cooperation supports mutual growth between the protozoan parasite Trichomonas vaginalis and dysbiotic vaginal bacteria
Source: PLoS Pathog. 2026 Sep 23;22(9):e1014620. doi: 10.1371/journal.ppat.1014620 (PMC13626468; doi:10.1371/journal.ppat.1014620)
Supplement: Supplementary file 3 [file ppat.1014620.s004.pdf]

Table S2. Ranked metabolites from the TMS dataset showing the greatest fold-differences between mixed and axenic cultures across timepoints. Values shown for individual samples are normalized GC-MS metabolite responses after trimethylsilyl (TMS) derivatization. 'allT' indicates mixed cultures containing *T. vaginalis* and all three BV-associated bacteria; 'TT' indicates axenic *T. vaginalis* cultures. Numbers 8, 16 and 32 indicate incubation timepoints in hours, and letters a-c indicate biological replicates. 'allvstrich' represents the fold-change calculated as mixed culture / axenic *T. vaginalis*. 'trichvsall' represents the reciprocal comparison.

Name	allT8a	allT8b	allT8c	TT8a	TT8b	TT8c
LACTIC ACID	109.4457	71.97292	114.5653	94.84547	114.1085	122.746
1,4-Butanediol, 2TMS derivative	19.1799	10.10968	22.11677	21.39142	27.36124	22.80026
GLYCEROL	2.463533	1.519296	2.069509	2.097345	2.470543	2.476406
1-Phenylethanol, TMS derivative	0.225284	0.158768	0.18721	0.195371	0.11938	0.171299
2-Hydroxybutyric acid, 2TMS deriva	1.247974	0.553825	2.171455	0.899251	1.153488	1.025856
TREHALOSE	0.230146	0.186188	0.358665	0.000001	0.000001	0.000001
MALTOSE oxime2	0	0.048747	0	0.063308	0.075194	0.130575
4-Aminobutanoic acid, 3TMS deriva	0	0.107651	0.131603	0.044929	0.039535	0
4-Aminomethylcyclohexane carbox	0.431118	0.409276	0.164968	0.206263	0.217054	0.202973
Ascorbic acid, 4TMS derivative	1.419773	1.907921	1.173309	1.671205	2.028682	3.455721
Cysteine, 3TMS derivative	0.08752	0.066012	0.159407	0.19469	0.241085	0.243697
D-Glucose, 5TMS derivative	1164.447	839.063	1301.59	1004.482	1310.561	927.3588
DL-Ornithine, 4TMS derivative	1.016207	0.265741	0.570899	0.706603	0.605426	0.588882
L-Alanine, 2TMS derivative	8.594814	4.170616	8.387396	7.856365	10.07907	8.217195
L-Glutamic acid, 3TMS derivative	5.711507	3.076168	5.417053	4.973451	6.989922	5.544279
L-Glutamine, 3TMS derivative	2.380875	0.740691	1.46988	9.371001	5.364341	2.522948
L-Proline, 2TMS derivative	1.831442	0.886594	2.175162	2.854323	2.817054	2.714932
L-Tyrosine, 3TMS derivative	61.09562	32.51726	49.66821	97.41593	115.3488	90.2702
Malic acid	0.495948	0.228504	0.34569	0.349898	0.369767	0.378151
Niacin, TMS derivative	0.259319	0.257617	0.251158	0.228727	0.286822	0.360698
Propylene glycol, 2TMS derivative	0.183144	0.095125	0.267841	0.152485	0.165116	0.157725
Ribose Oxime 1	3.517018	1.820582	3.523633	3.133424	4.000775	3.341306
Serine, 3TMS derivative	28.12318	12.07041	36.35959	47.36828	57.11008	42.13575

allvstrich	trichvsall	FoldDifference8h
0.892324	1.120669	-1.12067
0.718438	1.391908	-1.39191
0.859183	1.163896	-1.1639
1.175315	0.850836	1.175315
1.290606	0.77483	1.290606
258333.2	3.87E-06	258333.2
0.181165	5.519825	-5.51983
2.832635	0.353028	2.832635
1.605264	0.622951	1.605264
0.629018	1.589781	-1.58978
0.460562	2.171261	-2.17126
1.019337	0.98103	1.019337
0.974716	1.02594	-1.02594
0.808822	1.236366	-1.23637
0.811344	1.232523	-1.23252
0.266043	3.758792	-3.75879
0.583475	1.71387	-1.71387
0.47282	2.114968	-2.11497
0.974792	1.02586	-1.02586
0.876573	1.140806	-1.14081
1.148918	0.870384	1.148918
0.8459	1.182172	-1.18217
0.522141	1.915193	-1.91519

Name	allT16a	allT16b	allT16c	TT16a	TT16b	TT16c
LACTIC ACID	236.7487	262.2942	138.8885	228.8889	157.9834	57.53535
1,4-Butanediol, 2TMS derivative	36.87109	46.79547	15.72047	55.85714	33.85738	7.216611
GLYCEROL	8.28842	9.24369	3.404326	4.059524	4.120232	1.39899
1-Phenylethanol, TMS derivative	1.169701	0.857267	0.68178	0.421627	0.286899	0.108305
2-Hydroxybutyric acid, 2TMS deriva	2.605972	2.731941	1.212978	1.68254	1.682421	0.50954
TREHALOSE	0.375091	0.326371	0.180116	0.088294	0.058043	0.047138
MALTOSE oxime2	0.12673	0.113142	0.081115	0.073413	0.115257	0.031425
4-Aminobutanoic acid, 3TMS deriva	0.300073	0.226284	0.096922	0.119048	0.101161	0.030584
4-Aminomethylcyclohexane carbox	1.252731	0.939077	1.206323	0.345238	0.494196	0.354938
Ascorbic acid, 4TMS derivative	3.633649	2.067885	2.143927	4.175595	6.372305	4.186869
Cysteine, 3TMS derivative	0.236708	0.26893	0.15807	0.400794	0.258706	0.089226
D-Glucose, 5TMS derivative	657.3401	870.6005	930.2895	1052.952	1587.37	792.9966
DL-Ornithine, 4TMS derivative	4.193736	6.978242	1.915973	2.468254	2.192371	0.320988
L-Alanine, 2TMS derivative	16.33795	20.13577	6.020799	19.68254	13.65174	3.219416
L-Glutamic acid, 3TMS derivative	8.233795	7.671018	5.014559	7.358135	6.94859	2.170314
L-Glutamine, 3TMS derivative	3.513474	24.40383	3.246672	35.57143	26.8325	0.902918
L-Proline, 2TMS derivative	4.055353	5.294169	1.119384	6.405754	3.431177	0.869529
L-Tyrosine, 3TMS derivative	133.7334	188.7694	64.31947	212.8254	171.0912	40.76319
Malic acid	0.414421	0.430809	0.334027	0.434524	0.512438	0.201459
Niacin, TMS derivative	2.363438	2.858138	1.501664	1.747024	1.302653	0.631874
Propylene glycol, 2TMS derivative	0.539694	0.765883	0.344842	0.313492	0.325041	0.096521
Ribose Oxime 1	4.617626	4.359443	2.678869	4.356151	3.718905	1.389731
Serine, 3TMS derivative	46.59578	74.56919	26.90516	98.43651	76.23881	16.48934

allvstrich	trichvsall	FoldDifference16h
1.435464	0.696639	1.435464
1.025336	0.97529	1.025336
2.185718	0.457516	2.185718
3.316168	0.301553	3.316168
1.69077	0.591446	1.69077
4.556553	0.219464	4.556553
1.458399	0.685683	1.458399
2.485239	0.402376	2.485239
2.84512	0.351479	2.84512
0.532445	1.878127	-1.87813
0.886449	1.128096	-1.1281
0.715992	1.396663	-1.39666
2.627252	0.380626	2.627252
1.162523	0.860198	1.162523
1.269607	0.787645	1.269607
0.492269	2.031411	-2.03141
0.977812	1.022691	-1.02269
0.910856	1.097868	-1.09787
1.026851	0.973851	1.026851
1.826197	0.547586	1.826197
2.245303	0.445374	2.245303
1.231506	0.812014	1.231506
0.774569	1.291041	-1.29104

Name	allT32a	allT32b	allT32c	TT32a	TT32b	TT32c
LACTIC ACID	184.7264	333.7656	343.7631	56.96673	69.19409	80.97756
1,4-Butanediol, 2TMS derivative	16.04941	24.6384	20.64237	6.777583	12.90972	9.129676
GLYCEROL	20.91167	34.71322	40.82916	2.857049	2.73671	6.653367
1-Phenylethanol, TMS derivative	0.407659	1.495636	0.680524	0.084939	0.143127	0.095387
2-Hydroxybutyric acid, 2TMS deriva	1.894379	4.336035	4.745444	0.581655	0.52375	0.920511
TREHALOSE	0.161211	0.282419	0.25	0.037434	0.035231	0.045511
MALTOSE oxime2	0.050031	0.065461	0.10877	0.062391	0.060711	0.055175
4-Aminobutanoic acid, 3TMS deriva	0.081532	0.179551	0.190774	0.037434	0.035546	0.052681
4-Aminomethylcyclohexane carbox	0.591723	3.054863	4.460137	0.336033	0.312048	0.456983
Ascorbic acid, 4TMS derivative	1.429277	2.472569	2.915718	1.823336	1.633218	3.363466
Cysteine, 3TMS derivative	0.151946	0.279925	0.328018	0.092382	0.133375	0.182045
D-Glucose, 5TMS derivative	851.6467	1076.349	1247.636	572.1891	726.6889	768
DL-Ornithine, 4TMS derivative	2.256949	7.596633	6.603645	0.232706	0.408934	0.454489
L-Alanine, 2TMS derivative	8.75664	17.22195	22.34169	3.623468	4.8229	7.129676
L-Glutamic acid, 3TMS derivative	2.575664	10.41397	7.036446	2.300131	3.235923	2.899314
L-Glutamine, 3TMS derivative	2.494132	13.31172	10.09112	0.846979	4.167977	1.03086
L-Proline, 2TMS derivative	0.91538	2.34601	2.288724	0.653897	1.032086	0.947631
L-Tyrosine, 3TMS derivative	20.04694	115.591	116.5923	33.77933	65.36898	39.87032
Malic acid	0.219271	0.387157	0.34795	0.172942	0.24945	0.22288
Niacin, TMS derivative	2.400247	4.61596	4.357631	1.020797	1.048443	1.665524
Propylene glycol, 2TMS derivative	0.668314	1.419576	0.81549	0.332531	0.368669	0.335411
Ribose Oxime 1	2.183447	5.942643	4.044989	1.515981	2.181818	1.909601
Serine, 3TMS derivative	30.82891	48.01496	39.06606	16.09282	31.05379	16.03491

allvstrich	trichvsall	FoldDifference32h
4.162701	0.240229	4.162701
2.128266	0.469866	2.128266
7.875648	0.126974	7.875648
7.98826	0.125184	7.98826
5.417727	0.184579	5.417727
5.869425	0.170374	5.869425
1.257949	0.794945	1.257949
3.595848	0.278099	3.595848
7.335978	0.136314	7.335978
0.99964	1.00036	-1.00036
1.863378	0.53666	1.863378
1.536439	0.650856	1.536439
15.01396	0.066605	15.01396
3.102217	0.32235	3.102217
2.37406	0.421219	2.37406
4.283453	0.233457	4.283453
2.107414	0.474515	2.107414
1.814363	0.551158	1.814363
1.479032	0.676118	1.479032
3.045397	0.328364	3.045397
2.800837	0.357036	2.800837
2.170539	0.460715	2.170539
1.866209	0.535846	1.866209

Name	8h	16h	32h	Method
LACTIC ACID	-1.12067	1.435464	4.162701	TMS
1,4-Butanediol, 2TMS derivative	-1.39191	1.025336	2.128266	TMS
GLYCEROL	-1.1639	2.185718	7.875648	TMS
1-Phenylethanol, TMS derivative	1.175315	3.316168	7.98826	TMS
2-Hydroxybutyric acid, 2TMS derivative	1.290606	1.69077	5.417727	TMS
TREHALOSE	258333.2	4.556553	5.869425	TMS
MALTOSE oxime2	-5.51983	1.458399	1.257949	TMS
4-Aminobutanoic acid, 3TMS derivative	2.832635	2.485239	3.595848	TMS
4-Aminomethylcyclohexane carboxylic acid, N,N-k	1.605264	2.84512	7.335978	TMS
Ascorbic acid, 4TMS derivative	-1.58978	-1.87813	-1.00036	TMS
Cysteine, 3TMS derivative	-2.17126	-1.1281	1.863378	TMS
D-Glucose, 5TMS derivative	1.019337	-1.39666	1.536439	TMS
DL-Ornithine, 4TMS derivative	-1.02594	2.627252	15.01396	TMS
L-Alanine, 2TMS derivative	-1.23637	1.162523	3.102217	TMS
L-Glutamic acid, 3TMS derivative	-1.23252	1.269607	2.37406	TMS
L-Glutamine, 3TMS derivative	-3.75879	-2.03141	4.283453	TMS
L-Proline, 2TMS derivative	-1.71387	-1.02269	2.107414	TMS
L-Tyrosine, 3TMS derivative	-2.11497	-1.09787	1.814363	TMS
Malic acid	-1.02586	1.026851	1.479032	TMS
Niacin, TMS derivative	-1.14081	1.826197	3.045397	TMS
Propylene glycol, 2TMS derivative	1.148918	2.245303	2.800837	TMS
Ribose Oxime 1	-1.18217	1.231506	2.170539	TMS
Serine, 3TMS derivative	-1.91519	-1.29104	1.866209	TMS
