# Supplementary material for: Metabolic cooperation supports mutual growth between the protozoan parasite Trichomonas vaginalis and dysbiotic vaginal bacteria
Source: PLoS Pathog. 2026 Sep 23;22(9):e1014620. doi: 10.1371/journal.ppat.1014620 (PMC13626468; doi:10.1371/journal.ppat.1014620)
Supplement: Supplementary file 4 [file ppat.1014620.s005.pdf]

Gene	TVAG ID	Amplicon (bp)	Forward Primer (5′-3′)	Reverse Primer (5′-3′)
**Tv_ADI1**	TVAG_344520	374	CTACGATAAGAACCAACAGAGAA	TCTCGTGGAAATCGAGATAATG
**Tv_OCT1**	TVAG_041310	255	TCTCGCCATCCAGCATCAAGGCTCAT	GCGCGCTTGTTAAGCGGGGCCATA
**Tv_OCT2**	TVAG_368740	253	CCTTGCTATTGATATCAAGGCCAA	ACGCTTGTGAGACGAGCCAT
**Tv_CK1**	TVAG_261970	380	GACGAACAAGAAGACAGAATC	TTAGATATTGGGATCGACAGGA
**α-tubulin**	TVAG_206890	100	TGCCCAACAGGCTTCAAGAT	TTAGCGAGCATGCAGACAGC
