# Supplementary material for: Metabolic cooperation supports mutual growth between the protozoan parasite Trichomonas vaginalis and dysbiotic vaginal bacteria
Source: PLoS Pathog. 2026 Sep 23;22(9):e1014620. doi: 10.1371/journal.ppat.1014620 (PMC13626468; doi:10.1371/journal.ppat.1014620)
Supplement: Supplementary file 5 [file ppat.1014620.s006.pdf]

Category	Target gene	TVAG ID	Name	Sequence (5′–3′)	Purpose/notes
**gRNA1**	Tv_CK1	TVAG_261970	CK_gRNA1	ATGGTCGAAGATGCTGGCCG	Cas9 target 20 nt protospacer at + strand of the Tv_CK1 ORF, and TGG as the protospacer adjacent motif.
**gRNA2**	Tv_CK1	TVAG_261970	CK_gRNA2	AGGATTGTTGTCGCTCTCGG	Cas9 target 20 nt protospacer at + strand, of the CTv_K1 ORF, and TGG as the protospacer adjacent motif.
**gRNA**	Tv_Fd	TVAG_003900	As in Janssen et al. (2018)*		
**CK homology arm - forward (F) and reverse (R) primers - upstream of the ATG start codon**	Tv_CK1	TVAG_261970	CK_5UTR_F<br>CK_5UTR_R	TCCTTATCGTACTCCATCCCT<br>TCCATCGTCAATTAGAATTCGAGAC	Amplification of the 5′ homology arm (930 bp from the ATG) for Neo expression cassette.
**CK homology arm - forward (F) and reverse (R) primers - downstream of the TAA stop codon**	Tv_CK1	TVAG_261970	CK_3UTR_F<br>CK_5UTR_R	TAAAGTTCATTTAGATATTGGGATCGACAGG<br>TCTTCAAGATCCAGCCACTC	Amplification of the 3′ homology arm (1014 bp from the TAA) for Neo expression cassette.
**Fd homology arms, upstream and downstream of start and stop codons**	Tv_Fd	TVAG_003900	As in Janssen et al. (2018)*		
**Diagnostic forward and reverse primers for CK (Figure 8)**	Tv_CK1	TVAG_261970	CK_GOI1<br>CK_GOI2	CTGGAATCAAGAGATCTCCATGAG<br>AACGTGGAGTGGCATAGCTGG	CK_GOI1 + CK_GOI2: intact CK gene locus, 1,493 bp.<br>CK_GOI1 + Neo2: disrupted CK by Neo insertion, 1,448 bp.
**Diagnostic forward and reverse primers for Fd (Figure 8)**	Tv_Fd	TVAG_003900	Fd_GOI1<br>Fd_GOI2	CGATCGCTTCGCTCGCGGCTT<br>GCGTTAGCTGGCTGATCCTC	Fd_GOI1 + Fd_GOI2: intact Fd gene locus, 1,477 bp.<br>Fd_GOI1 + Neo2: disrupted Fd by Neo insertion, 1481 bp
**Diagnostic forward and reverse primers for Neo (Figure 8)**	Neomycin-resistance gene, no TVAG ID		Neo_1<br>Neo_2	ATTGAACAAGATGGATTGCACGC<br>AACGTCGAGCACAGCTGCGC	Neo1 + Neo2: Neo ORF, 246 bp.

*Janssen BD, Chen Y-P, Molgora BM, Wang SE, Simoes-Barbosa A, Johnson PJ, et al. CRISPR/Cas9-mediated gene modification and gene knock out in the human-infective parasite *Trichomonas vaginalis*. Scientific Reports. 2018;8(1)
