# Supplementary material for: Metabolic cooperation supports mutual growth between the protozoan parasite Trichomonas vaginalis and dysbiotic vaginal bacteria
Source: PLoS Pathog. 2026 Sep 23;22(9):e1014620. doi: 10.1371/journal.ppat.1014620 (PMC13626468; doi:10.1371/journal.ppat.1014620)
Supplement: Supplementary file 6 [file ppat.1014620.s007.pdf]

This file contains the full GC-MS processing and exploratory analysis reports.

PART A. Time-course TMS dataset related to Figures 5 and 6: Processing, normalization, PCA, score plots and boxplots for *T. vaginalis* + BVAB versus *T. vaginalis* alone.

TMS REPORT – DATA PROCESSING AND EXPLORATORY ANALYSIS

Data-mining

The raw GC-MS spectra was data-mined using an in-house R package "MassOmics" for automated peak integration (peak height). Two separate mass spectral libraries were used for compound detection, identification, and integration: 1) An in-house TMS library consisting mostly of sugar and sugar derivatives and 2) a subset NIST 2014 mass spectral library. The reason for using two libraries is because sugar derivatives are difficult to identify without highly specific retention times. As the in-house library has accurate retention time information, identification of sugars using the in-house library was prioritized over the NIST library identification. The NIST library was adopted for compounds that are not present in the in-house library.

Following automated peak integration, each compound was assessed for retention time shift. Where a shift occurred, the retention time window was manually corrected. Any false positive or duplicated identifications were removed from the dataset based on number of identifications (<2 detected in all samples) and library match factors. The peak responses of all metabolites were then normalized by the internal standard Ribitol to account for response variation introduced during sample preparation.

Data preprocessing

Relative standard deviations (RSD) were calculated for each metabolite identified. Identifications with low relative standard deviations (>20%) (i.e. compounds that have little variation in metabolite response between conditions) can be considered "housekeeping" or stationary compounds that are not affected by conditions of interest. The data was then pre-processed through log transformation and scaled by range scaling.

Exploratory analysis: PCA and Boxplots

PCA is an unsupervised method which project new variables (principal components) that best explain the variance in a dataset without referring to group conditions (i.e. Bacteria only, Trichomonas only, Bacteria AND Trichomonas). The resulting data can be summarized into much fewer variables called *scores* which are a weighted average of the original variables to reduce the dimensionality of the data. Pairwise score plots have been provided for each comparison to show the explained variance (up to 5 PCs) and Score-plots to assess grouping of data by conditions tested. Figures provided in the report was taken from processing the dataset through a web-based analytical platform "Metaboanalyst" (http://www.metaboanalyst.ca/MetaboAnalyst/faces/home.xhtml) as well as the statistical function in our in-house R package "MassOmics".

Furthermore, boxplots for each metabolites detected have been provided to facilitate visual exploration of data distributions according to the different conditions under investigation at each timepoints.

This file contains the full GC-MS processing and exploratory analysis reports.

PCA results summary

PCA of the TMS dataset at 8 hours and 32 hours revealed distinct grouping between the conditions under investigation (Trichomonas and bacteria vs Trichomonas only). However, the first principal component which explains the largest variation in the data was not related to the sample condition.

This file contains the full GC-MS processing and exploratory analysis reports.

TMS data Bacteria and Trichomonas vs Trichomonas Only 8h

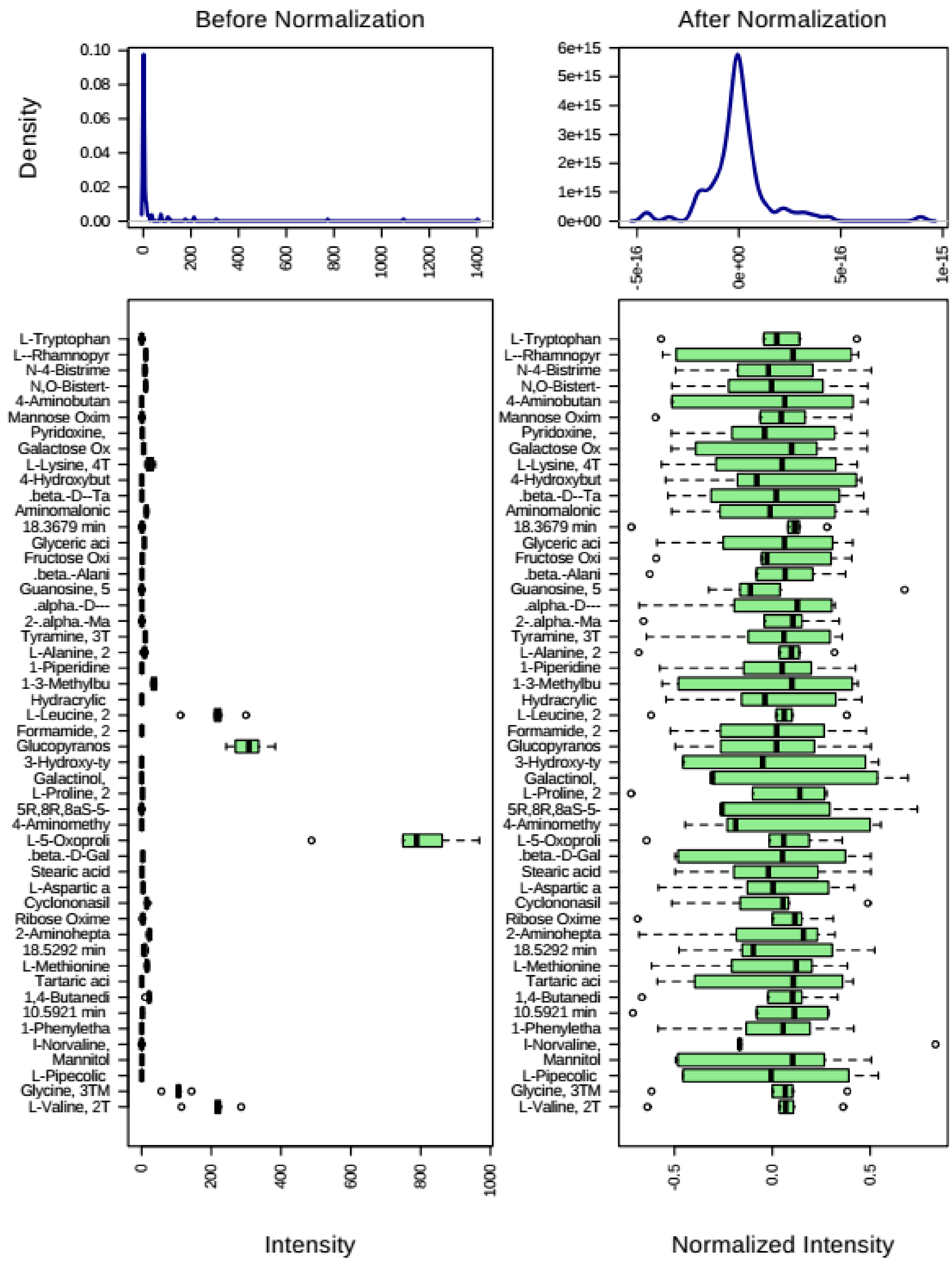

Figure A1. Box plots and density plots before and after data pre-processing of the first 50 compounds identified. Data transformation method: Log transformation, Data scaling method: Range scaling

This file contains the full GC-MS processing and exploratory analysis reports.

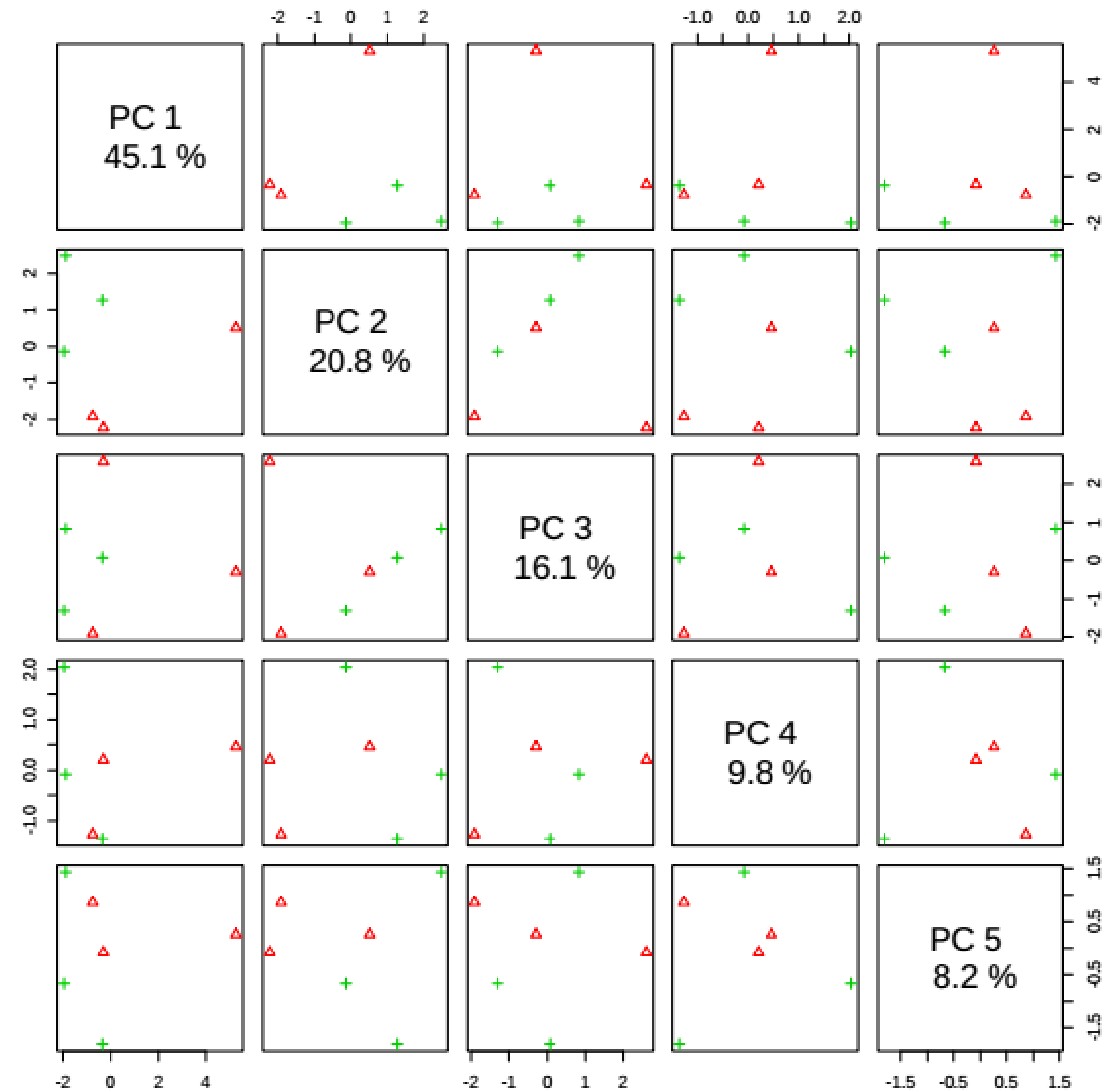

Figure A2. Pairwise score plots between the first 5 components. The diagonal cell displays the variance explained by each principal component.

This file contains the full GC-MS processing and exploratory analysis reports.

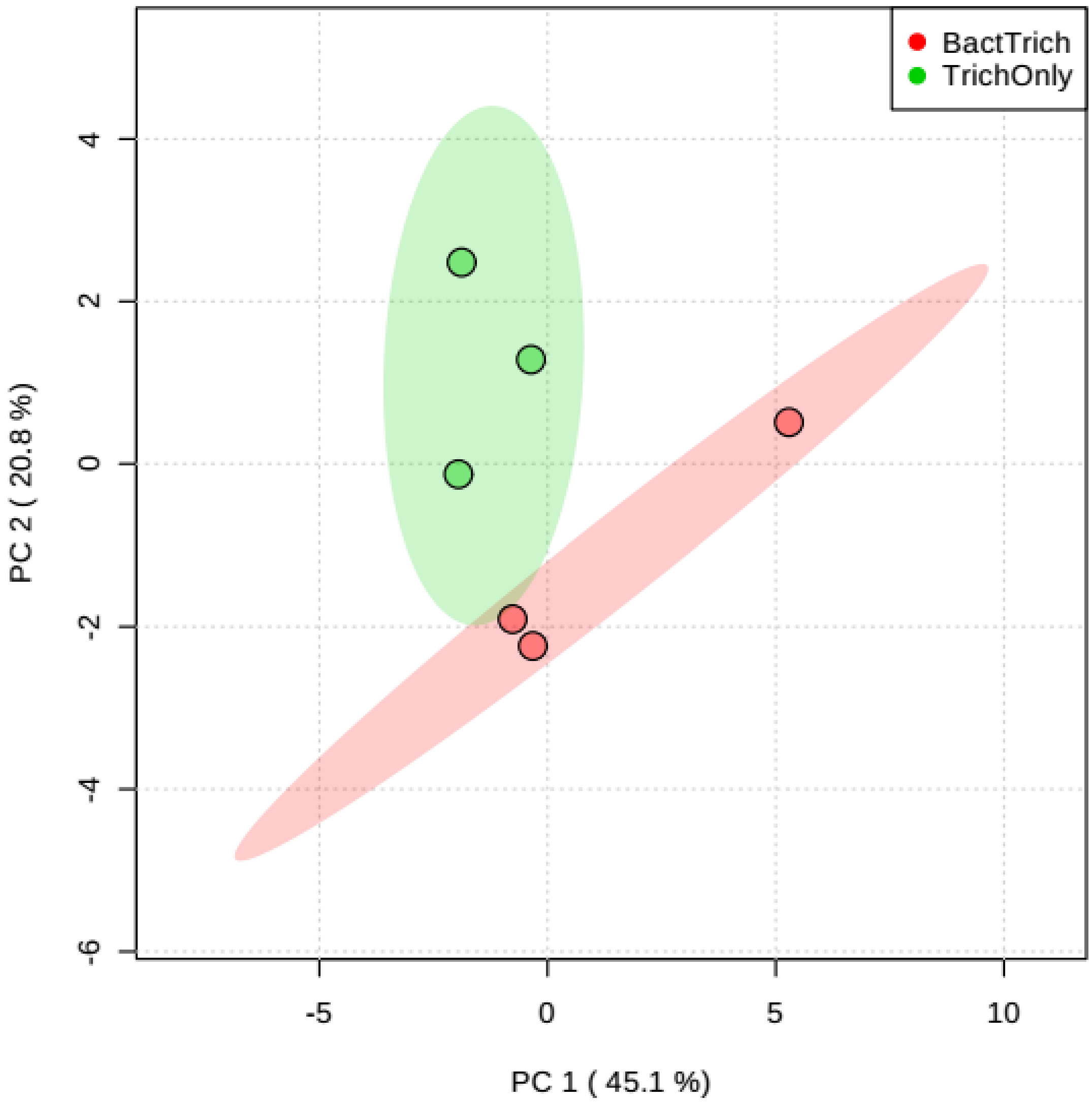

Figure A3. Score plot between the first 2 principal components. Red nodes indicate TMS samples from Bacteria and Trichomonas co-culture; Green nodes indicate TMS samples from Trichomonas only

This file contains the full GC-MS processing and exploratory analysis reports.

TMS data Bacteria and Trichomonas vs Trichomonas Only 16h

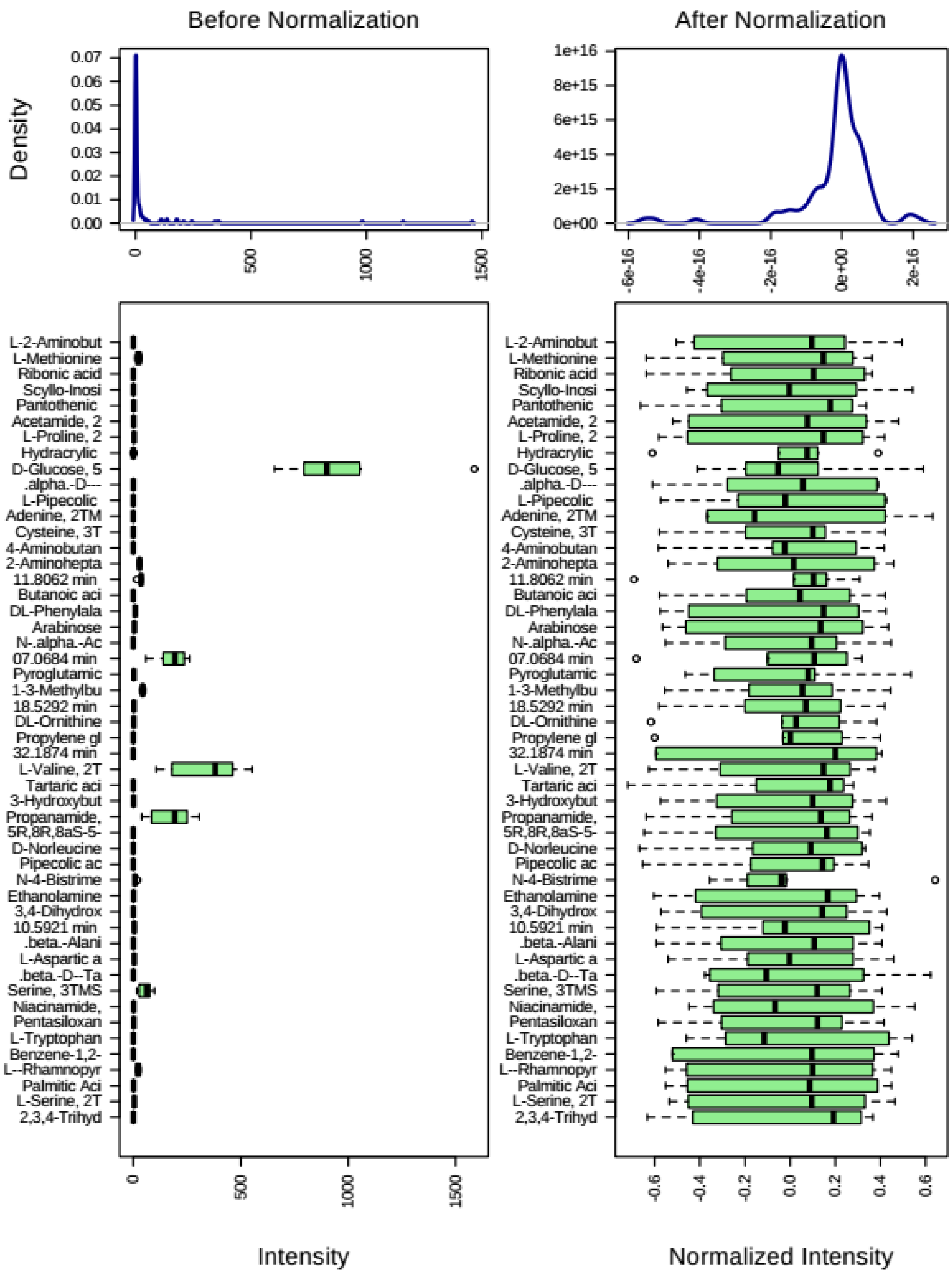

Figure A4. Box plots and density plots before and after data pre-processing of the first 50 compounds identified. Data transformation method: Log transformation, Data scaling method: Range scaling

This file contains the full GC-MS processing and exploratory analysis reports.

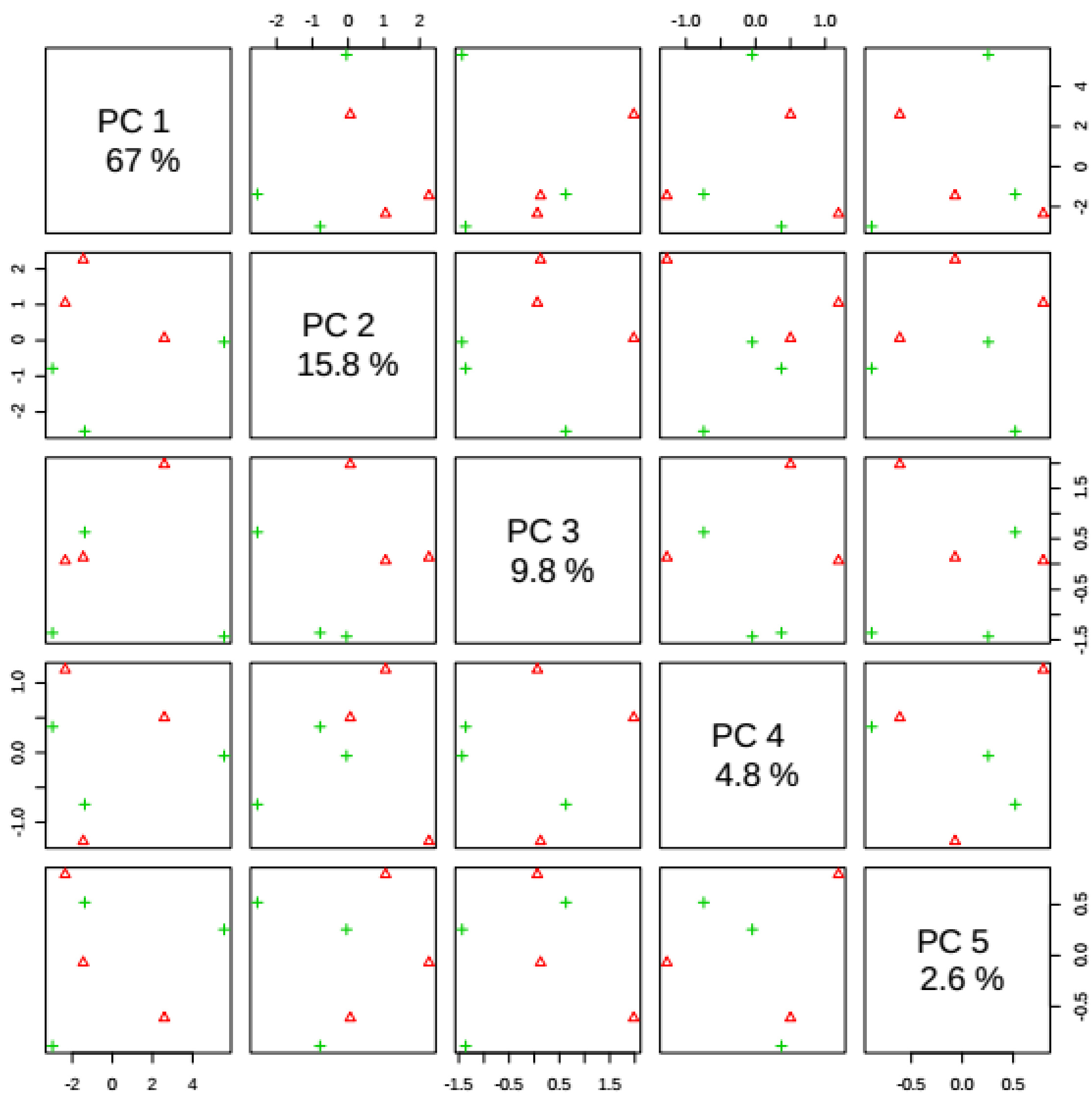

Figure A5. Pairwise score plots between the first 5 components. The diagonal cell displays the variance explained by each principal component.

This file contains the full GC-MS processing and exploratory analysis reports.

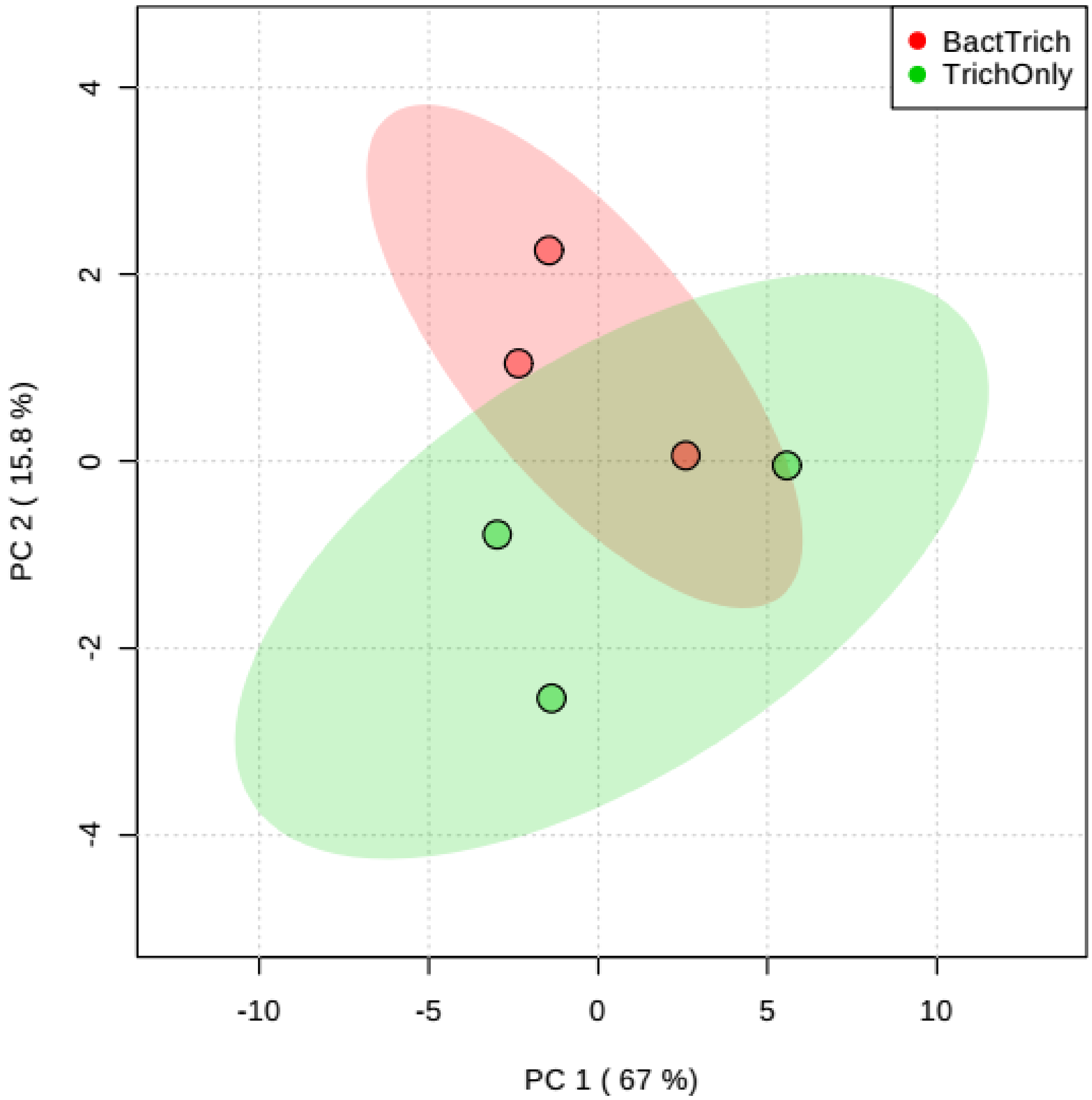

Figure A6. Score plot between the first 2 principal components. Red nodes indicate TMS samples from Bacteria and Trichomonas co-culture; Green nodes indicate TMS samples from Trichomonas only

This file contains the full GC-MS processing and exploratory analysis reports.

TMS data Bacteria and Trichomonas vs Trichomonas Only 32h

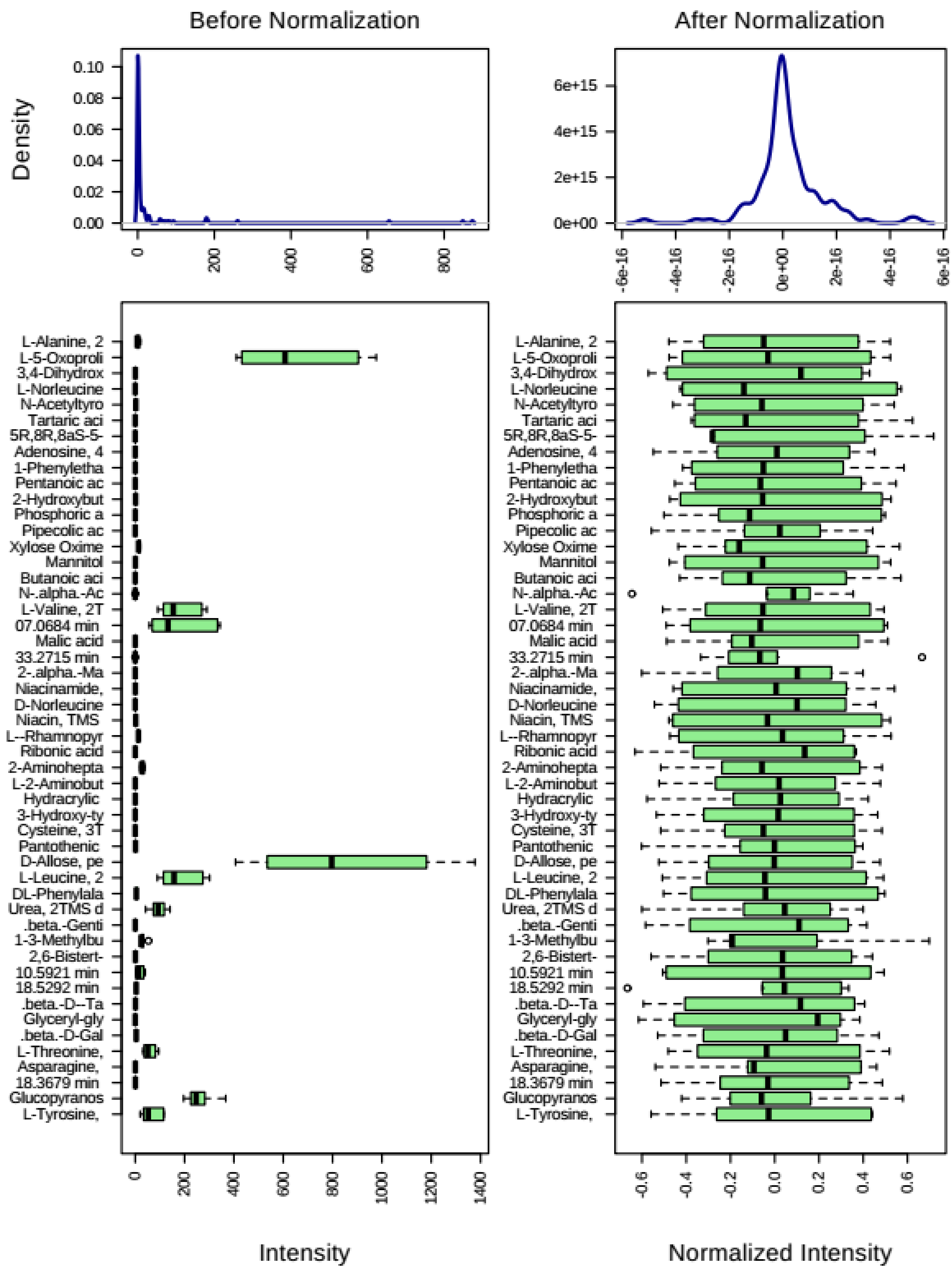

Figure A7. Box plots and density plots before and after data pre-processing of the first 50 compounds identified. Data transformation method: Log transformation, Data scaling method: Range scaling

This file contains the full GC-MS processing and exploratory analysis reports.

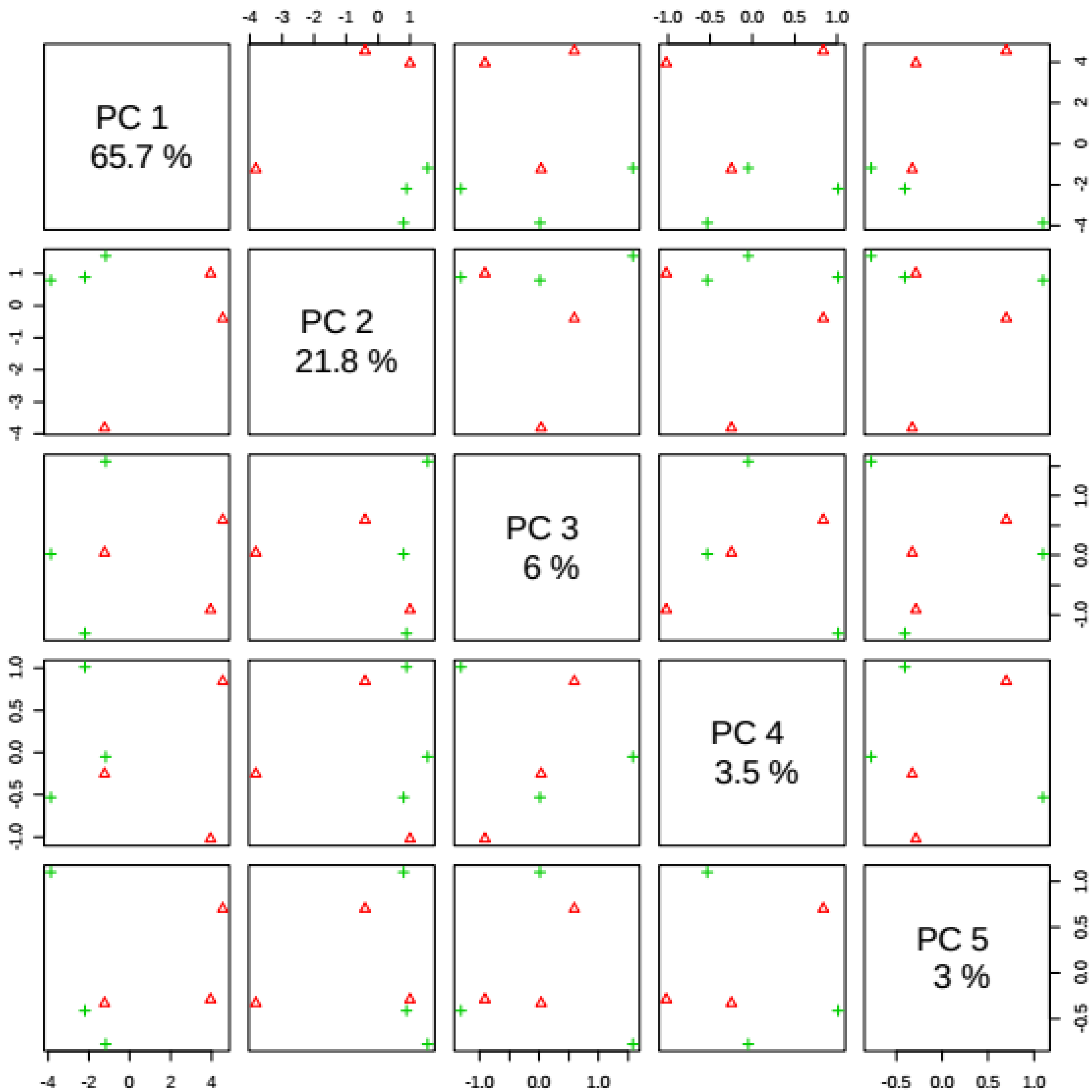

Figure A8. Pairwise score plots between the first 5 components. The diagonal cell displays the variance explained by each principal component.

This file contains the full GC-MS processing and exploratory analysis reports.

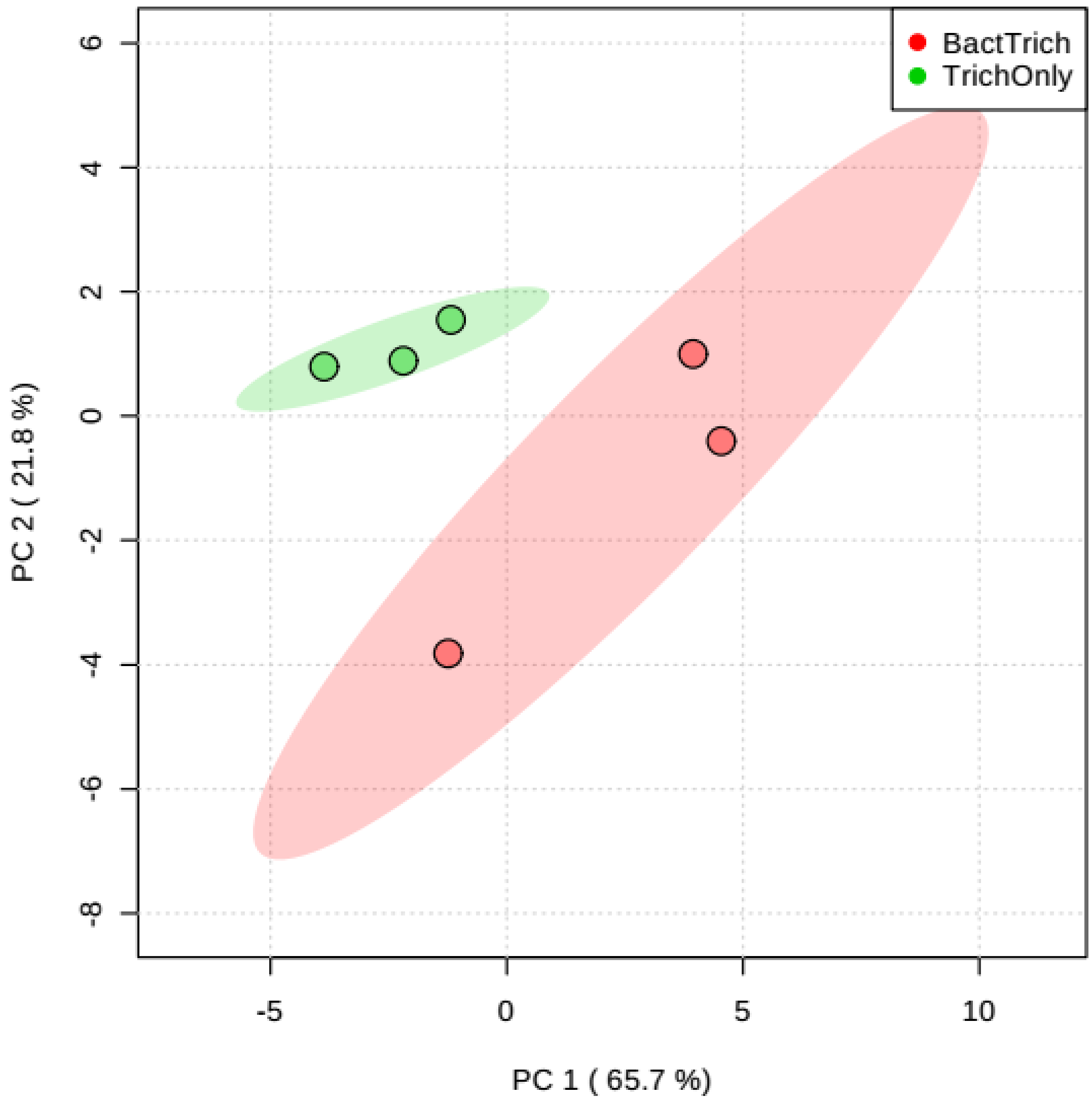

Figure A9. Score plot between the first 2 principal components. Red nodes indicate TMS samples from Bacteria and Trichomonas co-culture; Green nodes indicate TMS samples from Trichomonas only

This file contains the full GC-MS processing and exploratory analysis reports.

PART B. Time-course TMS dataset related to Figures 5 and 6: Processing, normalization, PCA, score plots and boxplots for *T. vaginalis* + BVAB versus *T. vaginalis* alone.

MCF REPORT – DATA PROCESSING AND EXPLORATORY ANALYSIS

Data-mining

The raw GC-MS spectra was data-mined using an in-house R package "MassOmics" for automated peak integration (peak height). An in-house MCF library was used for compound detection and identification. Following automated peak integration, each compound was assessed for retention time shift. Where a shift occurred, the retention time window was manually corrected. Any false positive or duplicated identifications were removed from the dataset based on the number of identifications (<2 detected in all samples) and library match factors. The peak responses of all metabolites were then normalized by the internal standard d4-Alanine to account for response variation introduced during sample preparation.

Data preprocessing

Relative standard deviations (RSD) were calculated for each metabolite identified. Identifications with low relative standard deviations (>20%) (i.e. compounds that have little variation in metabolite response between conditions) can be considered "housekeeping" or stationary compounds that are not affected by conditions of interest (i.e. Bacteria and Trichomonas vs Trichomonas only). In addition, the data was log transformed and scaled using Range scaling.

Exploratory analysis: PCA and Boxplots

PCA is an unsupervised method which project new variables (principal components) that best explain the variance in a dataset without referring to group conditions (i.e. Bacteria only, Trichomonas only, Bacteria AND Trichomonas). The resulting data can be summarized into much fewer variables called *scores* which are a weighted average of the original variables to reduce the dimensionality of the data. Pairwise score plots have been provided for each comparison to show the explained variance (up to 5 PCs) and Score-plots to assess grouping of data by conditions tested. Figures provided in the report was taken from processing the dataset through a web-based analytical platform "Metaboanalyst" (http://www.metaboanalyst.ca/MetaboAnalyst/faces/home.xhtml) as well as the statistical function in our in-house R package "MassOmics". Unknown compounds detected were not included in the exploratory analysis due to the possibility of increasing noise in the data.

Furthermore, boxplots for each metabolites detected have been provided to facilitate visual exploration of data distributions according to the different conditions under investigation.

Comparison between the 2 conditions of interest were performed according to each time-point (i.e. 8h, 16h, 32h).

This file contains the full GC-MS processing and exploratory analysis reports.

Bacteria and Trichomonas vs Trichomonas only: 8h

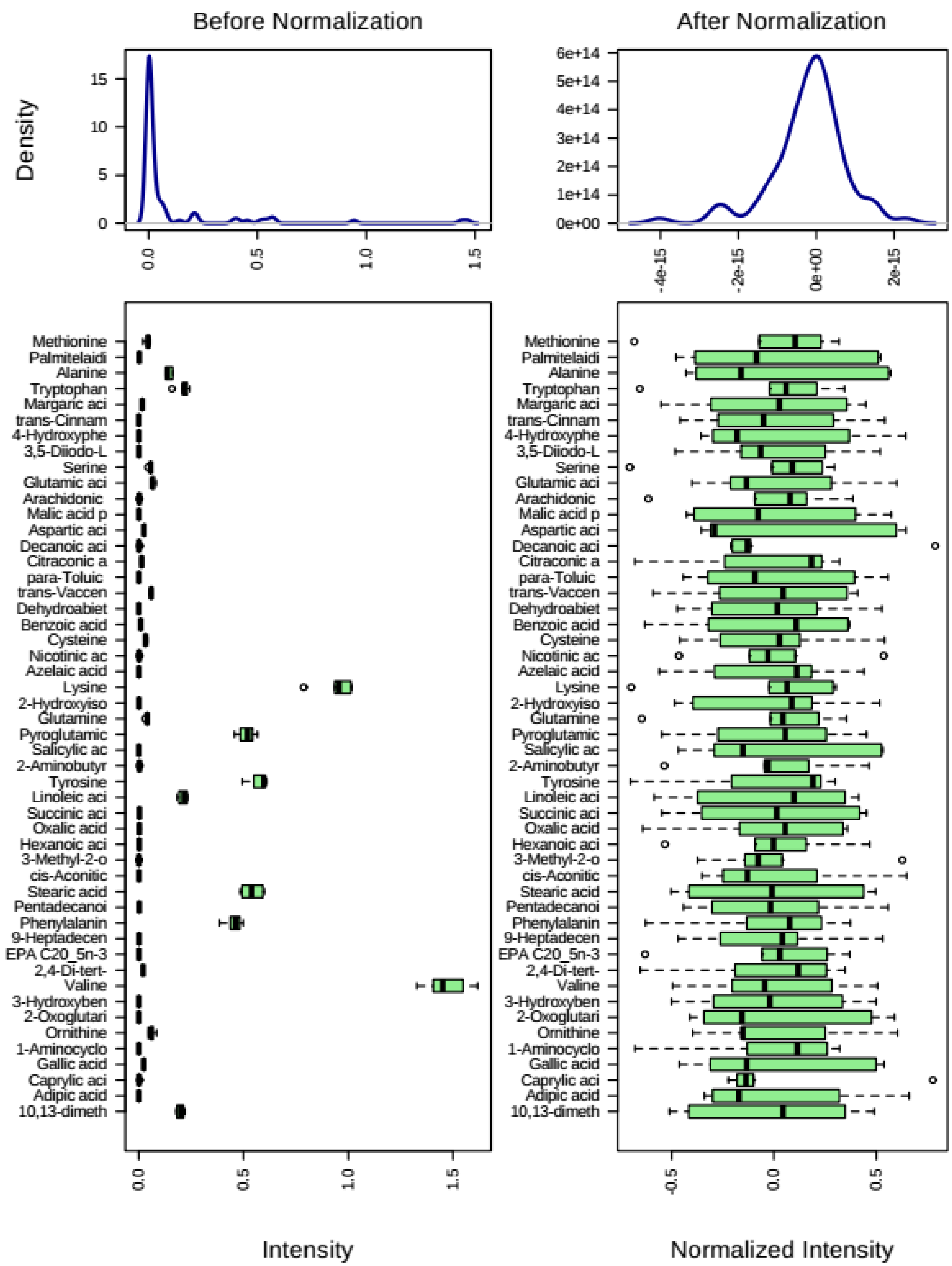

Figure B1. Box plots and density plots before and after data pre-processing of the first 50 compounds identified. Data transformation method: Log transformation, Data scaling method: Range scaling

This file contains the full GC-MS processing and exploratory analysis reports.

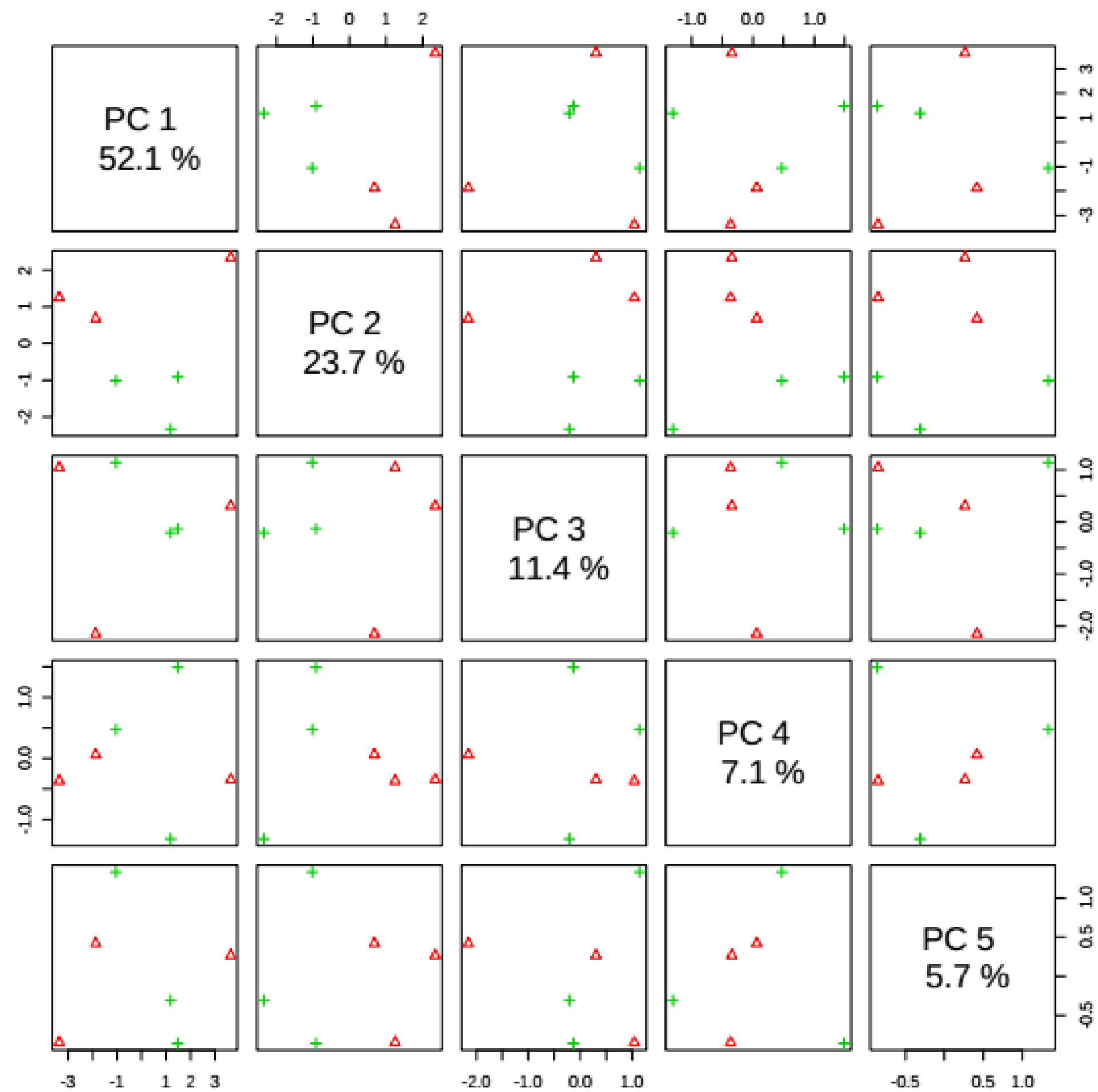

Figure . B2Pairwise score plots between the first 5 components. The diagonal cell displays the variance explained by each principal component.

This file contains the full GC-MS processing and exploratory analysis reports.

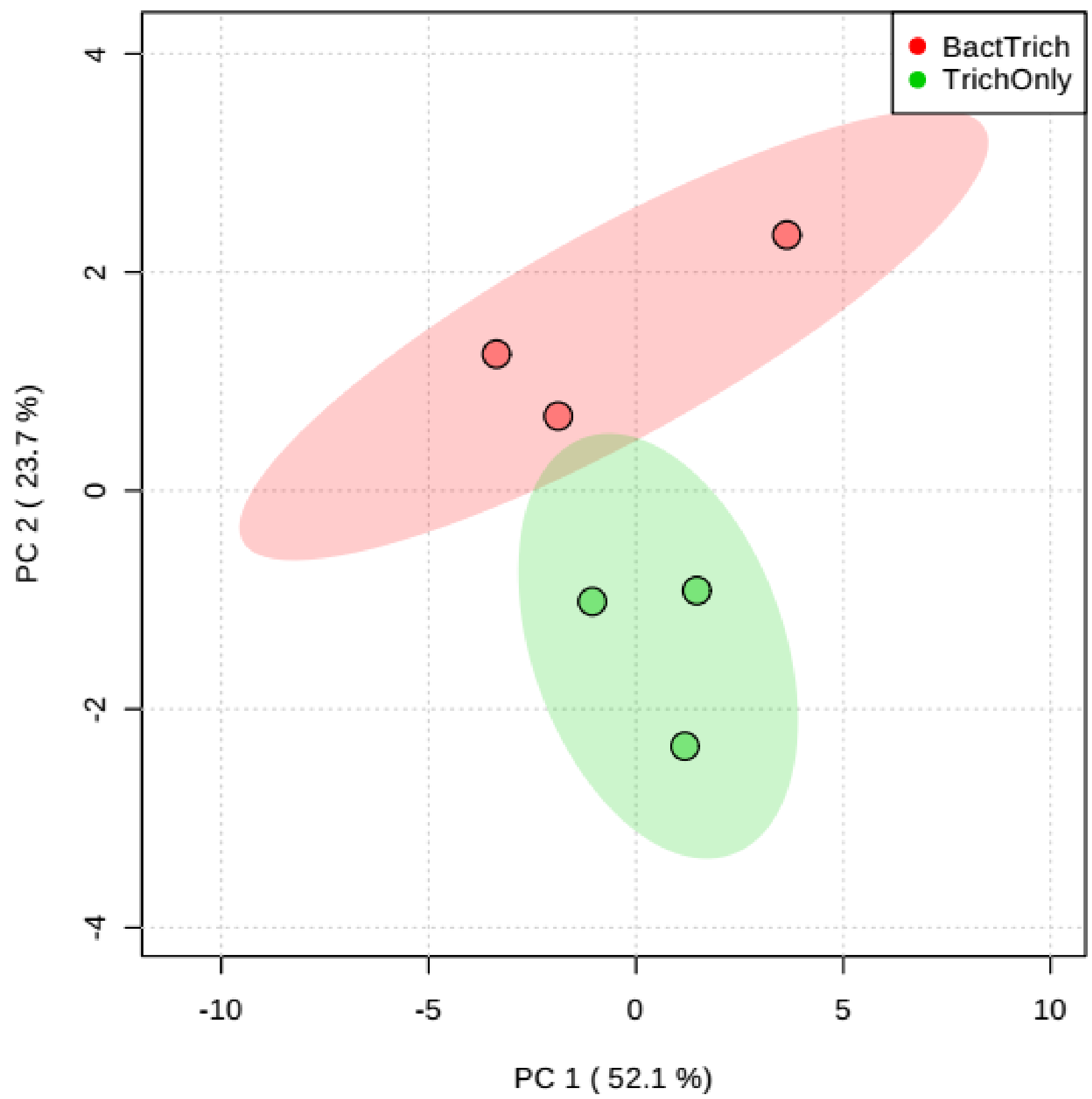

Figure B3. Score plot between the first 2 principal components. Red nodes indicate MCF samples from Bacteria and Trichomonas; Green nodes indicate MCF samples from Trichomonas only. It is evident from the scores plot that the main difference (PC1) is not related to the conditions under interest.

This file contains the full GC-MS processing and exploratory analysis reports.

Bacteria and Trichomonas vs Trichomonas only: 16h

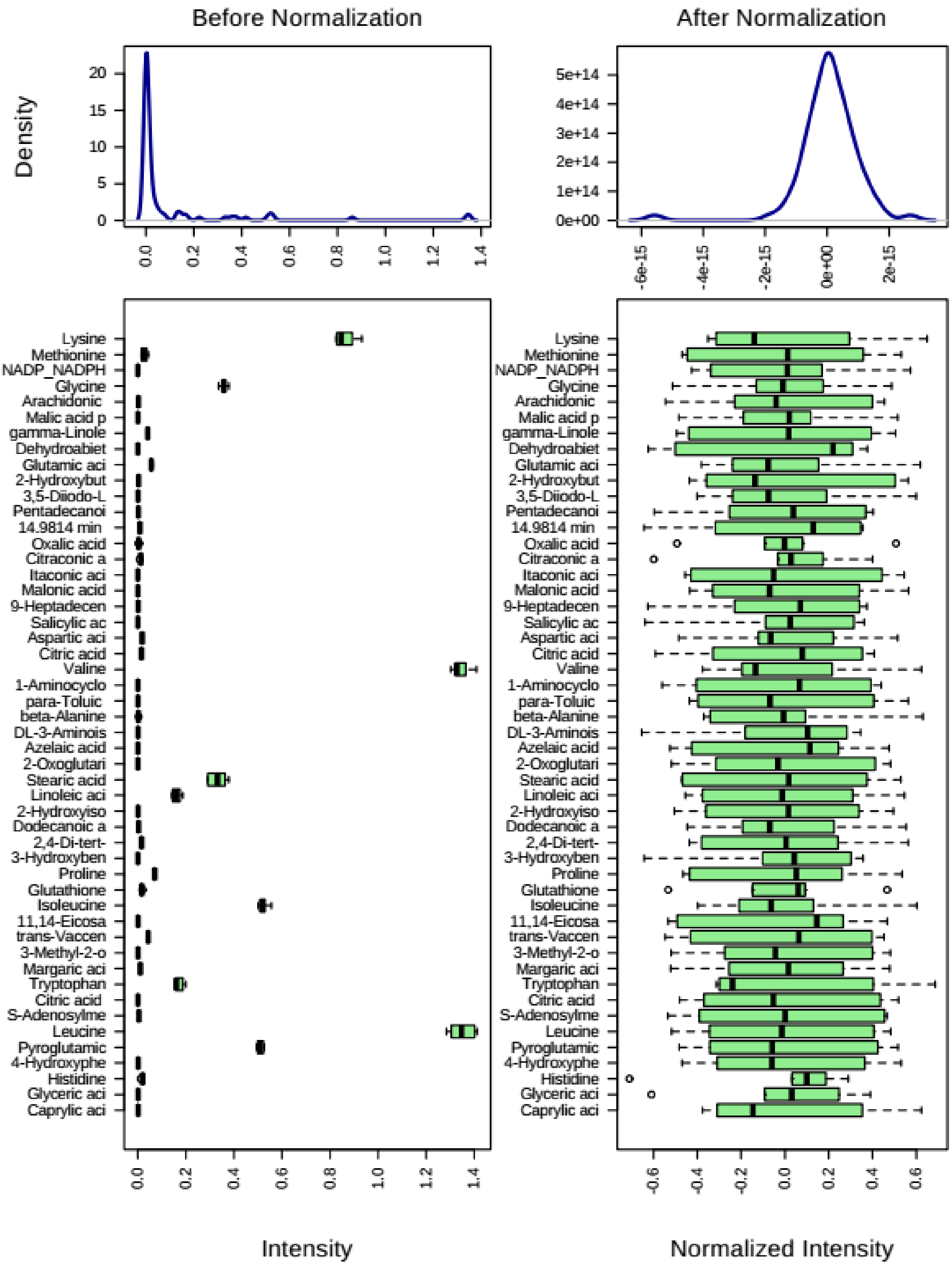

Figure B4. Box plots and density plots before and after data pre-processing of the first 50 compounds identified. Data transformation method: Log transformation, Data scaling method: Range scaling

This file contains the full GC-MS processing and exploratory analysis reports.

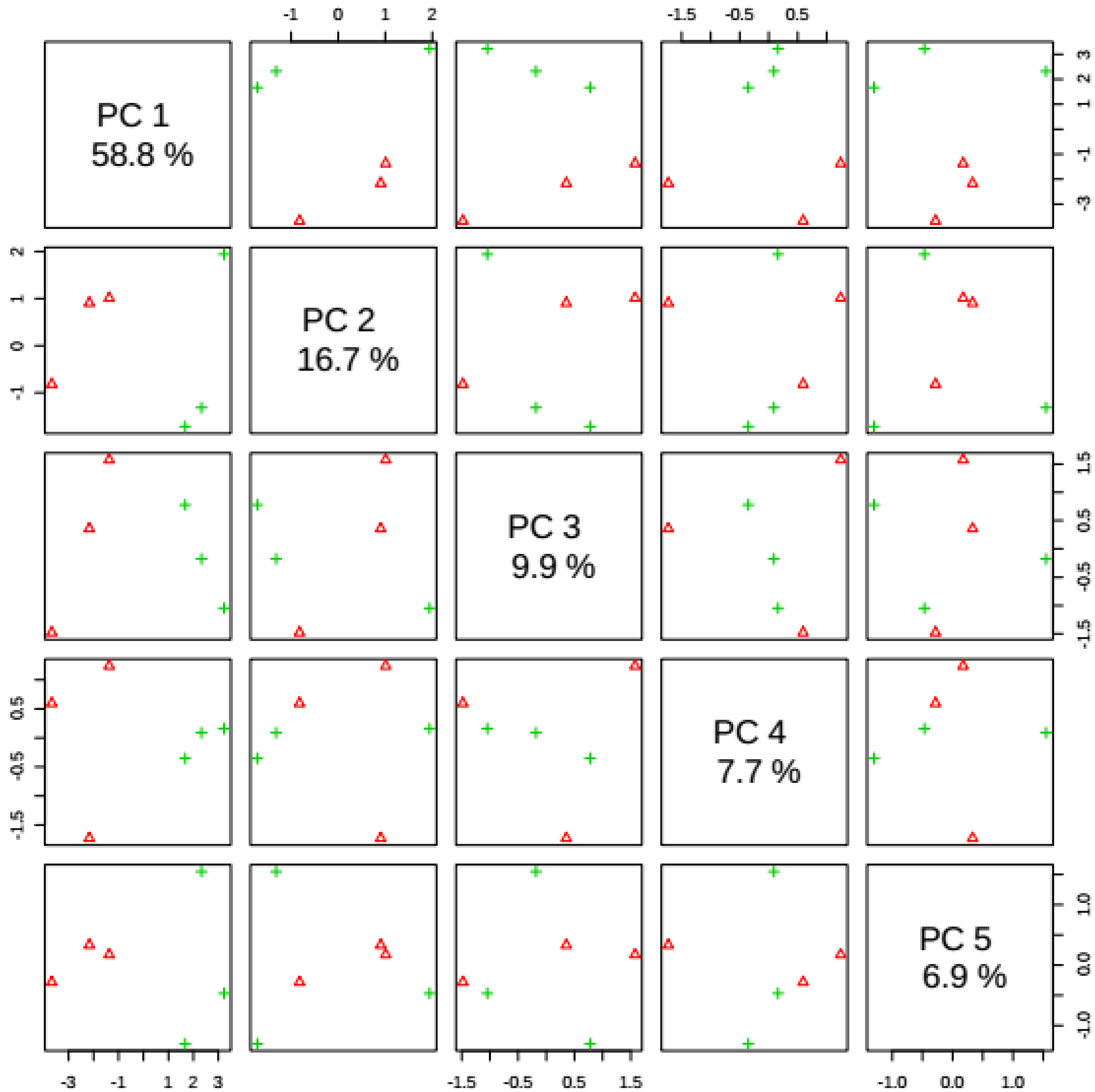

Figure B5. Pairwise score plots between the first 5 components. The diagonal cell displays the variance explained by each principal component.

This file contains the full GC-MS processing and exploratory analysis reports.

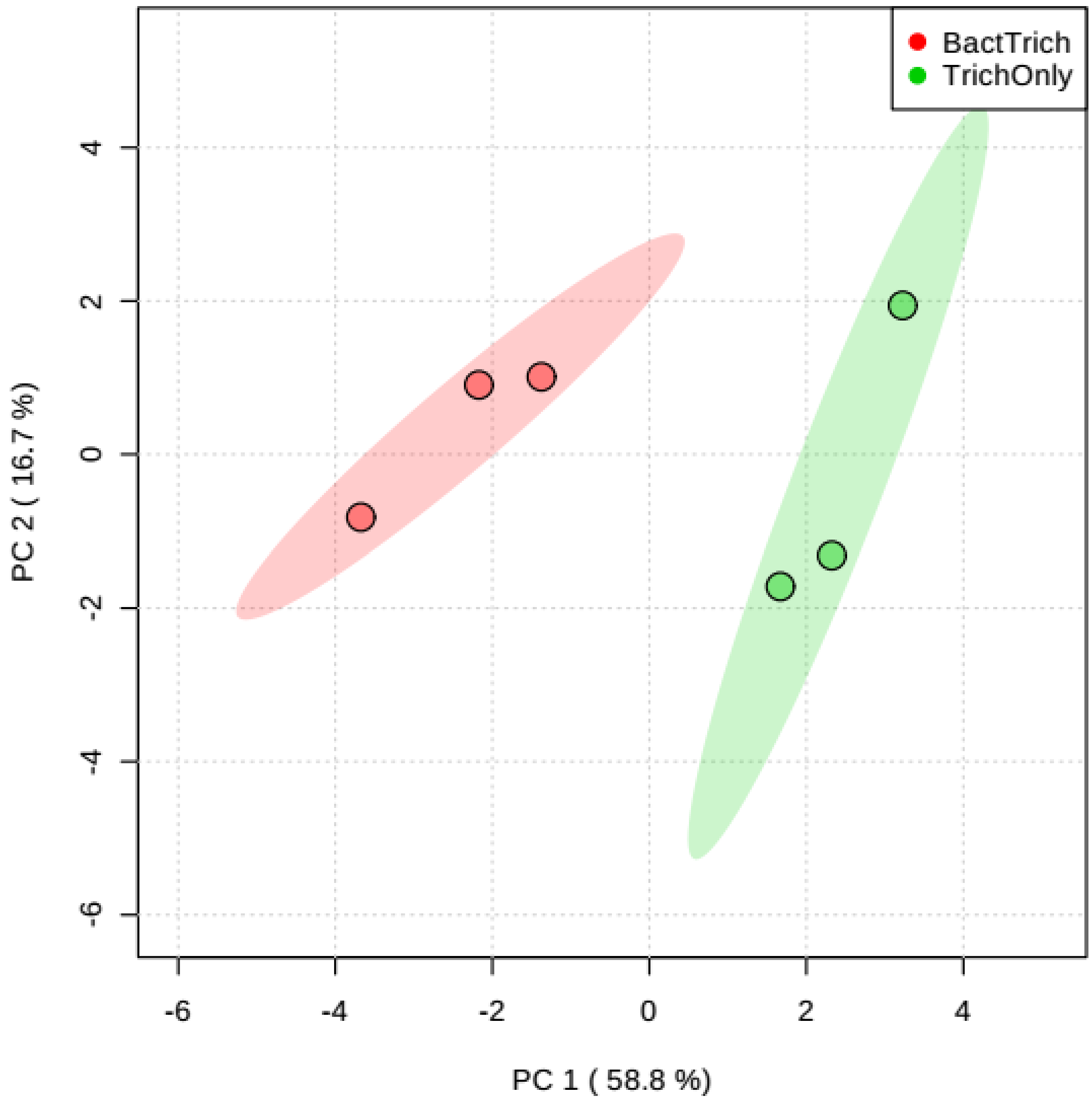

Figure B6. Score plot between the first 2 principal components. Red nodes indicate MCF samples from Bacteria and Trichomonas; Green nodes indicate MCF samples from Trichomonas only. It is evident from the scores plot that the main difference (PC1) in metabolite profile is related to the conditions under interest.

Bacteria and Trichomonas vs Trichomonas only: 32h

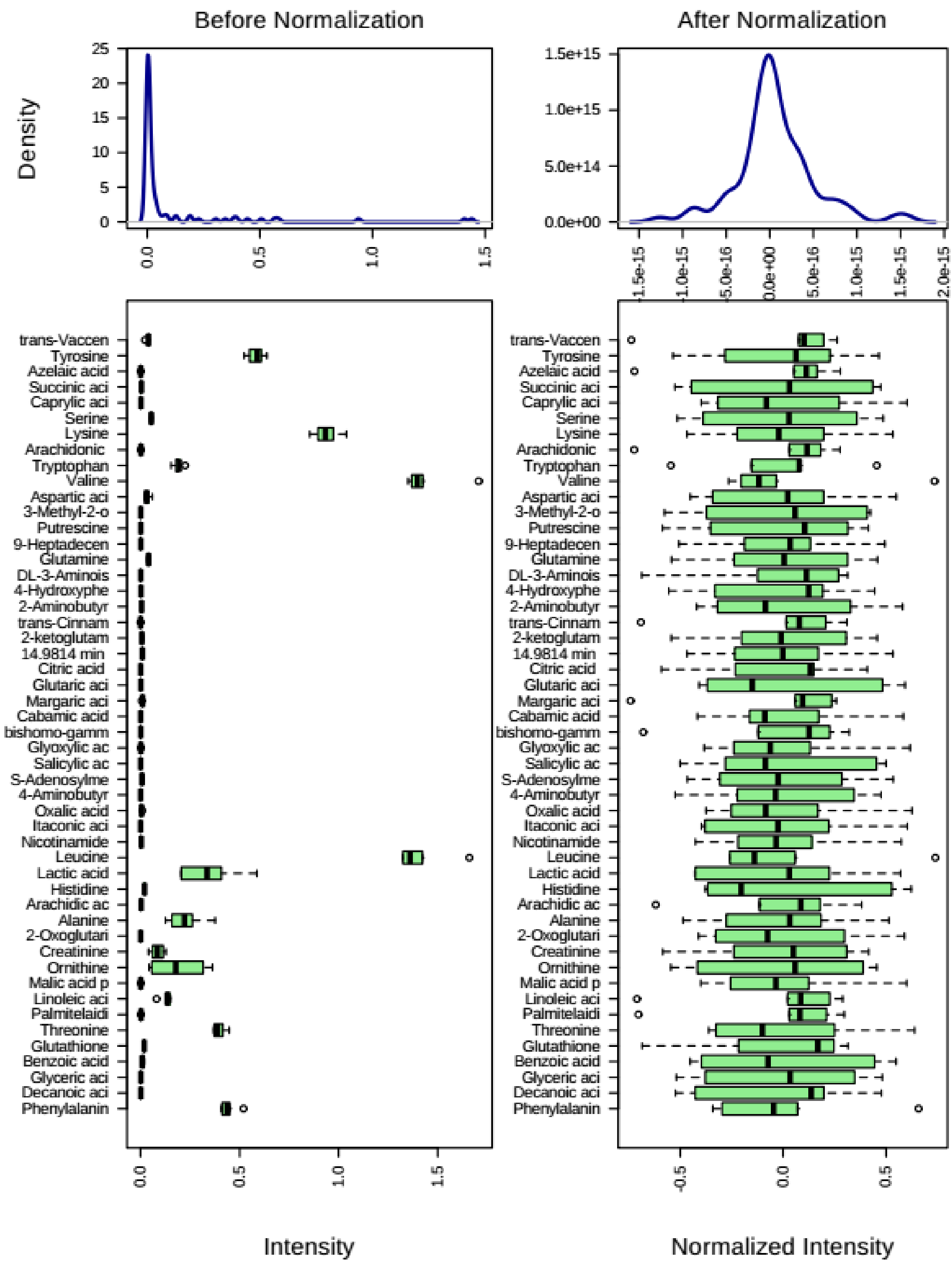

Figure B7. Box plots and density plots before and after data pre-processing of the first 50 compounds identified. Data transformation method: Log transformation, Data scaling method: Range scaling

This file contains the full GC-MS processing and exploratory analysis reports.

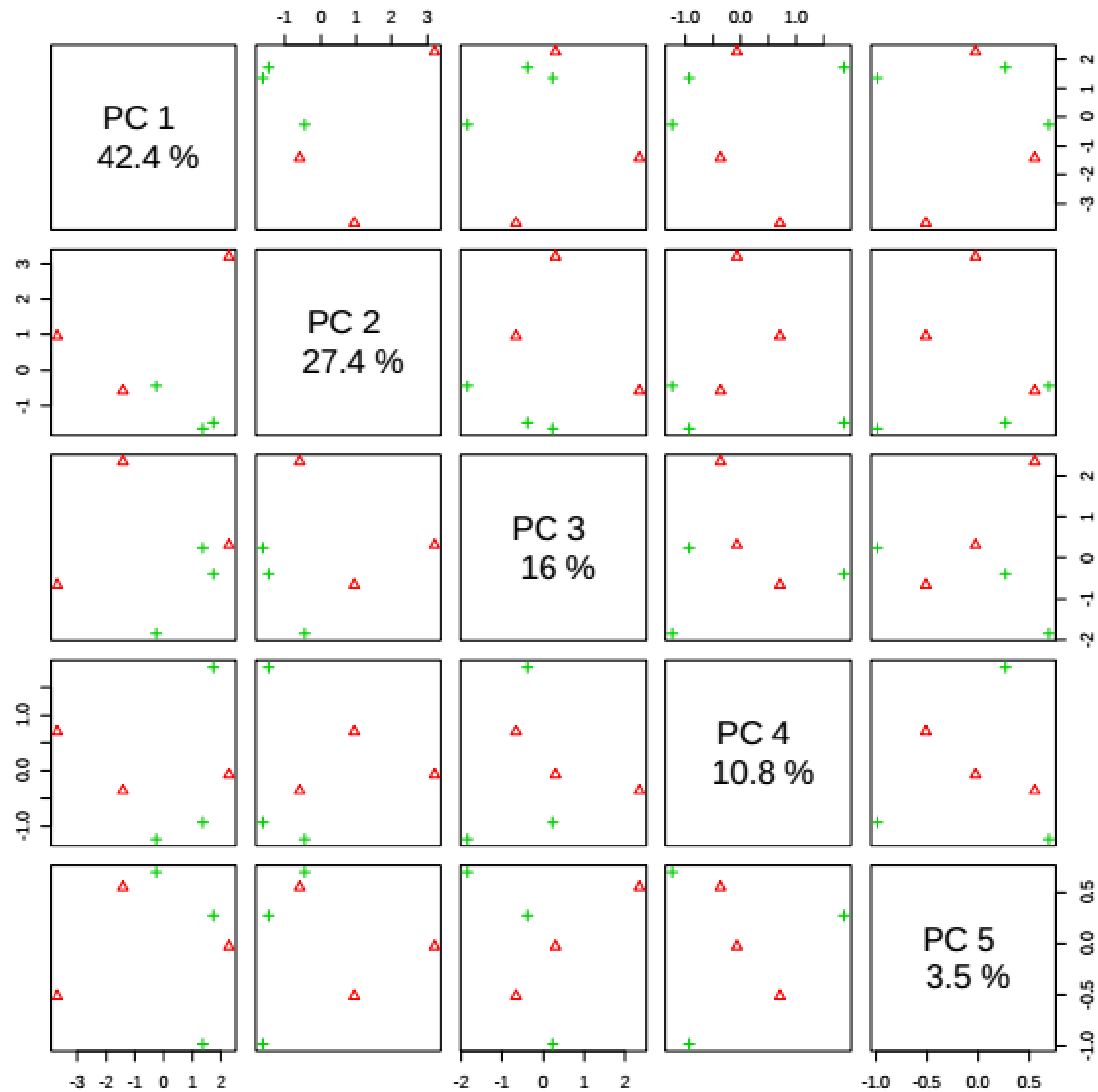

Figure B8. Pairwise score plots between the first 5 components. The diagonal cell displays the variance explained by each principal component.

This file contains the full GC-MS processing and exploratory analysis reports.

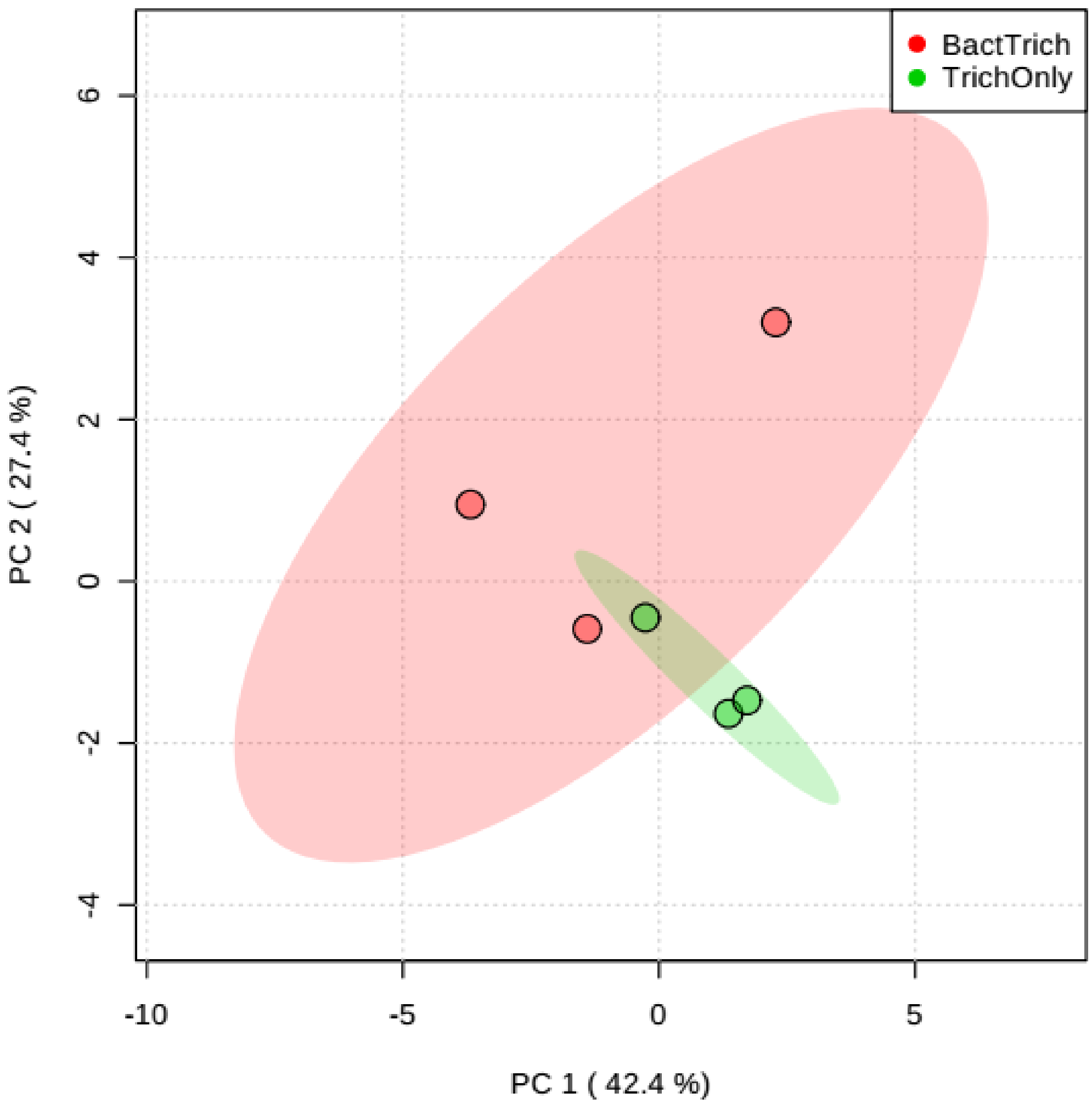

Figure B9. Score plot between the first 2 principal components. Red nodes indicate MCF samples from Bacteria and Trichomonas; Green nodes indicate MCF samples from Trichomonas only. Similar to the 8h timepoint, it is evident from the scores plot that the main difference (PC1) is not related to the conditions under interest.

This file contains the full GC-MS processing and exploratory analysis reports.

PART C. DFMO-treatment MCF dataset related to Figure 8: Processing, normalization and fold-change analysis of DFMO-treated versus untreated cultures in *T. vaginalis* alone and *T. vaginalis*-*G. vaginalis* co-culture.

MCF REPORT – DATA PROCESSING AND EXPLORATORY ANALYSIS OF THE EFFECT OF DMFO

Data-mining

The raw GC-MS spectra was data-mined using an in-house R package "MassOmics" for automated peak integration (peak height). An in-house MCF library was used for compound detection and identification. Following automated peak integration, each compound was assessed for retention time shift. Where a shift occurred, the retention time window was manually corrected. Any false positives or duplicated identifications were removed from the dataset based on the number of identifications (<2 detected in all samples) and library match factors. The peak responses of all metabolites were then normalized by the internal standard d4-Alanine to account for response variation introduced during sample preparation.

Data preprocessing

Further data processing was continued in MetaboAnalyst 6.0 (http://www.metaboanalyst.ca/MetaboAnalyst/faces/home.xhtml). Relative standard deviations (RSD) were calculated for each metabolite identified. Identifications with low relative standard deviations (>20%) (i.e., compounds that have little variation in metabolite response between conditions) can be considered "housekeeping" or stationary compounds that are not affected by conditions of interest (i.e., *Gardnerella vaginalis* and Trichomonas vs Trichomonas only, with or without DFMO). In addition, the data was log transformed and scaled using Range scaling.

Exploratory analysis: Fold changes

Figures provided in the report were taken from processing the dataset MetaboAnalyst 6.0. Unknown compounds detected were not included in the exploratory analysis due to the possibility of increasing noise in the data. Fold-change analysis was performed in MetaboAnalyst 6.0 using the classical feature, and the results were manually plotted in Prism. The two metabolites that were analyzed were Putrescine and Ornithine. Fold-change comparison between the 2 conditions of interest was performed according to the addition of DFMO (i.e., with DFMO or without).

This file contains the full GC-MS processing and exploratory analysis reports.

DFMO vs No DFMO: Trichomonas with *G. vaginalis*

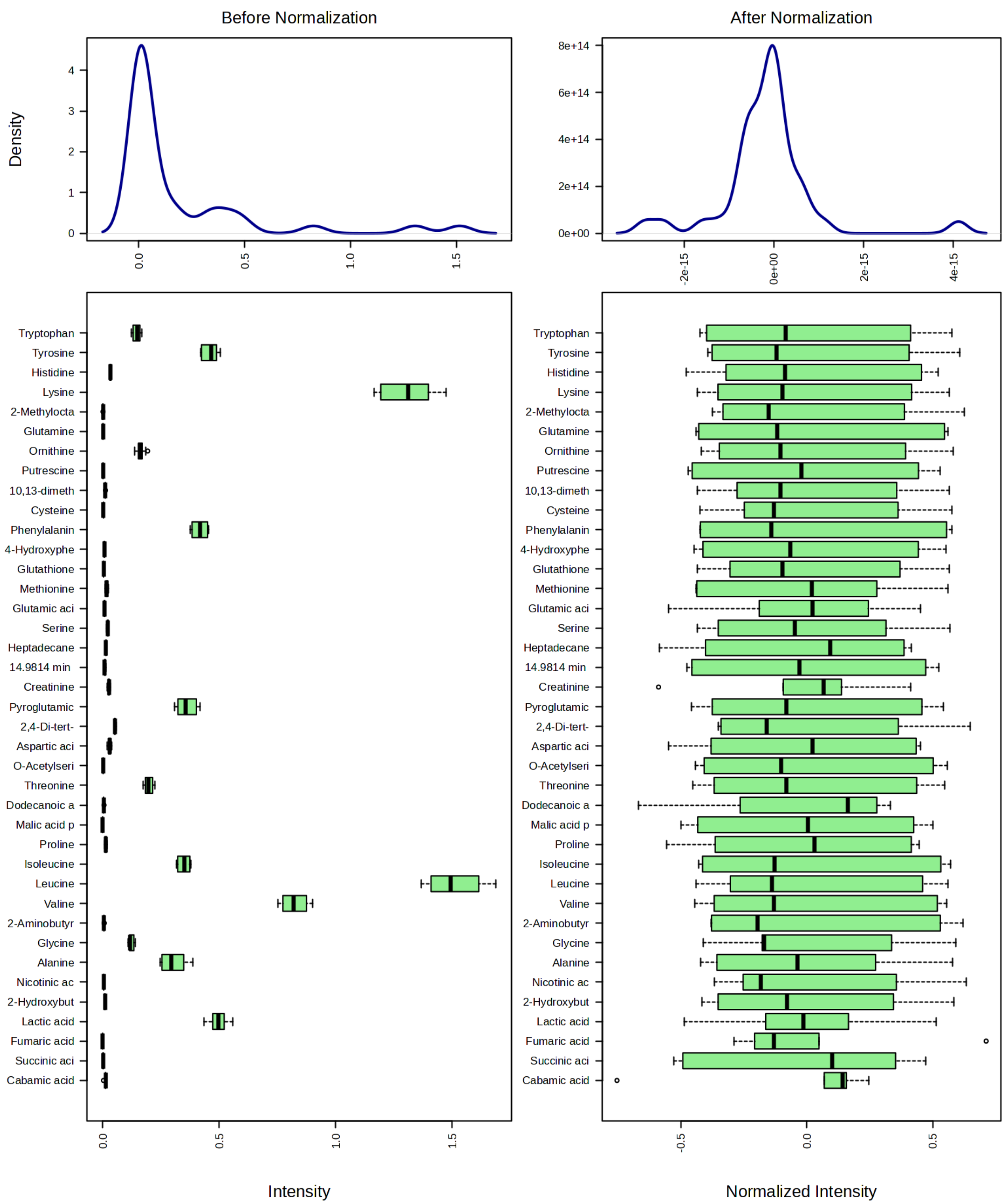

Figure C1. Box plots and density plots before and after data pre-processing of the first 50 compounds identified. Data transformation method: Log transformation, Data scaling method: Range scaling

This file contains the full GC-MS processing and exploratory analysis reports.

DFMO vs No DFMO: Trichomonas alone

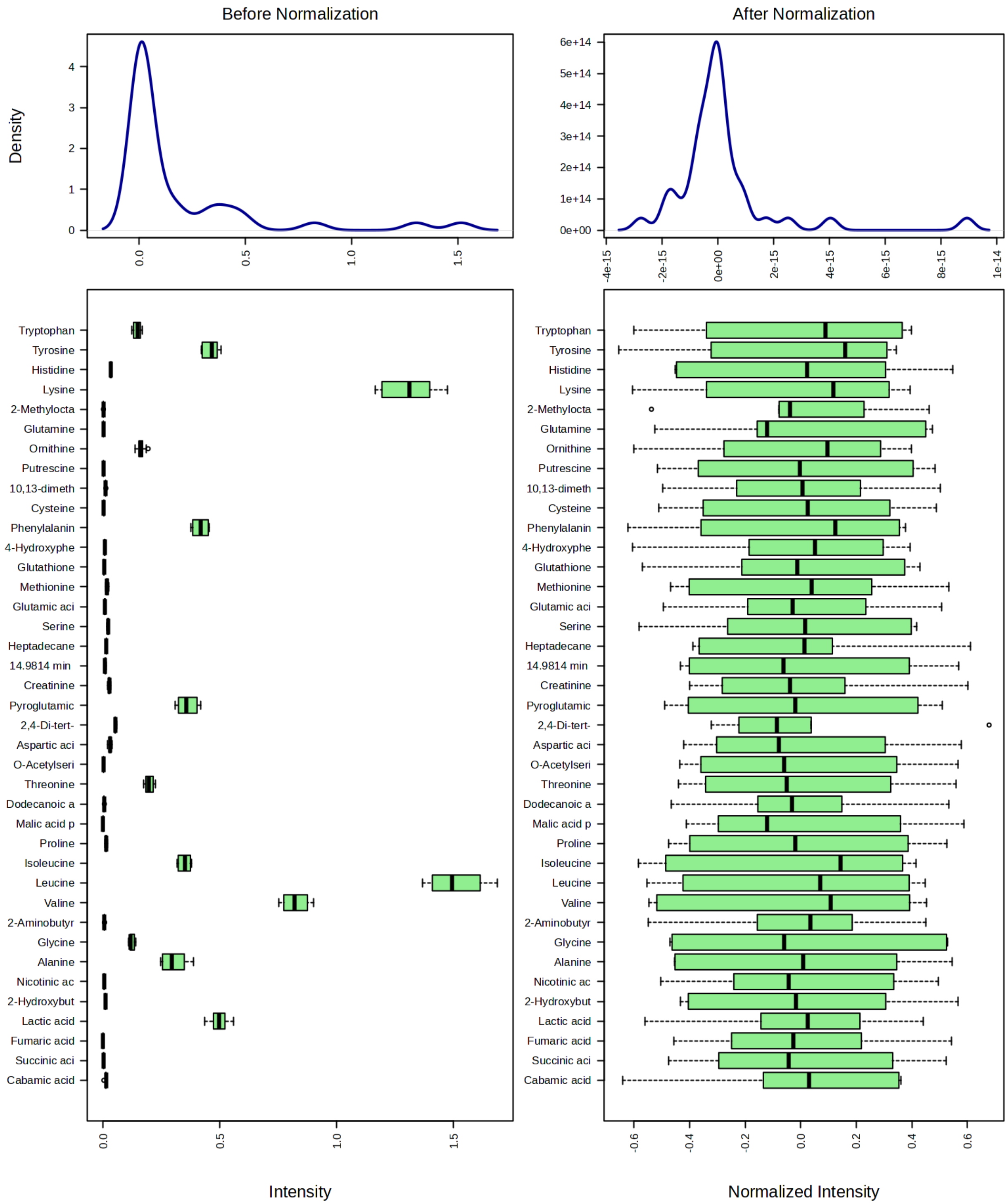

Figure C2. Box plots and density plots before and after data pre-processing of the first 50 compounds identified. Data transformation method: Log transformation, Data scaling method: Range scaling
