# Supplementary material for: Metabolic cooperation supports mutual growth between the protozoan parasite Trichomonas vaginalis and dysbiotic vaginal bacteria
Source: PLoS Pathog. 2026 Sep 23;22(9):e1014620. doi: 10.1371/journal.ppat.1014620 (PMC13626468; doi:10.1371/journal.ppat.1014620)
Supplement: Supplementary file 7 [file ppat.1014620.s008.pdf]

Name	All-16a	All-16b	All-16c	All-32a	All-32b	All-32c	All-8a	All-8b	All-8c	Tv-16a	Tv-16b	Tv-16c	Tv-32a	Tv-32b	Tv-32c	Tv-8a	Tv-8b	Tv-8c
10,13-dimethyltetradecan	0.127595	0.122895	0.123783	0.139166	0.124086	0.078109	0.219939	0.213511	0.178275	0.145565	0.149929	0.154405	0.131569	0.139585	0.137786	0.209571	0.191448	0.181831
11,14-Eicosadienoic (C20_	0.000976	0.000769	0.000782	0.000987	0.000843	0.000456	0.001491	0.001358	0.001085	0.001031	0.001051	0.001138	0.000677	0.000813	0.000657	0.00142	0.001231	0.001063
14.9814 min Dimethyl ami	0.00745	0.009762	0.008441	0.008751	0.007292	0.010432	0.008514	0.008138	0.007044	0.009803	0.006525	0.009463	0.008306	0.012784	0.010243	0.009127	0.009608	0.009469
1-Aminocyclopropane-1-c	0.000318	0.000285	0.000264	0.000456	0.000337	0.000351	0.00038	0.000326	0.000262	0.000402	0.000428	0.000418	0.00043	0.00053	0.000358	0.000377	0.000342	0.000389
2,4-Di-tert-butylphenol (d	0.014266	0.015953	0.014429	0.018433	0.014995	0.015601	0.020042	0.020425	0.018675	0.015255	0.017463	0.016367	0.01346	0.015561	0.015153	0.019974	0.02026	0.019466
2-Aminobutyric acid (From	0.003734	0.003941	0.004	0.007094	0.004349	0.003708	0.004221	0.004544	0.003537	0.004363	0.004969	0.004402	0.005642	0.008839	0.004049	0.004024	0.003997	0.004009
2-Hydroxybutyric acid	0.003446	0.004802	0.005028	0.018617	0.01124	0.009577	0.001249	0.001752	0.001371	0.002332	0.002478	0.002506	0.002956	0.006076	0.001992	0.001317	0.001421	0.001585
2-Hydroxyisobutyric acid	0.000854	0.000706	0.000758	0.000978	0.000834	0.00057	0.001413	0.001314	0.001135	0.001149	0.00097	0.001064	0.000809	0.000942	0.000873	0.001265	0.001157	0.001308
2-ketoglutamarate	0.004213	0.002579	0.005109	0.012997	0.010237	0.0073	0.006583	0.004415	0.003656	0.003536	0.004374	0.00367	0.004585	0.005283	0.002672	0.004102	0.00385	0.004362
2-Oxoglutaric acid	0.000645	0.000616	0.000539	0.000455	0.000509	0.000347	0.000584	0.000551	0.000426	0.000336	0.000396	0.000384	0.000382	0.000408	0.000358	0.000358	0.000366	0.000345
3,5-Diiodo-L-tyrosine	0.001043	0.000745	0.000751	0.001008	0.00353	0.000484	0.000921	0.000726	0.002095	0.001169	0.001795	0.000629	0.000903	0.000575	0.001208	0.000772	0.00044	0.001381
3-Hydroxybenzoic acid	0.00043	0.000285	0.000374	0.000523	0.000428	0.000266	0.000702	0.000536	0.000562	0.000458	0.000376	0.000471	0.000392	0.000424	0.000402	0.000664	0.000499	0.000615
3-Methyl-2-oxopentanoic	0.000838	0.000815	0.000903	0.000923	0.000916	0.000576	0.001153	0.000958	0.00095	0.000856	0.000911	0.000863	0.000698	0.000864	0.000634	0.000973	0.000897	0.000995
4-Aminobutyric acid (GAB	0.000521	0.000442	0.00061	0.000617	0.000511	0.000351	0.000635	0.000557	0.000487	0.000234	0.000329	0.000242	0.000227	0.000249	0.000147	0.000266	0.000187	0.000184
4-Hydroxyphenylacetic aci	0.001573	0.002143	0.001905	0.002588	0.001972	0.001866	0.000964	0.001068	0.000816	0.001054	0.001182	0.001264	0.001118	0.001793	0.000877	0.000759	0.000737	0.000754
9-Heptadecenoic acid (C1	0.00119	0.000936	0.001132	0.001481	0.001223	0.000873	0.001833	0.002187	0.001433	0.001437	0.001489	0.001513	0.001115	0.001209	0.001033	0.00179	0.001563	0.001763
Adipic acid	0.000191	0.000169	0.000211	0.000283	0.000315	0.000252	0.000538	0.000216	0.000358	0.000238	0.000203	0.000185	0.000259	0.000265	0.000407	0.000184	0.000171	0.000163
Alanine	0.131747	0.149054	0.147751	0.376758	0.230366	0.214022	0.162866	0.163305	0.126525	0.139669	0.133122	0.133649	0.158422	0.262527	0.12592	0.135499	0.128164	0.135494
Arachidic acid (C20_0)	0.002823	0.002424	0.002369	0.003189	0.002838	0.001791	0.005209	0.004777	0.003809	0.002674	0.002925	0.003301	0.002663	0.002716	0.002394	0.004792	0.004046	0.004254
Arachidonic acid (C20_4n-	0.002185	0.001874	0.002159	0.001694	0.001739	0.00105	0.003572	0.003239	0.002332	0.002527	0.002862	0.002934	0.001597	0.001833	0.001658	0.003245	0.002912	0.003033
Aspartic acid	0.018026	0.018791	0.019773	0.060035	0.039549	0.033116	0.029897	0.030289	0.023413	0.016637	0.017715	0.017747	0.020866	0.03122	0.01837	0.023411	0.023125	0.023656
Azelaic acid	0.000379	0.000424	0.00047	0.000727	0.000767	0.000474	0.000821	0.000707	0.000725	0.000303	0.000423	0.000316	0.000703	0.000714	0.000688	0.000694	0.000507	0.000577
Benzoic acid	0.012796	0.012269	0.010836	0.016587	0.019089	0.010557	0.010137	0.010523	0.007321	0.008435	0.008485	0.008148	0.005356	0.00498	0.006482	0.010483	0.006217	0.008303
beta-Alanine	0.001729	0.001709	0.001941	0.00207	0.00224	0.001761	0.002311	0.002015	0.001973	0.002063	0.002564	0.002021	0.001774	0.001915	0.001686	0.002042	0.002028	0.001984
BHT (Antioxidant)	0.001019	0.00049	0.001212	0.002541	0.001861	0.001625	0.001218	0.001902	0.002107	0.001096	0.001302	0.00174	0.001136	0.00098	0.001738	0.001311	0.001792	0.001111
bishomo-gamma-Linolenic	0.001093	0.000964	0.001025	0.001076	0.001151	0.00056	0.001836	0.001727	0.001294	0.0012	0.001405	0.001386	0.00097	0.001031	0.000837	0.001669	0.001521	0.001436
Cabamic acid (From old lib	0.00036	0.000335	0.00035	0.000579	0.000407	0.000345	0.00046	0.000494	0.00043	0.000406	0.00059	0.000461	0.000468	0.000409	0.000393	0.000483	0.000402	0.000573
Caprylic acid (From old lib	0.001374	0.001603	0.001278	0.002769	0.002233	0.001592	0.001568	0.001641	0.004467	0.001372	0.001742	0.001305	0.001443	0.001521	0.001976	0.001787	0.001701	0.001732
cis-Aconitic acid	0.000938	0.000964	0.001067	0.001088	0.001104	0.000537	0.001886	0.001559	0.001223	0.001146	0.001182	0.001129	0.000788	0.000822	0.000819	0.001349	0.001278	0.001343
Citraconic acid	0.012511	0.007247	0.011636	0.016807	0.013889	0.010379	0.013849	0.014372	0.01102	0.013823	0.016711	0.01196	0.012147	0.013624	0.010058	0.015085	0.008617	0.014099
Citric acid	0.013156	0.01393	0.014444	0.013999	0.013443	0.00807	0.023579	0.026485	0.017043	0.016161	0.016355	0.01605	0.009964	0.011786	0.012794	0.021123	0.019235	0.019077
Citric acid secondary peak	0.000476	0.000493	0.000514	0.000543	0.000665	0.000308	0.001065	0.000947	0.000622	0.000658	0.000641	0.000581	0.000531	0.000406	0.000544	0.00071	0.000662	0.000701
Creatinine	0.071165	0.086485	0.084106	0.060738	0.040839	0.102364	0.065701	0.06683	0.069712	0.094166	0.05609	0.091818	0.070265	0.129085	0.114186	0.095294	0.094833	0.091657
Cysteine	0.024531	0.018395	0.031275	0.03208	0.037257	0.017062	0.041178	0.03396	0.031269	0.028267	0.039831	0.02516	0.025388	0.023405	0.020625	0.033607	0.025859	0.02835
Decanoic acid (C10_0)	0.000998	0.000852	0.001088	0.001264	0.001174	0.001214	0.001149	0.001197	0.004578	0.001101	0.001258	0.000983	0.000703	0.000643	0.001643	0.001033	0.001046	0.001185
Dehydroabietic acid (From	0.00013	0.000174	0.000123	0.000272	0.000199	0.000177	0.00015	0.000179	0.000116	0.000187	0.000193	0.000186	7.19E-05	0.000106	0.000165	0.00013	0.000219	0.000168
DL-3-Aminoisobutyric acid	0.000856	0.000706	0.000826	0.001014	0.000879	0.000602	0.001148	0.001476	0.000955	0.000965	0.000985	0.000965	0.001039	0.000818	0.000988	0.001257	0.001162	0.001126
Dodecanoic acid (C12_0)	0.003065	0.002679	0.002982	0.004015	0.003346	0.003042	0.004042	0.004409	0.00336	0.003221	0.004087	0.003556	0.003735	0.002739	0.004853	0.003655	0.003624	0.003711
EPA (C20_5n-3,6,9,12,15c)	0.000961	0.000781	0.000922	0.000845	0.000848	0.000528	0.001434	0.001273	0.000971	0.00112	0.001242	0.001177	0.000724	0.000812	0.000777	0.001505	0.001319	0.001247
Gallic acid	0.024315	0.026349	0.026859	0.045443	0.031906	0.047243	0.028586	0.020951	0.029028	0.02381	0.02192	0.024374	0.028681	0.02934	0.040622	0.023832	0.019768	0.021094
gamma-Linolenic acid (C1	0.037965	0.036185	0.035623	0.037881	0.033771	0.019398	0.062866	0.059207	0.048354	0.04472	0.047349	0.045877	0.032136	0.036284	0.035626	0.059031	0.055377	0.050392
Glutamic acid	0.056315	0.055266	0.058236	0.094488	0.088384	0.06163	0.083354	0.07437	0.058372	0.054114	0.063523	0.052702	0.05601	0.063483	0.048802	0.065653	0.062389	0.062703
Glutamine	0.029974	0.021755	0.030656	0.048272	0.041277	0.038613	0.042702	0.039982	0.030777	0.02906	0.037519	0.032991	0.036067	0.045426	0.031772	0.039048	0.04497	0.039945
Glutaric acid	0.000961	0.001313	0.0011	0.001435	0.001137	0.001124	0.00125	0.0014	0.001187	0.001224	0.001163	0.001268	0.001264	0.001478	0.001152	0.001189	0.00123	0.001305
Glutathione	0.015293	0.019805	0.016922	0.019672	0.013967	0.020171	0.017786	0.016053	0.0158	0.016693	0.013009	0.016695	0.01659	0.019014	0.01922	0.018154	0.017318	0.016014
Glyceric acid	0.001049	0.00062	0.001482	0.001561	0.001695	0.001	0.001459	0.001076	0.001189	0.001257	0.001714	0.001126	0.001252	0.001323	0.000918	0.001338	0.00089	0.001138
Glycine	0.351499	0.379038	0.354684	0.436024	0.368367	0.408679	0.41378	0.379542	0.337735	0.335449	0.364862	0.358798	0.337824	0.398632	0.355476	0.426793	0.408731	0.391308
Glyoxylic acid	0.001013	0.001022	0.001056	0.001578	0.00102	0.001277	0.000941	0.001137	0.001071	0.000838	0.00093	0.000963	0.001177	0.001086	0.001169	0.0012	0.001185	0.001247
Hexanoic acid (C6_0)	0.001745	0.001506	0.001623	0.003106	0.002746	0.001901	0.003098	0.002986	0.003614	0.001913	0.00202	0.001854	0.002276	0.002108	0.002853	0.002739	0.002195	0.002733
Hippuric acid	0.009087	0.008219	0.009517	0.010941	0.007912	0.005212	0.021429	0.020345	0.015221	0.013954	0.015447	0.015848	0.012253	0.013363	0.011862	0.019558	0.018997	0.017856
Histidine	0.018945	0.012812	0.0217	0.023574	0.020066	0.021025	0.024798	0.021677	0.018697	0.019437	0.019816	0.020562	0.020026	0.023212	0.02019	0.023039	0.023032	0.020469
Isoleucine	0.513033	0.523399	0.529098	0.664229	0.539387	0.565026	0.636098	0.584787	0.50061	0.499887	0.556557	0.510148	0.53766	0.532075	0.546411	0.595391	0.557385	0.533721
Itaconic acid	0.000234	0.000159	0.000157	0.000191	0.000262	0.000139	0.000341	0.000347	0.000232	0.000186	0.000245	0.00019	0.000205	0.000137	0.000159	0.000317	0.00024	0.000279
Lactic acid	0.251326	0.282096	0.243479	0.586895	0.408196	0.381282	0.239604	0.219356	0.203342	0.189489	0.177763	0.196091	0.205498	0.290063	0.206054	0.237708	0.215378	0.211395
Leucine	1.379239	1.284097	1.305356	1.65973	1.350236	1.370725	1.626098	1.479651	1.178092	1.315504	1.412141	1.401983	1.323565	1.4229	1.323955	1.53265	1.420742	1.382326
Linoleic acid (C18_2n-6,9c	0.14902	0.140668	0.143838	0.152278	0.137235	0.08138	0.233537	0.21763	0.182727	0.174904	0.170856	0.187039	0.128754	0.146423	0.130818	0.229738	0.214518	0.192529
Lysine	0.850519	0.824959	0.842569	1.039651	0.910849	0.973316	1.015854	0.970446	0.787509	0.828707	0.932179	0.892758	0.896101	0.95449	0.854063	1.012292	0.942	0.935116
Malic acid peak 2	0.000563	0.000554	0.00051	0.000878	0.00068	0.000574	0.000728	0.00067	0.000625	0.000537	0.000641	0.000464	0.000513	0.000678	0.000554	0.000709	0.000629	0.000649
Malonic acid	0.000416	0.000353	0.000379	0.000487	0.000513	0.000313	0.000517	0.000437	0.000443	0.000373	0.00044	0.000344	0.000347	0.000344	0.000312	0.00037	0.000302	0.000442
Margaric acid (C17_0)	0.010146	0.008787	0.009687	0.011174	0.010282	0.006029	0.018262	0.01715	0.01423	0.011274	0.01172	0.01267	0.009911	0.011018	0.009873	0.017842	0.015744	0.01512
Methionine	0.024117	0.044718	0.014748	0.022972	0.008332	0.031663	0.037279	0.050868	0.019833	0.026243	0.015117	0.036911	0.018736	0.036307	0.03618	0.047835	0.055749	0.041784
Myristic acid (C14_0)	0.007147	0.005843	0.00676	0.007848	0.007199	0.004455	0.011818	0.011723	0.010201	0.007938	0.008663	0.008285	0.007059	0.006869	0.007796	0.011372	0.009646	0.009466
NADP_NADPH	0.000103	9.06E-05	9.24E-05	9.61E-05	7.32E-05	0.00012	6.67E-05	8.85E-05	9.2E-05	0.000104	0.000113	9.73E-05	4.97E-05	7.38E-05	0.000115	7.85E-05	6.53E-05	7.49E-05
Nicotinamide	0.002472	0.003199	0.003445	0.004084	0.002938	0.003343	0.003679	0.003513	0.003835	0.003444	0.003315	0.003519	0.003146	0.003539	0.003341	0.003687	0.003922	0.004211
Nicotinic acid	0.005371	0.004669	0.005926	0.016064	0.011125	0.01118	0.002034	0.002954	0.002311	0.003493	0.004429	0.003609	0.00587	0.009505	0.003727	0.002083	0.001673	0.002201
Ornithine	0.183016	0.203789	0.191398	0.364068	0.316067	0.265512	0.069238	0.08515	0.054514	0.062542	0.076624	0.070687	0.056998	0.092048	0.043342	0.047319	0.054616	0.054644
Oxalic acid	0.003393	0.002374	0.003781	0.009096	0.005952	0.005126	0.003689	0.003181	0.003567	0.003681	0.005338	0.003286	0.004027	0.00432	0.003593	0.003152	0.002737	0.003666
Palmitelaidic acid (C16_1n	0.002029	0.001678	0.001943	0.002507	0.002235	0.001098	0.003727	0.003743	0.002739	0.002372	0.002338	0.002427	0.00201	0.002179	0.002023	0.003342	0.002868	0.002819
para-Toluic acid	0.000607	0.000702	0.000653	0.00073	0.000586	0.000561	0.000628	0.000511	0.000588	0.000603	0.000722	0.000636	0.000466	0.000586	0.000665	0.000458	0.00042	0.000441
Pentadecanoic acid (C15_	0.001897	0.00196	0.001663	0.002268	0.001854	0.001224	0.003373	0.003029	0.002462	0.002406	0.002438	0.002294	0.001881	0.001949	0.002124	0.003027	0.002618	0.002574
Phenylalanine	0.42402	0.411202	0.416734	0.520107	0.431427	0.444851	0.499177	0.472929	0.384789	0.408457	0.420915	0.424095	0.407605	0.450708	0.412109	0.481178	0.451647	0.437529
Proline	0.066926	0.071507	0.073586	0.130062	0.090566	0.065435	0.093902	0.088057	0.069895	0.067112	0.069095	0.071696	0.070235	0.089052	0.060943	0.077497	0.076647	0.070407
Putrescine	0.000548	0.000802	0.000612	0.00192	0.001275	0.001563	0.000214	0.000297	0.000191	0.000332	0.000349	0.000286	0.000394	0.000806	0.000242	0.000288	0.000238	0.000248
Pyroglutamic acid	0.497809	0.502002	0.512173	0.595332	0.480277	0.514605	0.566341	0.523983	0.45691	0.493342	0.526203	0.523063	0.474207	0.511498	0.461741	0.542894	0.516097	0.484651
S-Adenosylmethionine	0.004868	0.004972	0.004958	0.014654	0.010451	0.008897	0.006363	0.00521	0.003772	0.003937	0.003932	0.003781	0.004666	0.00528	0.003768	0.004926	0.004916	0.003929
Salicylic acid	0.000328	0.000252	0.00036	0.000477	0.000351	0.000242	0.000369	0.000326	0.000484	0.000406	0.000397	0.000333	0.000283	0.000301	0.000493	0.000482	0.000349	0.000369
Serine	0.045938	0.046642	0.048125	0.059578	0.047446	0.054708	0.059047	0.055751	0.044706	0.051713	0.047268	0.054886	0.049024	0.061604	0.054607	0.060095	0.057354	0.054909
Stearic acid (C18_0)	0.310035	0.290378	0.290633	0.347054	0.306869	0.191677	0.6	0.561531	0.483289	0.352516	0.363588	0.378892	0.31197	0.337963	0.332238	0.592574	0.515114	0.492558
Succinic acid	0.006266	0.006269	0.005681	0.006525	0.006835	0.005089	0.005515	0.005349	0.00493	0.002467	0.00247	0.002544	0.002312	0.003223	0.002103	0.002646	0.002216	0.00277
Threonine	0.356949	0.341317	0.376246	0.447835	0.378803	0.398737	0.453171	0.416091	0.34362	0.363265	0.427792	0.403569	0.372245	0.415668	0.369675	0.434443	0.40403	0.406076
trans-Cinnamic acid	0.000117	4.1E-05	5.93E-05	8.51E-05	9.58E-05	2.99E-05	0.000107	0.000156	8.86E-05	7.05E-05	0.000123	0.000112	0.000132	0.000113	9.14E-05	0.000128	8.16E-05	7.04E-05
trans-Vaccenic acid	0.040484	0.03826	0.037217	0.043755	0.039324	0.022753	0.06378	0.061641	0.050721	0.045796	0.047257	0.04663	0.039424	0.041931	0.038728	0.064573	0.05674	0.054876
Tryptophan	0.160381	0.157751	0.1583	0.195444	0.17895	0.195823	0.230274	0.21448	0.158423	0.160771	0.187307	0.200386	0.154305	0.225188	0.195405	0.217125	0.244194	0.20843
Tyrosine	0.518339	0.526298	0.51681	0.638358	0.549764	0.608808	0.602439	0.596657	0.493718	0.50237	0.571971	0.542666	0.522645	0.60518	0.573232	0.598464	0.611698	0.548837
Unknown 045(100) 59(89	0.013315	0.009685	0.01535	0.01704	0.020427	0.011488	0.017546	0.012945	0.016334	0.013513	0.02009	0.012169	0.014766	0.014169	0.01171	0.013536	0.010307	0.013132
Unknown 070(100) 42(28	0.001027	0.001065	0.001083	0.002405	0.001093	0.001028	0.001106	0.00086	0.000993	0.000992	0.00072	0.000653	0.001258	0.000979	0.000716	0.000871	0.000557	0.000708
Unknown 043(100) 85(71	0.000374	0.000459	0.000492	0.000916	0.000854	0.000669	0.0008	0.000573	0.000413	0.000409	0.000558	0.000518	0.000364	0.0004	0.000344	0.000542	0.000413	0.00051
Unknown 045(100) 82(94	0.000217	0.000222	0.000229	0.000272	0.000299	0.000176	0.00031	0.000367	0.000394	0.000216	0.000227	0.000259	0.000235	0.00024	0.000302	0.00036	0.00032	0.000295
Unknown 056(100) 59(24	0.003824	0.003332	0.004359	0.004567	0.002919	0.002878	0.005063	0.002876	0.003637	0.003901	0.002472	0.002368	0.00391	0.00218	0.00318	0.003548	0.002167	0.00235
Unknown 059(100) 116(9	0.00146	0.000759	0.002062	0.002167	0.002942	0.001404	0.002305	0.001615	0.001812	0.001522	0.003185	0.00166	0.001869	0.001833	0.001228	0.001724	0.001448	0.001625
Unknown 059(100) 81(40	0.006127	0.004608	0.00519	0.005649	0.00531	0.005784	0.007832	0.011526	0.005751	0.00816	0.00732	0.007047	0.005533	0.00624	0.005527	0.009007	0.005734	0.009568
Unknown 069(100) 57(39	0.003075	0.002536	0.002834	0.004557	0.00246	0.002592	0.003621	0.003948	0.002645	0.003543	0.003917	0.003238	0.00316	0.002531	0.002369	0.00399	0.002559	0.003333
Unknown 073(100) 281(8	0.000738	0.000645	0.000615	0.000819	0.002926	0.000396	0.000694	0.000593	0.001812	0.001048	0.00137	0.000459	0.000827	0.000656	0.001078	0.000753	0.000289	0.000954
Unknown 073(100) 429(7	0.014057	0.013334	0.015589	0.023117	0.020165	0.017353	0.017268	0.018839	0.024038	0.017272	0.018837	0.01565	0.005086	0.003308	0.036714	0.01755	0.015479	0.020084
Unknown 074(100) 46(21	0.012129	0.013731	0.013364	0.011191	0.008269	0.010642	0.01237	0.008713	0.012869	0.012204	0.007095	0.007356	0.012692	0.007242	0.010954	0.009495	0.006536	0.007656
Unknown 074(100) 57(67	0.001535	0.001342	0.001407	0.002024	0.001385	0.001406	0.001391	0.001344	0.001575	0.001324	0.001687	0.001443	0.001282	0.001538	0.001695	0.001425	0.001378	0.001594
Unknown 074(100) 87(68	0.001124	0.001268	0.000982	0.00079	0.000925	0.000751	0.000784	0.000832	0.000636	0.000329	0.000284	0.000307	0.000432	0.000362	0.000394	0.000403	0.000371	0.000317

Unknown 081(100) 136(5	0.029646	0.031198	0.026787	0.040346	0.025807	0.034343	0.032816	0.032382	0.021903	0.027653	0.023843	0.029052	0.028874	0.026651	0.030346	0.034175	0.028911	0.027057
Unknown 082(100) 153(9	0.004409	0.002207	0.005089	0.00477	0.003241	0.002499	0.00549	0.00273	0.004125	0.003871	0.002539	0.002013	0.004662	0.001893	0.002566	0.004054	0.001581	0.002036
Unknown 083(100) 111(7	0.002041	0.001539	0.001996	0.00248	0.002473	0.002835	0.002661	0.002702	0.002175	0.002368	0.002623	0.0027	0.002315	0.00338	0.002017	0.002414	0.002856	0.002518
Unknown 084(100) 126(4	0.000878	0.001097	0.001159	0.001333	0.001053	0.001366	0.001156	0.000796	0.000828	0.000811	0.000676	0.000743	0.000802	0.000841	0.000735	0.001086	0.000778	0.000929
Unknown 088(100) 43(72.	0.000314	0.000263	0.000259	0.000391	0.00026	0.000323	0.000231	0.000158	0.000243	0.00019	0.000187	0.000245	0.000238	0.000244	0.000197	0.000308	0.000254	0.000265
Unknown 088(100) 44(30.	0.047819	0.047419	0.049194	0.06188	0.035112	0.042762	0.059569	0.042299	0.040197	0.04313	0.032313	0.029657	0.047238	0.027369	0.041628	0.04904	0.02266	0.030156
Unknown 088(100) 57(53.	0.000647	0.000417	0.000532	0.000848	0.000707	0.000289	0.001131	0.000783	0.000759	0.000804	0.000929	0.000655	0.000561	0.000626	0.0005	0.000868	0.000809	0.000963
Unknown 091(100) 106(5	0.000416	0.000502	0.000549	0.000657	0.000489	0.000538	0.000569	0.000569	0.000538	0.000489	0.000458	0.000451	0.000485	0.000574	0.000611	0.000551	0.000624	0.00052
Unknown 091(100) 150(3	0.004449	0.004937	0.005265	0.006631	0.005148	0.00408	0.00394	0.004119	0.003637	0.003999	0.004076	0.004165	0.003558	0.005085	0.00319	0.003587	0.003418	0.003498
Unknown 091(100) 92(23.	0.00035	0.000156	0.000268	0.000435	0.000476	0.000327	0.000307	0.000459	0.000237	0.000321	0.000471	0.000572	0.000305	0.000461	0.000345	0.000473	0.000409	0.000562
Unknown 095(100) 126(3	0.00043	0.000367	0.000416	0.00054	0.00038	0.000307	0.000461	0.000503	0.000384	0.000536	0.000349	0.000488	0.000514	0.000443	0.000464	0.000541	0.0004	0.000317
Unknown 112(100) 43(60.	0.011037	0.013753	0.012553	0.01309	0.008063	0.010193	0.014192	0.008403	0.010982	0.01012	0.006796	0.006317	0.012189	0.00636	0.009959	0.010515	0.006559	0.005739
Unknown 114(100) 59(15.	0.187457	0.119478	0.2167	0.227362	0.226563	0.189054	0.224787	0.180899	0.191724	0.170306	0.236483	0.172394	0.209221	0.190178	0.171739	0.201344	0.168285	0.187936
Unknown 114(100) 59(61.	0.026961	0.030801	0.029221	0.029444	0.021709	0.038063	0.028571	0.027472	0.026969	0.032167	0.022402	0.032363	0.028495	0.04186	0.037109	0.033963	0.036676	0.031726
Unknown 115(100) 100(7	0.001818	0.00168	0.001548	0.002609	0.001858	0.001954	0.002248	0.002214	0.001702	0.002122	0.002465	0.002283	0.001874	0.002273	0.001624	0.002359	0.00202	0.002431
Unknown 115(100) 59(65.	0.000514	0.000581	0.000611	0.000555	0.000677	0.000366	0.000796	0.000838	0.000609	0.000641	0.000601	0.000614	0.000453	0.000535	0.000658	0.000758	0.000654	0.00061
Unknown 126(100) 127(4	9.87E-05	0.000123	0.000128	0.000173	0.000176	8.4E-05	0.000243	0.000197	0.000146	0.00016	0.000179	0.000191	0.000106	9.55E-05	0.00016	0.000205	0.000148	0.000172
Unknown 128(100) 139(2	0.003217	0.002475	0.00347	0.006907	0.004892	0.003275	0.001254	0.001223	0.00105	0.001295	0.001281	0.000789	0.001203	0.001022	0.00074	0.000955	0.000531	0.000718
Unknown 128(100) 42(10.	0.00058	0.000714	0.000712	0.002699	0.001505	0.000909	0.000961	0.000855	0.000557	0.000623	0.000663	0.00061	0.000545	0.000837	0.000533	0.000705	0.000847	0.000636
Unknown 128(100) 42(11.	7.97E-05	0.000107	6.48E-05	0.000111	7.97E-05	8.6E-05	0.000101	0.000115	9.53E-05	6.61E-05	0.0001	7.75E-05	8.23E-05	0.000119	5.8E-05	9.95E-05	0.000138	0.000106
Unknown 130(100) 189(1	0.000347	0.000421	0.000341	0.000486	0.00041	0.000339	0.00052	0.000518	0.000384	0.000406	0.000483	0.000709	0.000341	0.000447	0.000387	0.000585	0.000516	0.000528
Unknown 135(100) 180(2	0.000825	0.000937	0.00101	0.00096	0.000627	0.000656	0.000666	0.000647	0.000562	0.000543	0.000512	0.000555	0.000526	0.000599	0.000411	0.000597	0.000575	0.000611
Unknown 142(100) 153(5	0.037745	0.026927	0.037126	0.048039	0.032046	0.028611	0.04625	0.031856	0.029828	0.036032	0.027645	0.023117	0.036434	0.021836	0.025744	0.040777	0.017338	0.02621
Unknown 142(100) 59(19.	0.017016	0.017665	0.019295	0.018393	0.015393	0.027125	0.019688	0.017711	0.01638	0.021736	0.016144	0.022764	0.018145	0.028693	0.022519	0.021727	0.024987	0.023877
Unknown 149(100) 167(3	0.001104	0.000718	0.001023	0.001426	0.001002	0.000741	0.001145	0.00124	0.00154	0.000843	0.001294	0.001094	0.00081	0.001218	0.000944	0.001002	0.001162	0.001297
Unknown 156(100) 96(36.	0.00046	0.000354	0.000538	0.001136	0.001592	0.000598	0.000827	0.000617	0.000593	0.000433	0.000614	0.000493	0.001119	0.000956	0.000908	0.000718	0.000514	0.000578
Unknown 161(100) 236(8	0.00039	0.000368	0.000382	0.000438	0.000564	0.000273	0.000639	0.000502	0.000388	0.000368	0.000445	0.000429	0.000397	0.000363	0.000345	0.000563	0.00052	0.000529
Unknown 202(100) 158(4	0.000117	6.36E-05	8.02E-05	6.43E-05	9.72E-05	6.53E-05	0.000322	0.000198	0.000193	0.0001	0.000141	0.000111	9.51E-05	0.000149	0.000126	0.00026	0.000242	0.000235
Unknown 249(100) 57(31.	0.000983	0.001368	0.001177	0.001291	0.001323	0.00129	0.001685	0.001612	0.001579	0.001288	0.001352	0.001337	0.001092	0.001221	0.001223	0.001741	0.0014	0.001363
Unknown 281(100) 282(2	0.010655	0.010323	0.008995	0.012729	0.00925	0.008335	0.010457	0.011314	0.009682	0.010431	0.010089	0.008422	0.009302	0.006778	0.010748	0.011476	0.005869	0.009622
Unknown 355(100) 73(67.	0.002089	0.002005	0.001715	0.002386	0.004242	0.001191	0.002103	0.001987	0.004041	0.002408	0.003396	0.00146	0.001857	0.001396	0.003097	0.001921	0.001248	0.002049
Valine	1.325029	1.302595	1.330628	1.706412	1.381604	1.413472	1.617317	1.480862	1.327641	1.321372	1.410007	1.365083	1.349964	1.425563	1.369182	1.547759	1.405997	1.421163

Legend

All	T. vaginalis and BV bacteria co-cultures.
Tv	T. vaginalis axenic cultures
8, 16, 32	Time points of 6, 16 and 32h.
a, b, c	Triplicates

Name	All-16a	All-16b	All-16c	All-32a	All-32b	All-32c	All-8a	All-8b	All-8c	Tv-16a	Tv-16b	Tv-16c	Tv-32a	Tv-32b	Tv-32c	Tv-8a	Tv-8b	Tv-8c
2-Hydroxyl	2.605972	2.731941	1.212978	1.894379	4.336035	4.745444	1.247974	0.553825	2.171455	1.68254	1.682421	0.50954	0.581655	0.52375	0.920511	0.899251	1.153488	1.025856
10.5921 mi	8.28842	9.24369	3.404326	20.91167	34.71322	40.82916	2.463533	1.519296	2.069509	4.059524	4.120232	1.39899	2.857049	2.73671	6.653367	2.097345	2.470543	2.476406
Glyceric ac	7.202476	8.802437	8.492512	5.555899	7.779302	6.718679	7.591572	5.852404	8.430955	6.765873	6.916252	3.489899	4.038529	3.658069	4.675499	7.254595	6.621705	8.116354
L-Threonin	119.3299	141.2567	63.65391	52.16553	95.3217	82.15034	78.71637	36.20311	82.31325	168.8889	129.6451	31.61841	31.29947	50.55175	36.2793	75.91014	101.9535	68.76794
Aminomal	16.69337	14.94865	25.82363	13.0945	22.58354	24.97494	20.83144	17.89844	8.376274	15.89881	21.76451	18.57464	15.70053	21.21925	16.46633	12.50374	10.48217	13.99871
Malic acid	0.414421	0.430809	0.334027	0.219271	0.387157	0.34795	0.495948	0.228504	0.34569	0.434524	0.512438	0.201459	0.172942	0.24945	0.22288	0.349898	0.369767	0.378151
2,3,4-Trihy	0.760379	0.670148	0.375624	0.34404	0.469451	0.404897	0.67423	0.2478	0.512512	0.855159	0.810945	0.304994	0.249781	0.328091	0.300187	0.392784	0.568992	0.475113
Xylose Oxi	12.60306	13.22019	17.84027	11.15256	19.38155	21.3303	12.94165	13.7197	9.491196	15.31151	22.14925	16.83053	12.80385	13.54137	13.12968	12.88496	10.99612	14.85714
Arabinose	6.227968	4.73107	2.40599	2.591106	3.305486	2.863326	3.04376	1.821598	3.062095	7.137897	5.201493	2.123737	1.308669	1.98553	2.100374	2.556841	4.04031	4.623788
Ribose Oxi	4.617626	4.359443	2.678869	2.183447	5.942643	4.044989	3.517018	1.820582	3.523633	4.356151	3.718905	1.389731	1.515981	2.181818	1.909601	3.133424	4.000775	3.341306
Cyclonona	9.671522	15.37337	16.40932	13.53428	17.72569	19.14351	13.59968	14.82194	12.04263	14.78175	23.17081	16.23345	12.33275	13.96414	13.49626	17.06739	14.57364	14.77957
Tartaric aci	0.363438	0.381201	0.257488	0.16677	0.280549	0.246583	0.350081	0.220379	0.268767	0.330357	0.366501	0.152918	0.191988	0.184964	0.165524	0.196052	0.334884	0.361991
Asparagine	2.142025	0.939948	0.637687	0.276714	1.473815	1.67369	0.615883	0.529113	0.596849	0.572421	0.757048	0.982323	0.589974	0.586348	0.644327	0.471749	0.710078	0.52618
Fructose O	1.914057	3.571802	1.112313	0.652254	1.279302	1.569476	1.599676	0.94956	1.575533	6.914683	5.980929	1.641975	1.043126	1.641082	1.085411	1.633764	2.431783	2.197802
18.3679 mi	1.217043	2.395126	0.702995	0.554046	0.94202	0.830866	1.160454	0.541638	1.169601	4.154762	3.71476	1.143939	0.654772	1.034602	0.675499	1.130701	1.357364	1.190045
18.5292 mi	1.983977	4.076588	5.685108	3.444101	6.472569	6.085991	12.71475	5.17434	19.48471	3.203373	3.076285	1.040685	3.190236	4.240642	1.057045	6.059905	5.513178	2.747899
Glucopyrar	117.7451	154.7363	297.5042	225.2057	282.7332	366.9795	335.1442	282.5619	382.6951	183.873	433.937	278.8418	196.2872	226.9091	266.7332	269.3751	333.2465	242.265
L-Glutamin	3.513474	24.40383	3.246672	2.494132	13.31172	10.09112	2.380875	0.740691	1.46988	35.57143	26.8325	0.902918	0.846979	4.167977	1.03086	9.371001	5.364341	2.522948
L-Lysine, 4	44.61471	59.48825	19.29784	7.731933	34.26933	35.48519	21.16694	9.240352	13.83596	81.36508	57.18076	12.8193	8.269702	20.80151	10.69327	33.75357	39.56589	24.17582
Scyllo-Inos	0.86016	1.034813	0.750832	0.541692	1.110349	1.183941	0.972447	0.621869	0.913809	1.330357	1.138474	0.709877	0.52014	0.683234	0.593828	0.7597	0.976744	0.770524
Ascorbic ac	3.633649	2.067885	2.143927	1.429277	2.472569	2.915718	1.419773	1.907921	1.173309	4.175595	6.372305	4.186869	1.823336	1.633218	3.363466	1.671205	2.028682	3.455721
Pantotheni	2.083758	2.389904	1.394343	0.679432	1.75	1.692483	1.249595	0.947529	1.086191	2.608135	2.454395	0.982604	1.073774	1.339415	1.035536	1.263445	1.707752	1.604396
D-(-)-Fruct	0.766934	0.870322	1.232529	0.275479	0.832294	1.21754	0.883306	0.506093	0.535681	0.681548	1.170813	0.619529	0.866243	0.923875	0.776808	0.511232	0.777519	0.620556
.beta.-D-(+	0.372178	0.360313	1.068636	0.245213	0.368454	1.947039	1.141005	0.984766	0.44949	0.412698	0.728027	1.691919	2.150832	1.02548	1.27712	0.569775	0.786047	0.345184
Myo-Inosit	0.277495	0.461271	2.24584	0.444101	0.486284	2.436219	1.771475	1.592756	0.974977	0.330357	0.76534	2.794052	3.524081	1.629129	2.908042	1.020422	1.707752	0.497091
Adenosine	0.238165	0.24369	0.174709	0.122298	0.24813	0.228929	0.303079	0.137779	0.21038	0.257937	0.250415	0.115039	0.160902	0.150047	0.2048	0.187202	0.228682	0.196509
2-.alpha.-N	0.180626	0.191471	0.196755	0.100679	0.180798	0.202164	0.797407	0.525728	0.883225	0.209325	0.226368	0.135522	0.127846	0.149418	0.18298	0.878148	1.034109	0.908856
Guanosine	0.207575	0.209748	0.264975	0.091414	0.173317	0.212415	0.401945	0.157075	0.192771	0.251984	0.272803	0.175365	0.222198	0.199119	0.227244	0.182437	0.190698	0.221073
Pentanoic	0.490168	0.463011	0.31822	0.271155	0.465711	0.387813	0.573744	0.175017	0.244671	0.489087	0.390547	0.152357	0.14514	0.19157	0.161783	0.257318	0.283721	0.416936
L-Aspartic	3.658412	2.447346	4.626456	4.66399	4.990648	4.798405	1.883306	5.273189	3.224282	3.443452	5.411277	6.439955	3.943958	3.121736	4.283666	3.830497	3.713178	6.164189
Ribonic aci	0.329206	0.446475	0.24792	0.158122	0.279925	0.281321	0.371151	0.220379	0.306766	0.372024	0.432836	0.174242	0.184107	0.249135	0.24283	0.311777	0.435659	0.418229
Phosphoric	0.573926	0.619669	0.361481	0.274861	0.473815	0.468109	0.445705	0.292146	0.482854	0.590278	0.567993	0.237374	0.229641	0.324945	0.284289	0.411164	0.510853	0.679379
D-Allose, p	1653.092	1966.009	901.5374	1181.174	1377.037	1006.506	2281.18	678.0447	1678.829	2250.159	1477.413	499.0707	406.641	586.4083	535.4613	1135.183	1272.856	1370.849
Oxalic acid	0.615441	0.697998	0.292845	0.460161	0.400249	0.355923	0.764992	0.343263	0.665431	0.780754	0.466833	0.198092	0.186296	0.258257	0.225062	0.456093	0.509302	0.573368
L-Pipecolic	0.496723	0.492602	0.22629	0.256331	0.399626	0.370159	0.181524	0.145565	0.224282	0.325397	0.259536	0.150112	0.174037	0.224599	0.184227	0.145677	0.210078	0.172592
Pyroglutan	4.736344	2.995648	1.978785	2.8147	2.330424	2.429385	1.773096	2.228165	3.481928	3.092262	3.008292	1.739899	2.129378	1.868198	1.877182	1.994554	4.422481	3.128636
Butanoic a	0.147123	0.192341	0.067388	0.050649	0.13217	0.096811	0.110211	0.067028	0.100093	0.114087	0.089552	0.035073	0.037653	0.061969	0.048005	0.097345	0.090698	0.098255
1-Piperidin	0.466861	0.577023	0.343594	0.291538	0.335411	0.421982	0.799028	0.312119	0.63392	0.717262	0.767828	0.251684	0.200525	0.258886	0.244389	0.466984	0.496899	0.645766
L-Alanine,	16.33795	20.13577	6.020799	8.75664	17.22195	22.34169	8.594814	4.170616	8.387396	19.68254	13.65174	3.219416	3.623468	4.8229	7.129676	7.856365	10.07907	8.217195
L-Serine, 2	3.767662	5.24369	2.301165	1.760346	3.44389	3.666287	1.833063	1.498646	2.975904	6.933532	5.90796	2.078563	2.29838	3.40799	2.302369	4.174268	4.153488	5.270847
Propylene	0.539694	0.765883	0.344842	0.668314	1.419576	0.81549	0.183144	0.095125	0.267841	0.313492	0.325041	0.096521	0.332531	0.368669	0.335411	0.152485	0.165116	0.157725
Palmitic Ac	1.506191	1.83899	0.977953	0.810994	1.992519	1.724943	1.014587	0.818213	2.020389	2.237103	2.375622	0.889731	0.854422	1.012897	0.997195	1.653506	2.348062	1.914027
Stearic acid	0.831027	1.302002	0.576539	0.515133	1.03616	1.436219	0.9141	0.553148	1.565338	1.503968	1.393035	0.459877	0.534588	0.620006	0.645574	0.754935	1.18062	0.901099
Propanami	197.7334	250.1514	85.16473	86.09759	135.8204	114.6241	108.8104	53.60054	122.0093	307.2381	187.3831	38.94949	37.64623	73.16011	49.59601	117.4132	151.3178	118.9813
DL-Phenyla	9.009468	10.06789	4.276206	3.558369	6.326683	6.459567	2.497569	2.905213	6.202966	13.77679	11.73134	3.608586	3.76007	5.319912	3.267456	6.489449	6.355039	9.334842
41.3527 mi	0.531682	0.797215	0.646423	0.29895	0.770574	0.648064	0.721232	0.72478	1.243744	1.238095	1.659204	0.596521	0.564799	0.580686	0.658978	1.172226	1.452713	1.33161
L-2-Aminol	0.438456	0.573542	0.182612	0.13218	0.312968	0.390091	0.294976	0.166892	0.241891	0.882937	0.451907	0.159371	0.174256	0.193772	0.291459	0.294078	0.241085	0.264383
L-Proline, 2	4.055353	5.294169	1.119384	0.91538	2.34601	2.288724	1.831442	0.886594	2.175162	6.405754	3.431177	0.869529	0.653897	1.032086	0.947631	2.854323	2.817054	2.714932
DL-Ornithi	4.193736	6.978242	1.915973	2.256949	7.596633	6.603645	1.016207	0.265741	0.570899	2.468254	2.192371	0.320988	0.232706	0.408934	0.454489	0.706603	0.605426	0.588882
L-Valine, 2	385.0255	462.5379	179.7537	158.7548	290.3142	270.1048	212.953	114.1557	223.4365	553.3968	377.8972	106.8462	91.36252	149.7226	114.3741	211.6487	285.4202	226.1306
l-Norvaline	0	0	0	0	0	0	0	0.017942	0	0	0	0	0	0	0	0	0	0
D-Norleuci	0.080117	0.120975	0	0.050031	0.05985	0.052392	0	0	0	0.202381	0.211443	0.04349	0.022329	0.024851	0.035536	0.035398	0	0.055591
07.0684 mi	236.7487	262.2942	138.8885	184.7264	333.7656	343.7631	109.4457	71.97292	114.5653	228.8889	157.9834	57.53535	56.96673	69.19409	80.97756	94.84547	114.1085	122.746
1-(3-Methy	37.91988	56.18799	42.39601	23.78258	53.55112	33.85877	42.4376	27.33378	28.38184	45.63492	47.78773	30.00673	21.69877	23.72318	24.11721	33.36147	40.8124	42.97867
Hydracrylic	0.603059	0.690165	0.526206	0.256949	0.396509	0.447039	0.507293	0.371699	0.266914	0.680556	1.044776	0.22138	0.180166	0.34665	0.279925	0.355344	0.404651	0.559147
Formamide	0.444283	0.658834	0.457571	0.358863	0.340399	0.443622	0.583468	0.415369	0.483781	0.861111	0.589552	0.23064	0.223511	0.307329	0.330424	0.352621	0.515504	0.669683
4-Hydroxyl	0.390386	0.412533	0.406822	0.190859	0.382793	0.236333	0.452188	0.207177	0.276182	0.463294	0.378109	0.161055	0.196585	0.21642	0.229426	0.285228	0.311628	0.443439
Butanedioi	2.262928	1.669278	1.231281	0.880791	1.814838	1.265376	1.128039	0.81652	1.310473	2.087302	1.929519	0.801066	0.662872	0.882668	0.979426	1.443159	1.41938	1.760181
Urea, 2TM	180.2535	227.2028	126.2696	42.31748	141.0474	117.8679	162.7618	116.2789	173.5533	356.7619	268.8955	87.56004	73.63923	105.0293	80.61845	186.9462	207.1318	215.3329
N,O-Bis(ter	12.44574	12.9852	21.12146	10.84126	19.41646	20.31891	16.59806	14.03927	7.993513	11.75794	17.1476	15.23681	13.48511	17.19786	13.34913	9.872022	10.81163	12.42146
1,4-Butane	36.87109	46.79547	15.72047	16.04941	24.6384	20.64237	19.1799	10.10968	22.11677	55.85714	33.85738	7.216611	6.777583	12.90972	9.129676	21.39142	27.36124	22.80026
3-Hydroxyl	1.653314	1.557876	0.958819	0.455219	1.426434	1.096241	1.388979	0.869668	1.348471	2.384921	1.989221	0.708474	0.54028	0.832023	0.74283	1.041525	1.44031	1.910795
((5R,8R,8a	0.04807	0	0.054908	0	0	0	0	0.028097	0	0.069444	0.077944	0.017677	0.023205	0.016986	0	0	0	0.071105
L-5-Oxopro	1245.879	1482.973	747.1281	666.0111	976.7581	904.4556	749.2253	487.2146	787.7516	1652.317	1317.784	502.5185	410.1156	548.5599	432.0399	789.435	968.4341	862.159
Pipecolic a	0.164603	0.120975	0.163062	0.040148	0.081047	0.126993	0.166937	0.096479	0.144578	0.198413	0.171642	0.07688	0.075744	0.181818	0.114713	0.11096	0.168992	0.255333
L-Leucine,	407.5863	479.9164	180.7654	157.3317	301.6858	274.2961	209.6467	111.3609	214.2428	585.6508	400.1327	106.523	89.77933	157.6747	114.4539	222.1484	299.2124	227.0407
L-Norleucir	0.155863	0.087032	0.084027	0.039531	0.129676	0.132688	0.097245	0.054841	0	0.134921	0.074627	0.050505	0.038967	0.075495	0.040835	0.044929	0	0.087912
N-Methyld	0.233795	0.349869	0.16015	0.422483	1.038653	1.120159	0.102107	0.050779	0.058387	0.159722	0.223051	0.082211	0.185201	0.272727	0.244701	0	0.04186	0.058177
Ethanolam	1.487254	1.925152	0.847338	0.883879	1.335411	1.343964	1.401945	0.773866	1.470806	2.164683	1.856551	0.684063	0.514229	0.754954	0.758728	1.614023	1.556589	1.262443
Glycine, 3T	152.0524	166.3777	86.09651	59.63187	117.7855	101.0296	105.6596	56.63372	107.4476	206.9841	151.6153	52.93827	44.91769	69.95911	51.91022	110.3989	142.8837	100.3232
Tyramine,	13.17407	14.15927	8.482529	7.882026	11.66085	10.65604	8.954619	6.050102	9.889713	17.42063	17.3864	6.244669	4.966725	7.353256	7.638404	12.86317	12.26899	10.67873
.beta.-Alan	0.822287	1.101828	0.558652	0.49475	0.966958	0.625854	0.76175	0.475626	0.693234	1.27877	0.989221	0.400112	0.318958	0.388487	0.417706	0.949626	0.845736	0.772463
4-Aminobu	0.300073	0.226284	0.096922	0.081532	0.179551	0.190774	0	0.107651	0.131603	0.119048	0.101161	0.030584	0.037434	0.035546	0.052681	0.044929	0.039535	0
N-(4-[Bis(t	18.15586	7.953003	5.205491	8.390982	6.632793	8.02221	5.110211	8.018957	16.21501	7.814484	7.704809	6.426487	10.64273	6.132746	6.735661	7.377127	11.47287	9.729153
N-.alpha.-A	0.093227	0.072237	0.050333	0	0.064838	0.106492	0	0.028775	0.056534	0.09127	0.126036	0.036195	0.039623	0.059453	0.048628	0.085773	0.053488	0.069813
4-Aminom	1.252731	0.939077	1.206323	0.591723	3.054863	4.460137	0.431118	0.409276	0.164968	0.345238	0.494196	0.354938	0.336033	0.312048	0.456983	0.206263	0.217054	0.202973
L-Methioni	25.38092	33.0792	12.94509	10.37678	20.34913	18.13667	11.82658	7.907921	16.91196	37.98413	28.19237	7.432099	7.523643	14.36427	8.458853	15.80395	21.14109	17.69619
1-Phenylet	1.169701	0.857267	0.68178	0.407659	1.495636	0.680524	0.225284	0.158768	0.18721	0.421627	0.286899	0.108305	0.084939	0.143127	0.095387	0.195371	0.11938	0.171299
Niacinamic	1.462491	2.368146	1.366057	0.193947	0.350374	0.178246	1.893031	1.275897	1.689527	3.75	3.047264	1.207632	0.955779	1.525008	0.665835	2.2403	2.551938	2.813187
N-Acetylty	2.222141	2.312446	4.144759	1.718962	1.893392	1.5541	1.747164	3.487136	2.043559	2.001984	3.902156	5.535354	3.782837	2.933627	4.377805	2.441116	2.668992	3.16225
Niacin, TM	2.363438	2.858138	1.501664	2.400247	4.61596	4.357631	0.259319	0.257617	0.251158	1.747024	1.302653	0.631874	1.020797	1.048443	1.665524	0.228727	0.286822	0.360698
Acetamide	2.150765	1.886858	2.017471	1.436689	2.943267	2.681093	1.854133	1.290115	1.21038	1.563492	2.267828	1.604658	1.537215	1.662787	1.939838	0.925119	1.3	1.069813
L-Tryptoph	0.171158	0.445605	0.099002	0	0.130299	0.205011	0.118314	0.085647	0	1.430556	1.943615	0.166105	0.17338	0.294747	0.117519	0.663036	0.110078	0.188106
Serine, 3TN	46.59578	74.56919	26.90516	30.82891	48.01496	39.06606	28.12318	12.07041	36.35959	98.43651	76.23881	16.48934	16.09282	31.05379	16.03491	47.36828	57.11008	42.13575
.alpha.-D-(	0.165331	0.326371	1.02787	0.302656	0.283666	1.987472	0.930308	0.93568	0.616311	0.303571	1.038972	0.969416	1.937609	1.807172	2.805175	0.513274	0.955814	0.286361
D-Glucose,	657.3401	870.6005	930.2895	851.6467	1076.349	1247.636	1164.447	839.063	1301.59	1052.952	1587.37	792.9966	572.1891	726.6889	768	1004.482	1310.561	927.3588
Glyceryl-gl	0.085215	0.124456	0.245424	0.096356	0.11409	0.273918	0.235008	0.170278	0.237257	0.093254	0.162521	0.224467	0.25	0.221768	0.227244	0.142274	0.210078	0.098901
32.1874 mi	0.06555	0	0.073211	0	0.059227	0.082574	0	0.04807	0	0	0.069652	0.034231	0.040937	0.061025	0.062032	0	0.064341	0.045249
.beta.-Gen	0.191551	0.292428	0.18802	0.121062	0.201995	0.223235	0.264182	0.113406	0.179796	0.345238	0.218076	0.140572	0.136821	0.212016	0.169576	0.167461	0.162016	0.12799

Galactinol,	0.036417	0	0.055324	0	0	0.050683	0	0.088693	0	0	0.108624	0.063692	0.065674	0.054105	0.067643	0	0.062016	0
L-(+)-Rham	34.23744	31.27589	11.33111	8.531192	16.79302	20.41913	15.18639	8.398104	8.033364	27.13492	19.97347	12.56566	11.33975	15.05127	8.221945	14.83458	10.30155	14.63478
Arabinofur	0	0	0	0	0	0	0	0	0	0	0	0	0	0	0	0	0	0
.beta.-D-Ga	1.72469	1.97215	4.356489	2.200741	4.405237	6.339408	2.596434	3.519296	3.722892	2.069444	7.072968	4.584175	2.738616	3.737653	5.194514	2.610619	3.551938	2.844215
L-Tyrosine,	133.7334	188.7694	64.31947	20.04694	115.591	116.5923	61.09562	32.51726	49.66821	212.8254	171.0912	40.76319	33.77933	65.36898	39.87032	97.41593	115.3488	90.2702
3-Hydroxy-	0.046613	0.057441	0.034526	0.03706	0.054239	0.048975	0	0.032837	0	0.069444	0.072139	0.023008	0.025613	0.033658	0.020885	0.039483	0	0.043956
L-Phenylala	127.4873	144.5988	59.63394	51.55775	95.12219	85.97722	75.07293	36.2329	79.7924	179.4921	129.4328	36.39282	29.8704	49.48474	38.55112	74.20558	91.2062	82.21849
Cysteine, 3	0.236708	0.26893	0.15807	0.151946	0.279925	0.328018	0.08752	0.066012	0.159407	0.400794	0.258706	0.089226	0.092382	0.133375	0.182045	0.19469	0.241085	0.243697
L-Glutamic	8.233795	7.671018	5.014559	2.575664	10.41397	7.036446	5.711507	3.076168	5.417053	7.358135	6.94859	2.170314	2.300131	3.235923	2.899314	4.973451	6.989922	5.544279
2,6-Bis(tert	1.407138	1.517842	0.891015	1.068561	1.140898	1.055809	0.946515	0.681787	1.439296	1.964286	1.728856	0.590629	0.567207	0.679459	0.69919	1.316542	1.04186	1.296703
Adenine, 2	0.053897	0.087032	0.023295	0	0.043017	0.046697	0	0.040284	0	0	0	0	0	0	0	0	0.039535	0
2-Aminohe	20.25929	23.08094	34.90183	19.10315	36.33416	39.02506	23.56564	26.19093	13.07692	26.36508	36.73632	30.211	25.28722	27.74457	23.27681	23.27025	18.45581	24.61021
Benzene-1,	0	0.074848	0.500416	0.143298	0.104738	0.524487	0.447326	0.358835	0.274328	0	0.277778	0.813412	0.597636	0.415854	0.605985	0.450647	0.471318	0.148028
Pyridoxine,	2.612527	2.990426	1.371464	1.23286	2.71384	2.431663	1.353323	1.023358	1.358665	3.270833	3.195688	1.049383	1.255254	1.603964	1.312032	1.82437	2.536434	2.179703
Pentasiloxa	2.517116	3.00087	1.278702	1.09265	2.180175	1.529613	2.126418	0.925863	1.848934	4.036706	2.529851	0.816498	0.677977	0.954703	0.682357	1.786249	2.173643	2.195863
11.8062 mi	32.75164	43.39077	36.45923	27.56269	40.58853	31.34852	33.99676	28.83412	44.59685	34.86508	37.67164	16.64422	20.77058	16.85561	23.06234	33.68822	28.45891	40.84809
22.4512 mi	16.42535	20.03829	9.324459	8.818406	14.75312	14.94305	11.23987	6.618822	12.15941	24.03175	19.46269	7.243547	6.369527	8.410192	6.900249	11.71409	15.53488	12.45766
Galactose (	1.884195	2.975631	5.595258	3.651019	5.351621	6.797836	5.748784	5.451591	6.750695	3.058532	8.063018	5.515152	3.914186	4.284052	4.975374	4.415929	5.956589	4.159017
Mannose (	0.94319	1.138381	0.668469	0.307597	1.256234	0.764806	1.055105	0.593094	0.991659	1.637897	1.635987	0.508418	0.501313	0.658383	0.476309	0.933969	1.135659	1.387201
Mannitol	1.707939	1.905135	0.670965	0.54231	1.232544	1.17369	0.401945	0.406567	0.721038	2.230159	1.995854	0.597924	0.585377	0.934571	0.509975	0.752893	1.107752	0.868132
19.2884 mi	2.005098	2.64839	0.761647	0.54231	1.586658	1.238041	0.492707	0.406567	0.721038	3.292659	2.800166	0.823513	0.785683	1.196603	0.684539	0.945541	1.222481	1.199095
3,4-Dihydr	0.241078	0.704961	0.499168	0.720815	0.652743	0.741458	0.298217	0.246107	0.437442	0.709325	0.957711	0.178451	0.328371	0.507707	0.351309	0.283186	0.368992	0.363284
Sucrose	0	0	0	0	0	0	0	0	0	0	0	0	0.02014	0.030198	0.01995	0	0	0
32.7625 mi	0.375091	0.326371	0.180116	0.161211	0.282419	0.25	0.230146	0.186188	0.358665	0.088294	0.058043	0.047138	0.037434	0.035231	0.045511	0	0	0
33.2715 mi	0.12673	0.113142	0.081115	0.050031	0.065461	0.10877	0	0.048747	0	0.073413	0.115257	0.031425	0.062391	0.060711	0.055175	0.063308	0.075194	0.130575

Values represent normalized GC-MS metabolite responses for all detected metabolites across biological replicates. 'All' indicates mixed cultures containing *T. vaginalis* and all three BV-associated bacteria; 'Tv' indicates axenic *T. vaginalis* cultures. 'M' and 'T' indicate MCF and TMS derivatization datasets, respectively. Numbers 8, 16 and 32 indicate incubation timepoints in hours. Letters a–c indicate biological replicates.
