# Supplementary material for: Metabolic cooperation supports mutual growth between the protozoan parasite Trichomonas vaginalis and dysbiotic vaginal bacteria
Source: PLoS Pathog. 2026 Sep 23;22(9):e1014620. doi: 10.1371/journal.ppat.1014620 (PMC13626468; doi:10.1371/journal.ppat.1014620)
Supplement: Supplementary file 8 [file ppat.1014620.s009.pdf]

S1 (A)

Neo1/Neo2

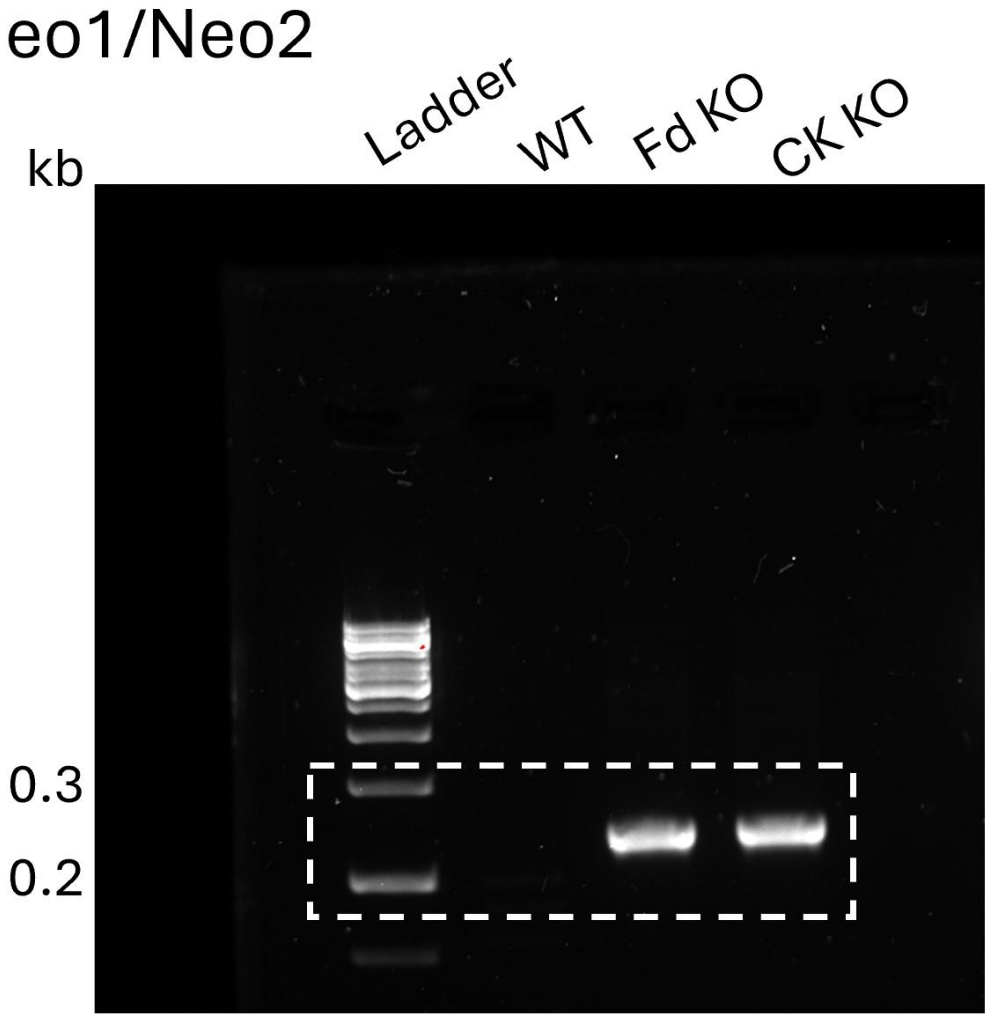

Fd_GOI1/Neo2

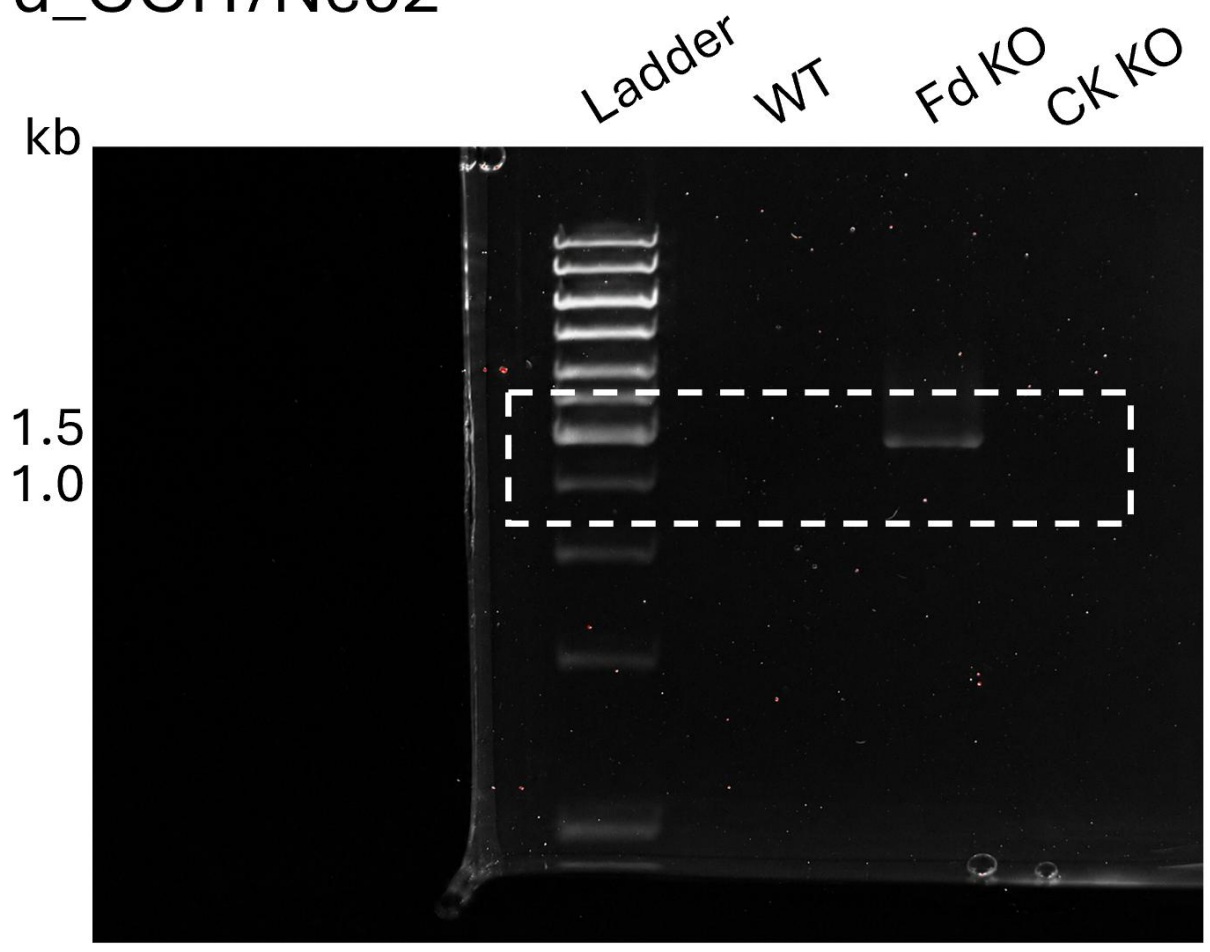

CK_GOI1/Neo2

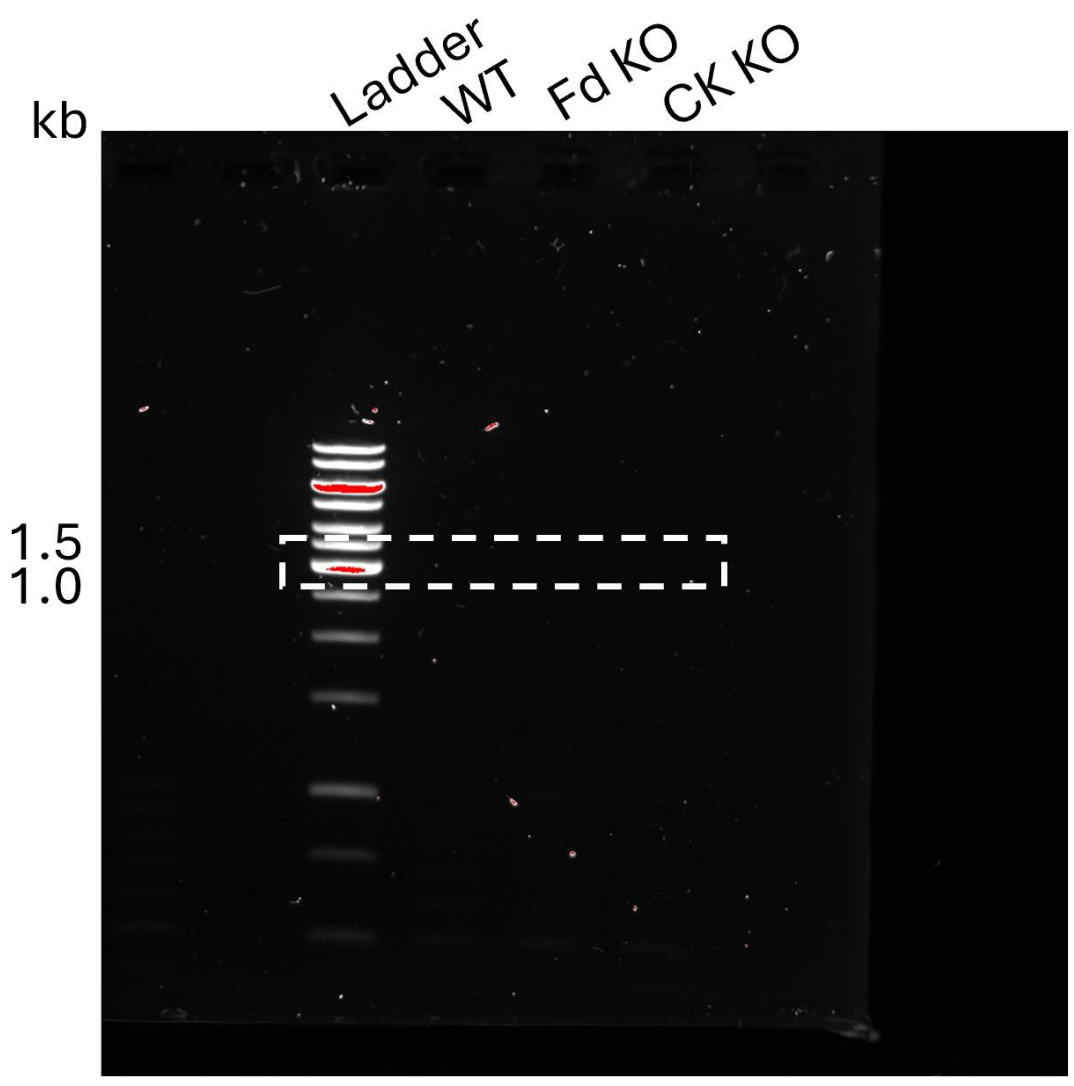

Fd GOI1/GOI2

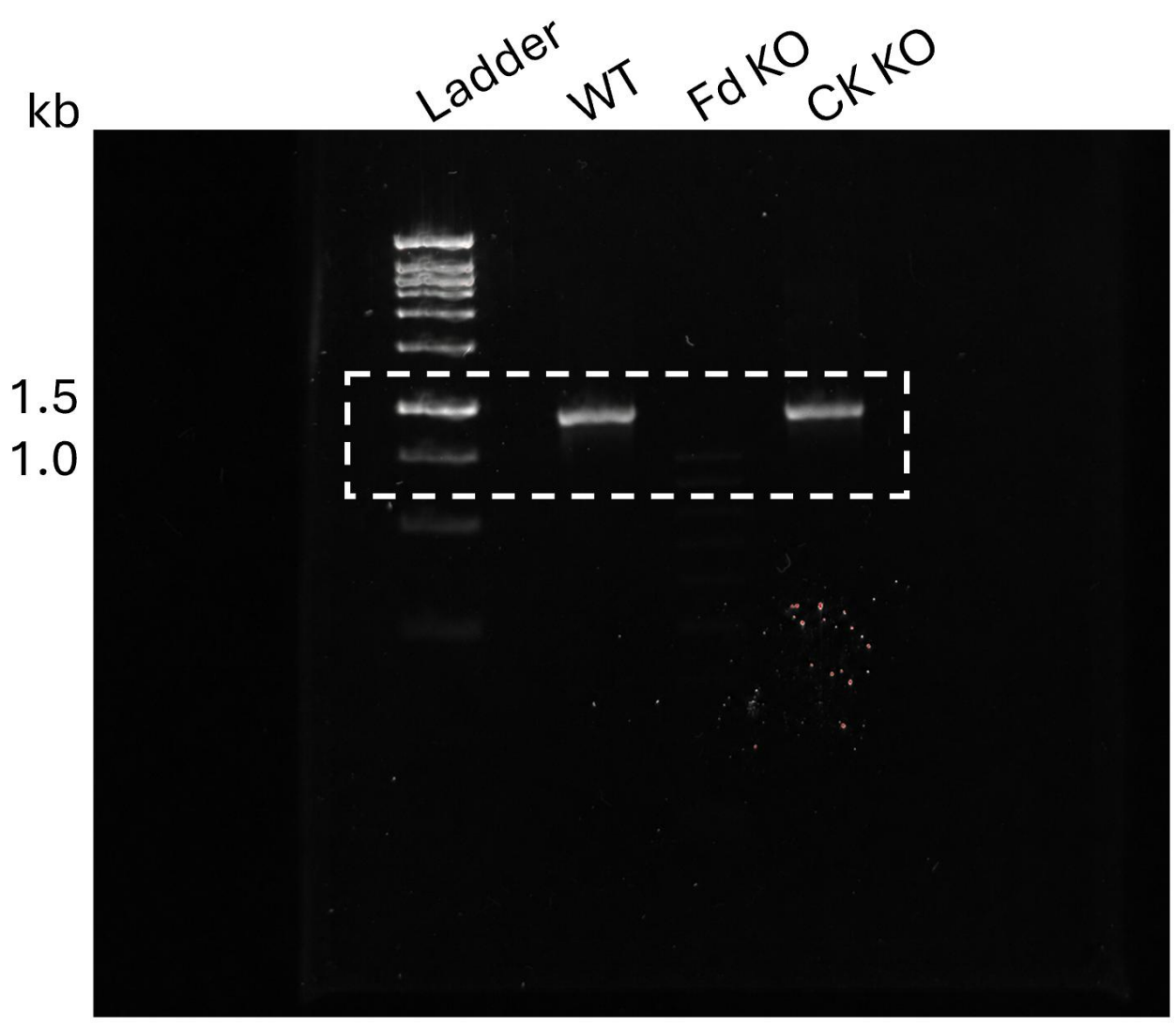

CK GOI1/GOI2

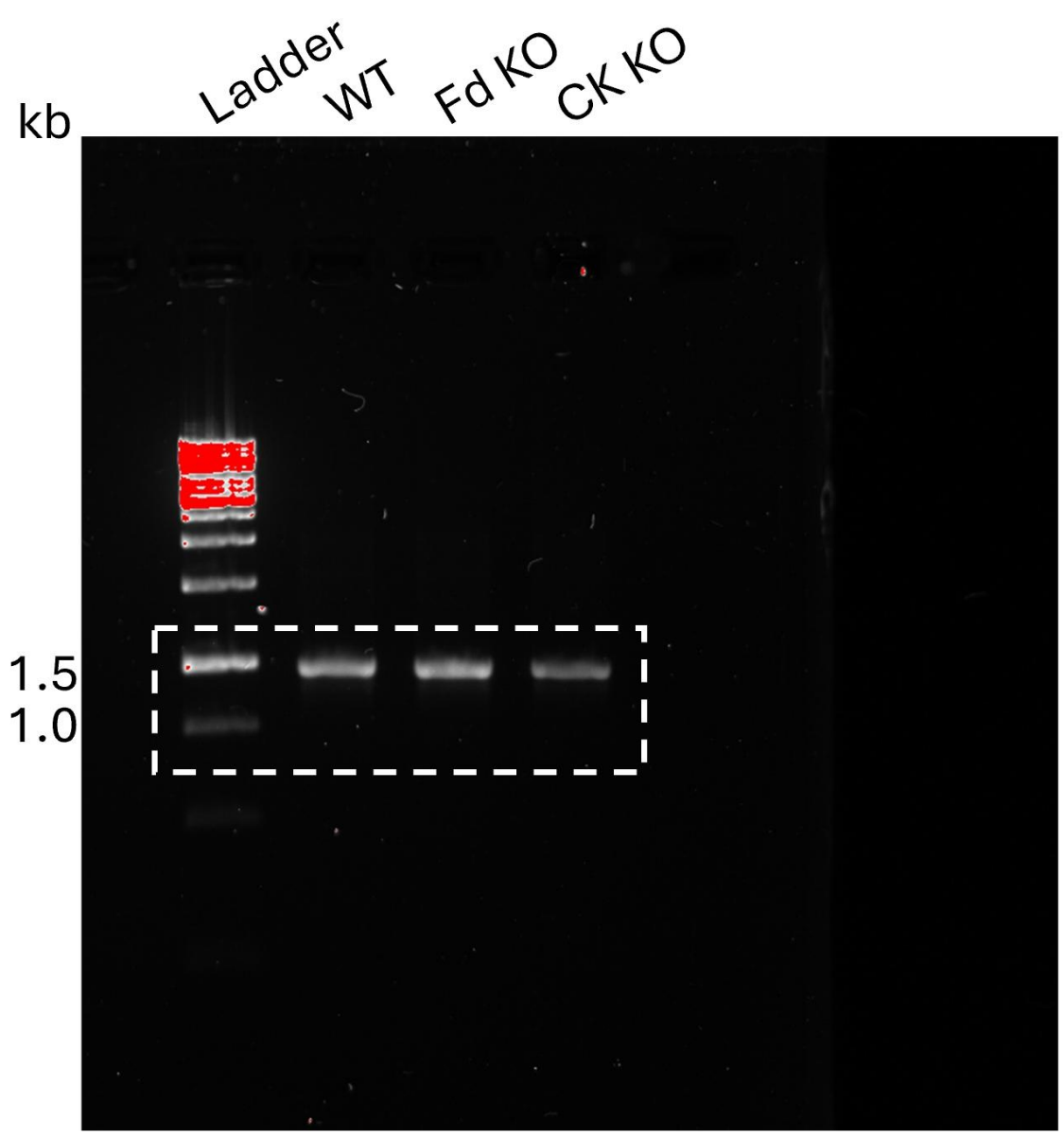

S1 Raw Images. (A) Un-cropped agarose get images related to Figure 8A. Dotted boxes indicate cropped portion used in respective figures.

S1 (B)

Mycoplasma hominis

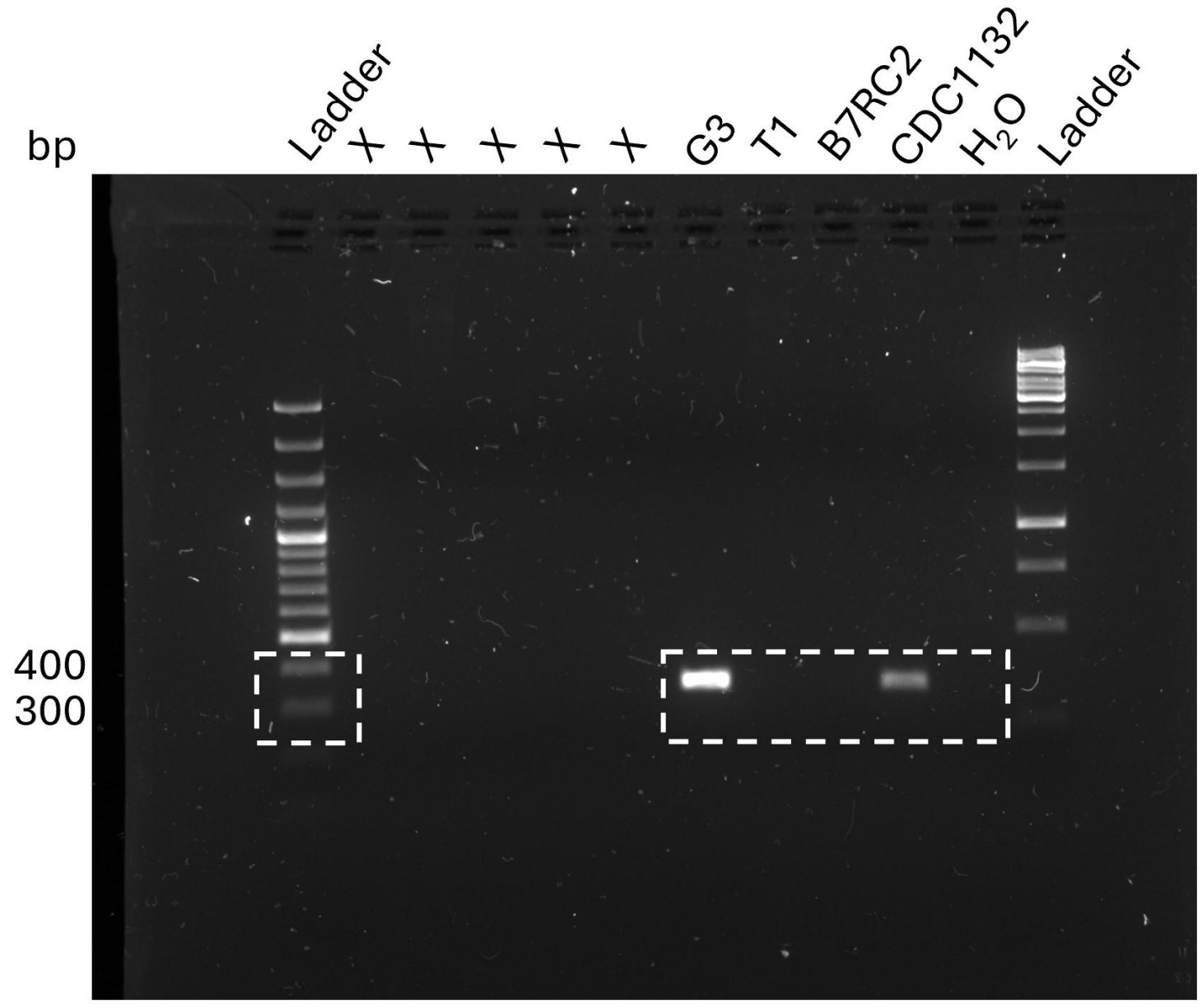

Candidatus Mycoplasma girerdii

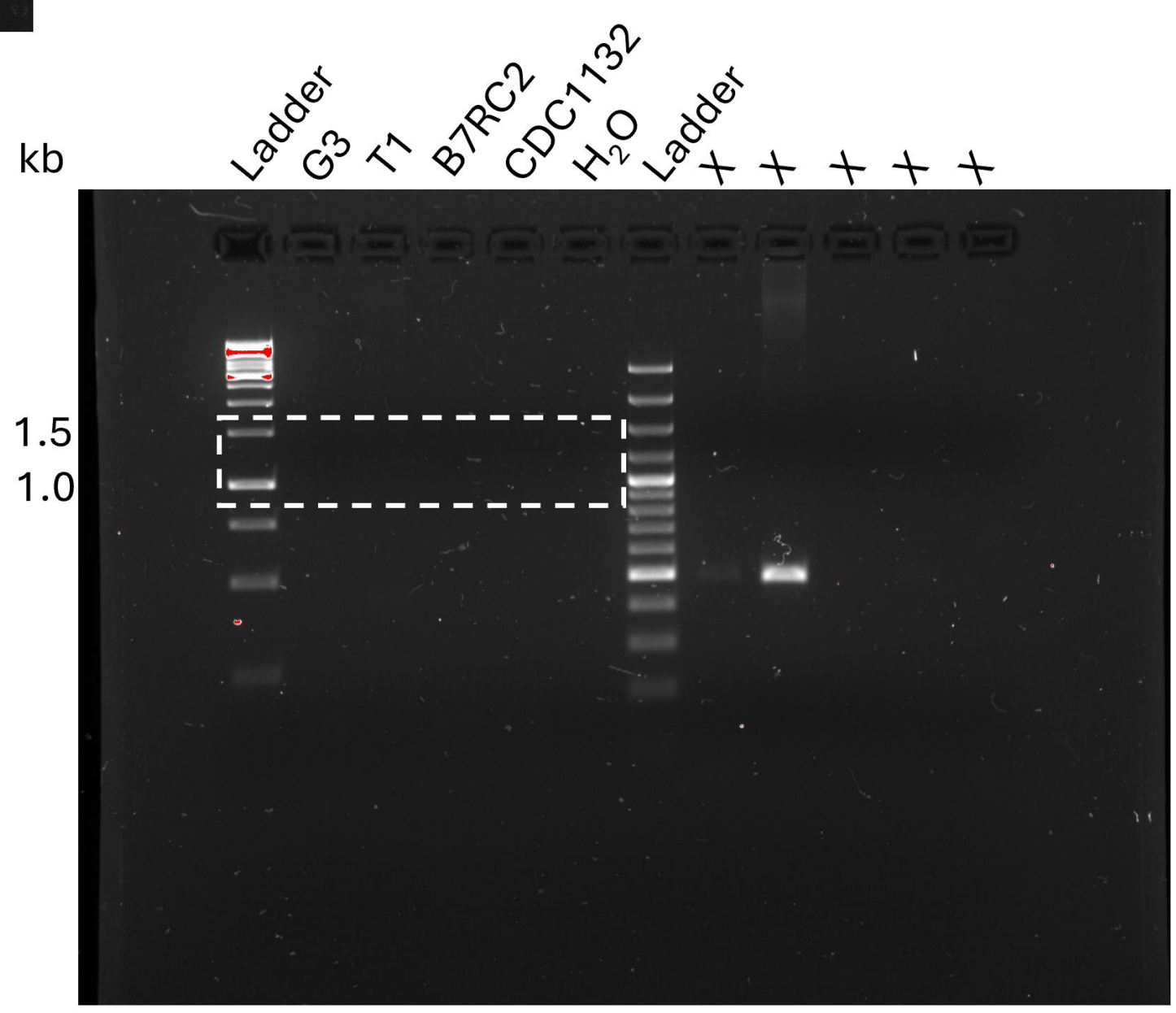

Trichomonas vaginalis

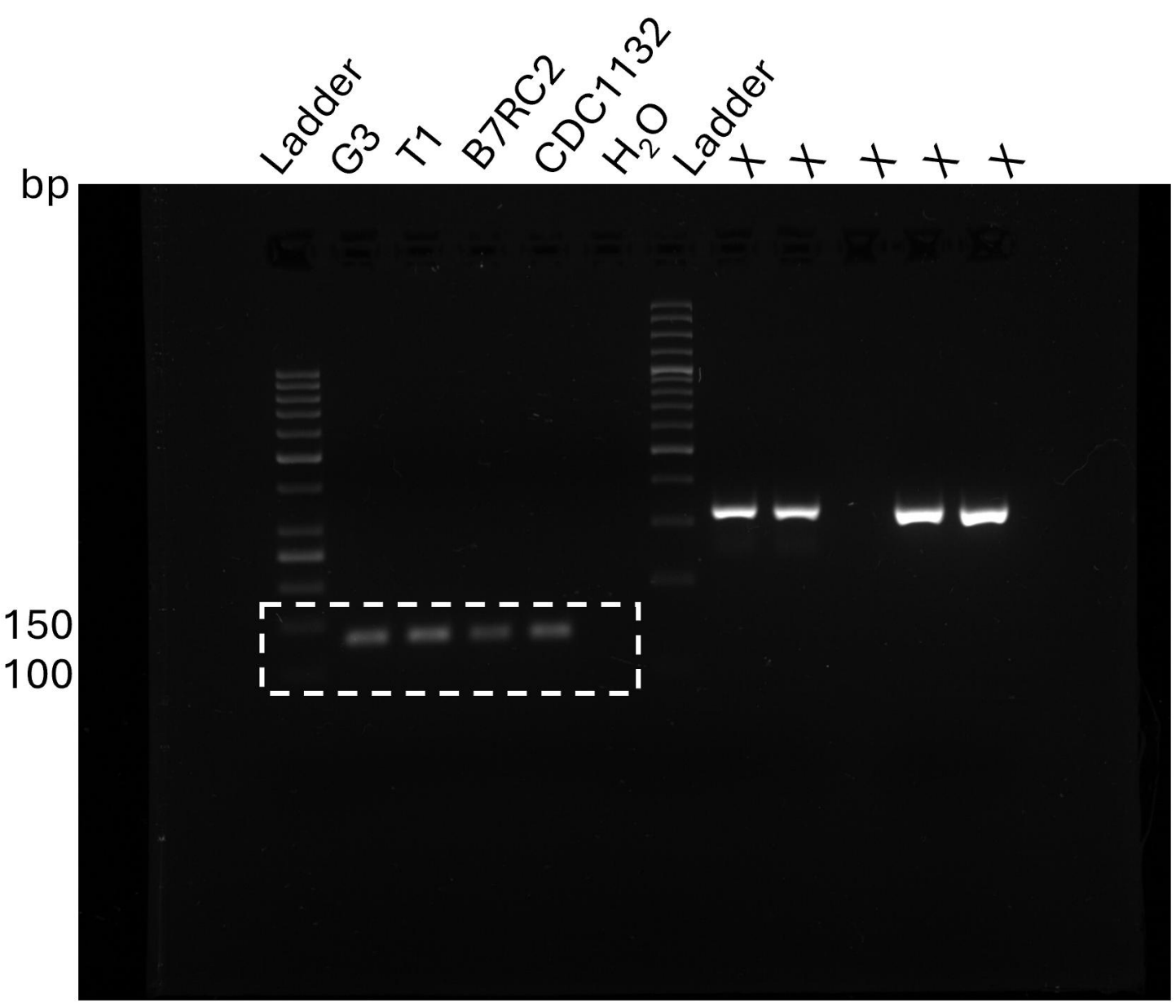

S1 Raw Images. (B) Un-cropped agarose get images related to S1 Fig. Dotted boxes indicate cropped portion used in respective figures.
