# Supplementary material for: Metabolic cooperation supports mutual growth between the protozoan parasite Trichomonas vaginalis and dysbiotic vaginal bacteria
Source: PLoS Pathog. 2026 Sep 23;22(9):e1014620. doi: 10.1371/journal.ppat.1014620 (PMC13626468; doi:10.1371/journal.ppat.1014620)
Supplement: Supplementary file 9 [file ppat.1014620.s011.docx]

Dear Editor and Reviewers,

We thank the Editor and the reviewers for their careful evaluation of our manuscript and for the constructive comments. We are pleased that the reviewers found the study interesting, novel, and relevant to the field. We have revised the manuscript carefully and have performed additional experiments and analyses to address the major points raised. We believe these revisions have substantially strengthened the manuscript:

1. PCR screening for *Mycoplasma hominis* and *Mycoplasma girerdii* in the *T. vaginalis* strain used in this study.
2. Additional analysis of DFMO-treated culture supernatants by GC-MS to further evaluate the ADH/polyamine metabolic axis.
3. Expanded methodological descriptions, including parasite viability assessment, staining procedures, inoculum ratios, pH conditions, and statistical analyses.
4. Additional data presentation improvements, improved figure legends, p-values, error bar definitions, and figure formatting.
5. Expanded discussion of study limitations and pathobiological relevance, including host-cell models, in vivo validation, strain diversity, and the ecological implications of metabolic cooperation.

**EDITOR**

**Editor comment**, quote: “All of the readers suggested several places for improvement but the overall recommendation was positive. Please address the concerns, particularly those of reviewer #2, which may require a few more control experiments and important clarifications.”

**Response.** We thank the Editor for the constructive assessment and for the opportunity to revise our manuscript. We have paid particular attention to Reviewer #2’s comments and have addressed them by adding new experimental controls, expanding the Materials and Methods, improving figure presentation, and clarifying the interpretation of our findings. These changes are described in detail below.

**JOURNAL REQUIREMENTS**

**Requirement 1: CRediT author contributions**

**Response.** We have updated the CRediT author contribution information in the submission system to ensure that all authors’ contributions are complete and accurate.

**Requirement 2: Manuscript source file**

**Response.** We have provided the revised manuscript as a DOCX source file as requested.

**Requirement 3: Author Summary**

**Response.** An Author Summary of 207 words has been included between the Abstract and Introduction from the original submission.

**Requirement 4: Trademark/copyright symbols**

**Response.** We have removed all trademark/copyright symbols from proprietary names throughout the manuscript.

**REVIEWER #1**

We thank Reviewer #1 for the positive assessment of our manuscript and for recognizing the importance of cross-kingdom metabolic cooperation between *T. vaginalis* and bacterial vaginosis-associated bacteria.

**Reviewer #1 - General comment: Pathogenicity and infection-related framing**

**Comment.** The reviewer suggested that pathogenicity and infection-related processes could be frame more explicitly rather than simply mutual microbial growth.

**Response.** We agree with the reviewer of broad implications beyond metabolic microbial cooperation. We have added a couple of final sentences, one in the Introduction and one in the Discussion, to emphasize this point: [lines 131-134 and 382-385].

**MAJOR comment: Study limitations.** The reviewer noted that limitations should be more clearly acknowledged, including the absence of host-cell co-culture systems, in vivo experiments, multiple *T. vaginalis* isolates, and possible contributions of *Mycoplasma* endosymbionts.

**Response.** We agree that these limitations could be explicitly acknowledged. We also agree that *Mycoplasma* endosymbionts, in particular, are important potential confounders in studies of *T. vaginalis* metabolism and pathobiology. We performed PCR screening for *M. hominis* and “*Candidatus Mycoplasma girerdii*” in the working cultures (G3 and others). We did the following changes:

- We described the PCR detection in Methods with references [lines 518-521].
- We presented the results of the PCR screening in Results [lines 198-199] (S1 Fig).
- We added a couple of sentences to acknowledge the potential impact of mycoplasma in Discussion [lines 447-449 and 473-479], and replaced the original sentence “However, these experiments were performed in a simplified co-culture system that does not reproduce the full complexity of the vaginal environment and should therefore be interpreted with this limitation in mind” by expanding these limitations as a full paragraph [lines 488-494].

**MAJOR Comment**. Resolution of figures and tables.

**Response.** The original figure files were prepared according to the journal’s requirements. We recognize that figures embedded in the review PDF may appear compressed relative to the uploaded high-resolution files. For the revised submission, all figures have been rechecked before resubmission.

**MINOR comments**

**Comment**. Lines 80-81 require a reference

**Response.** We agree that this statement should be explicitly supported. Although the statement was based on the studies cited in the preceding sentence, we have repeated the relevant references at the end of the sentence for clarity.

**Comment**. Lines 137-138 are too vague

**Response.** We agree that the original wording was too broad, and we are glad the reviewers spotted it. Our intention was to explain the rationale for testing co-cultivation under a single medium, rather than to imply that these organisms have identical nutritional requirements. We have modified this section accordingly at the first two paragraphs of Results.

**Comment.** Figure 7C could not be identified

**Response.** We thank the reviewer for identifying this error. We have corrected the text and figure legend to refer correctly to Figure 7B.

**Comment.** Typographical error “aammonia”

**Response.** This typographical error has been corrected.

**Comment.** Mycoplasma and dsRNA virus status of G3

**Response.** We believe that we have now clarified this point, as described under the Reviewer’s Major Comment. We have also clarified that the dsRNA virus/TVV status of our working stock was not determined in this study, but added references stating the expected TVV-status of G3 [lines 521-522].

**Comment.** Why bacterial counts were not assessed as CFU

**Response.** In the original experiments, we used spot plating as a qualitative readout because full CFU enumeration across multiple time points, culture conditions, biological replicates, and anaerobic bacterial species would have required extensive serial dilutions and large numbers of agar plates maintained strictly under anaerobic conditions. Importantly, this qualitative spot-plating approach has been used and validated in our previous studies of *T. vaginalis-*bacteria interactions by our group (Pinheiro et al. *mBio*. 2018. doi:10.1128/mBio.01784-18 and Barnett et al. *PLoS Pathog* 2023. doi:10.1371/journal.ppat.1011563) where it was shown to provide a reliable visual assessment of relative bacterial survival/growth compared to quantitative plating approaches. However, we thought it was pertinent to briefly clarify that in Methods [lines 548-549].

**Comment.** Protozoan viability.

**Response.** We thank the reviewer for pointing out that the protozoan viability assessment was not sufficiently clear. In the original manuscript, we stated that *T. vaginalis* viability was assessed using the TC20 automated cell counter, but we agree that it was not clear that this applied to both axenic and co-culture samples. We have now revised the Methods to clarify that parasite viability was assessed by the TC20 automated cell counter “during cultivation both axenically and in co-cultures with bacteria” [lines 516-517]. Because *T. vaginalis* cells are substantially larger than bacterial cells, bacteria were well below the relevant imaging/size-detection range and do not interfere with parasite viability scoring via trypan blue exclusion using the TC20 automated cell counter (Bio-Rad).

**Comment.** Why gene expression was measured only at 8 h.

**Response.** The 8 h timepoint was selected to capture an early transcriptional response of *T. vaginalis* to BVAB before later differences in microbial abundance, nutrient depletion, and accumulated extracellular metabolites become more pronounced. This choice was also guided by our previous studies showing that *T. vaginalis* responds to bacterial co-incubation within early interaction windows through changes in bacteria-responsive genes and proteins (Pinheiro et al. *mBio*. 2018. doi:10.1128/mBio.01784-18 and Barnett et al. *PLoS Pathog* 2023. doi:10.1371/journal.ppat.1011563). In the original submission, we have acknowledged that time-resolved dual RNA-seq or meta-transcriptomic analysis would be valuable in future studies to define coordinated parasite and bacterial transcriptional responses across the full co-culture time course.

**REVIEWER #2**

We thank Reviewer #2 for the detailed and constructive critique and for recognizing the strengths of the study, including the use of multiple BV-associated bacterial species, time-course metabolomics, and the attempt to genetically interrogate *Tv_CK1*. We have paid particular attention to this reviewer’s comments, and have addressed the major concerns through additional experiments, expanded methods, and improved data presentation. Because several comments raised by Reviewer #2 concern related issues, we address some of them collectively below to avoid repetition.

**MAJOR comment 1:** *Mycoplasma*

**Comment.** The reviewer noted that the status of *M. hominis* and *M. girerdii* in the *T. vaginalis* strain used was not indicated and requested PCR testing.

**Response.** We have now performed PCR screening for *M. hominis* and “*Candidatus Mycoplasma girerdii*” in the working cultures (G3 and others). We did the following changes:

- We described the PCR detection in Methods with references [lines 518-521].
- We presented the results of the PCR screening in Results [lines 198-199] (S1 Fig)
- We added a couple of sentences to acknowledge the potential impact of mycoplasma in the Discussion [lines 447-449 and 473-479].

**MAJOR comment 2 and MINOR Comments 6 and 8:** Protozoan viability, staining, and flow cytometry gating

**Comment.** The reviewer requested clarification on how parasite viability was assessed, how cells were stained, and requested a flow cytometry gating strategy with appropriate controls.

**Response.** We thank the reviewer for noting that our original Methods did not clearly distinguish between protozoan viability and enumeration. We have revised the Methods to clarify that *T. vaginalis* viability was assessed by the TC20 automated cell counter, whereas flow cytometry was used to enumerate fluorescently labelled *T. vaginalis* cells in axenic and co-culture samples. Because *T. vaginalis* cells are substantially larger than the bacterial cells present in co-culture, bacteria do not interfere with TC20-based parasite viability assessment. We have also added methodological details on the flow cytometry enumeration approach. To make these changes, this paragraph was substantially modified [lines 533-551]. This staining procedure and gating strategy was previously optimized and validated in a dedicated methods paper from our group and was subsequently applied in two of our previous *T. vaginalis*-bacteria co-culture studies. These three references are now explicitly stated in the revised Methods.

**MAJOR Comments 3 and 5; MINOR Comment 7:** Bacterial inocula and bacterial growth quantification

**Comment.** Here, the reviewer wanted **(i)** clarification of bacterial inocula, whether bacteria were added as total bacteria or per species, and **(ii)** quantification of bacterial growth, including total CFU in the three-species BVAB cultures and *G. vaginalis* quantification in Fig 8.

**Responses. (i)** We agree that this point could be clarified. In the original manuscript, we stated in the Materials and Methods that *T. vaginalis* and bacteria were mixed at a 1:10 protozoan:bacterial ratio, and the relevant figure legends indicated the initial protozoan inoculum. However, we appreciate that this wording did not explicitly state whether the bacterial inoculum referred to total bacteria or to each bacterial species. We have now clarified in the Methods that “When multiple bacterial species were used, the total bacterial inoculum represented the combined population divided equally among the indicated species” [lines 538-540]. Since protozoal inoculum is stated in every figure legend, where appropriate, we hope this would clarify. For example, an initial inoculum of 2 X 10⁴ protozoa/mL means that the corresponding total bacterial inoculum was 2 X 10⁵ bacteria/mL, composed of the indicated bacterial species. **(ii)** CFU enumeration across multiple timepoints, biological replicates, culture conditions, and bacterial species would have required extensive serial dilutions and large numbers of agar plates under anaerobic conditions and, as the reviewer acknowledged, it would not distinguish bacterial species on the non-selective agar. The qualitative spot-plating approach, used here, has been validated in our previous studies of *T. vaginalis*-bacteria interactions shown to provide a reliable visual assessment of relative bacterial survival/growth as compared to quantitative CFU (Pinheiro et al., mBio 2018; Barnett et al., PLOS Pathogens 2023). We have now clarified this point in the revised Methods [lines 548-549]. We also agree that quantitative bacterial enumeration would provide additional information in Fig 8. However, the bacterial data in this experiment were originally generated as a qualitative companion readout to *T. vaginalis* growth response to DFMO. To avoid overinterpretation, we have noted “as qualitative readout for bacterial growth” in the legend of Fig 8.

**MAJOR Comment 4:** Mass spec support of ADH pathway in co-culture

**Comment.** The reviewer requested an assay to assess ATP production during co-culture to support the metabolomics data and increased utilization of the ADH pathway.

**Response.** We agree with the reviewer that an additional functional readout of the metabolic pathway would strengthen the interpretation of increased ADH/polyamine-axis activity during co-culture. We carefully considered measuring total ATP in the mixed cultures. However, we concluded that total ATP measurements in this system would be difficult to interpret because ATP would reflect the combined contribution of multiple organisms in the culture and would also be strongly influenced by differences in microbial abundance. This is particularly relevant for mixed cultures, where ATP could not be assigned specifically to *T. vaginalis* or to the bacterial partner(s) and/or its endosymbiont. To address the reviewer’s point more directly, we performed an additional GC-MS analysis of culture supernatants collected from the DFMO experiment, allowing us to determine whether pharmacological inhibition of polyamine biosynthesis altered metabolites within the same pathway as highlighted by the original metabolomics analysis. Consistent with the proposed involvement of the ornithine-putrescine axis, DFMO treatment reduced the abundance of both metabolites. Because DFMO inhibits ornithine decarboxylase and therefore directly targets the polyamine branch downstream of ornithine rather than CK-mediated ATP generation, these data might provide a more direct validation of the pathway branch that was pharmacologically perturbed in our study. These new data are now included as the final part of Fig. 8 (Fig 8D) and described in the Results [lines 358-363]. We have also revised the text to avoid overinterpreting ATP production as directly measured in this study. Instead, we now present the ADH pathway as a plausible energetic axis supported by metabolite accumulation, targeted gene expression analysis, and pharmacological perturbation of the linked polyamine pathway following the changes below:

Results, [lines 243-245]: “The ADH pathway breaks down carbamoyl-phosphate to produce ATP and suggesting that this pathway may be intently utilized to provide additional energy for the microbial co-growth” was replaced by “The ADH pathway can generate ATP through carbamate kinase, suggesting that enhanced arginine metabolism may contribute to the energetic adaptation of co-cultures under nutrient-restricted conditions”.

Results, [lines 336-337]: “These observations suggest ATP and polyamines are key to support the joint metabolism and co-growth of *T. vaginalis* and BVAB in this culture condition” was replaced by “These observations suggest that ADH-linked metabolism and polyamine synthesis contribute to the joint metabolism and co-growth of *T. vaginalis* and BVAB in mixed cultures”.

Discussion [lines 432-435]: “While the contribution of CK-mediated ATP generation remains solely inferred from pathway structure and Tv_CK1 transcriptional upregulation, the reduction of putrescine following DFMO treatment further supports the functional involvement of the ornithine-putrescine branch in this microbial interaction.”

**MINOR Comment 1:** Abstract wording

**Response.** We are glad the reviewer spotted the missing verb in this sentence. We have corrected the incomplete sentence in the Abstract. It now reads: “Inhibition of polyamine biosynthesis partially impaired the cooperative growth of both microorganisms, further supporting the importance of this metabolic axis.”

**MINOR Comment 2:** “Despite increasing awareness”

**Response.** We agree that the phrase “Despite increasing awareness” was unnecessary and made the sentence less clear. We have revised the sentence as suggested. It now reads: “Historical precedent from studies with the protozoan parasite *Entamoeba histolytica* shows that the impact of bacteria in parasite biology and pathogenicity has been recognized for more than a century.”

**MINOR Comment 3:** “spot platting”

**Response.** We have corrected “spot platting” to “spot plating” throughout the manuscript.

**MINOR Comment 4:** Fig 1 reference missing

**Response.** We are glad the reviewer spotted this, and we have included Figure 1 in parentheses with the p-values as requested.

**MINOR Comment 5:** DMEM pH

**Response.** We agree with reviewer. There is also a wide variation of pH in TYM media as well, as reported in literature for *T. vaginalis*, anywhere from 5.8 to 7.2. We now disclose the pH for both TYM and DMEM, as used for our experiments (i.e. pH 6.2 and 7.2, respectively), in Materials and Methods.

**MINOR Comment 9**: ‘highly viable’, Fig 2.

**Response.** We agree that ‘highly viable’ was imprecise and unnecessary because viability is explicitly shown in Fig. 2. We have removed this wording from the figure legend.

**MINOR Comment 10:** error bars, what bars represent and statistical tests to fig legends

**Response.** We agree and have revised methods and the figure legends to define what bars, points, error bars, and box plots represent. For quantitative bar and curve plots, the legends now state that data are shown as mean +/- standard deviation (SD), indicate the number of independent biological replicates, and specify the statistical tests used. For Figure 6, we clarified that box plots show the median and interquartile range, with whiskers extending to the most extreme non-outlier values within 1.5 X the interquartile range. We also added or clarified p-value annotations where appropriate.

**MINOR Comment 11:** mean, not median?

**Response.** We thank the reviewer for identifying this wording error. The use of “median” was incorrect; the statistical analysis was performed on mean *T. vaginalis* cell numbers, consistent with ANOVA. We have corrected this in the Materials and Methods and revised the relevant figure legends to state that data are shown as mean +/- SD, with the statistical tests specified in each legend.

**MINOR Comment 12:** values, symbols, p-values in Fig 3.

We agree that the statistical comparisons in Figure 3 should be clearer. However, because all conditions differed significantly from the no-bacteria control, we have added a compact annotation directly to the graph stating that all comparisons versus the no-bacteria control were significant (p < 0.01, one-way ANOVA followed by Dunnett’s multiple-comparisons test). This avoids multiple brackets or symbols that would otherwise pollute/obscure the figure itself.

**MINOR Comment 13:** values, symbols, p-values in Fig 3 which appear blurred

We have revised the figure legends to state what bars and error bars represent and to specify the statistical tests used. We have also added p-values or significance symbols where appropriate, including in Figures 3 and 8. We have reviewed Fig 3 and confirmed that the source figure is of high resolution; the apparent blurring likely resulted from compression in the submission-generated PDF. We will provide the final figure files according to PLOS formatting and resolution requirements.

**MINOR Comment 14.** Fig 4 needing clarification of what the error bars represent.

**Response.** We have also revised the Fig 4 legend to state what the bars and error bars represent and to specify the statistical test used (as per minor comment #10).

**MINOR Comment 15:** ‘highly viable’, Fig 4.

**Response**. We agree with the reviewer that “being” was missing (and we fixed it), and the wording was unclear as we forgot to state that “Protozoal cell viability remained high (> 92%) for both conditions and across time points”. This information has now been added to the Fig. 4 legend.

**MINOR Comment 16:** Fig 6 labels

**Response.** We agree that labelling the two metabolites is a good idea and should improve the readability of Fig 6. We have done this by changing from the single y-axis label ‘Normalized metabolite response’ to ‘Normalized putrescine response’ and ‘Normalized ornithine response’ to the top and bottom panels, respectively.

**MINOR Comment 17:** Figure 7C inconsistency

**Response.** We thank the reviewer for identifying this inconsistency. We have corrected the text and legend to match the final two-panel structure of Figure 7, pointing correctly to Figure 7B.

**MINOR Comment 18:** Fig 8, error bars and p-values

**Response.** As per minor comment #10, Fig 8 legend was revised to state what the bars and error bars represent and to specify the statistical test used. This Fig and legend were modified to accommodate new Fig 8D resulting from new experiments under major comment #4.

**REVIEWER #3**

We thank Reviewer # 3 for the very positive assessment of our manuscript and for recognizing the novelty and significance of the study. We also appreciate the reviewer’s helpful suggestions regarding additional literature, interpretation, and data presentation.

**MAJOR comment.** Possible phagocytosis of BV-associated bacteria

**Comment.** The reviewer noted evidence that *T. vaginalis* can phagocytose some *Lactobacillus* species and asked whether there are relevant data for the three BV-associated bacteria tested. The reviewer suggested whether TEM would be feasible.

**Response.** We agree that phagocytosis is an interesting and relevant possibility. We did not directly test whether the BV-associated bacteria used in this study are internalized by the parasite, and there is no precedent for these bacteria in the literature. Because TEM or other microscopy-based assays of bacterial internalization would require substantial additional experimental work and would not directly resolve metabolite exchange, we consider this beyond the scope of the present revision. However, we thought this point deserved a brief statement to the Discussion [lines 479-481] as an important direction for future work.

**MINOR Comments.** Here, the reviewer:

(i) Suggested clarifying that the three bacteria selected for co-culture experiments represent core/major BV-associated bacteria

**Response.** We agree and have made small revisions in the Introduction and Results to clarify that *G. vaginalis*, *A. vaginae*, and *P. bivia* were selected as representative BV-associated bacteria rather than implying that they encompass the full diversity of BV-associated communities. We modified Introduction, [lines 59-60]: “the overgrowth of anaerobic bacteria, including core BV-associated taxa such as *Gardnerella vaginalis*, *Atopobium vaginae*, and *Prevotella bivia*.” and Results, [line 145]: “*T. vaginalis* and the three selected BVAB - *G. vaginalis*, *A. vaginae*, and *P. bivia”*

(ii) Pointed to a citation error for Bradshaw et al. (2025)

**Response.** We have checked and corrected it.

(iii) Suggested a historical literature on *Trichomonas*-bacteria interactions

**Response.** We thank the reviewer for this useful suggestion. We did try to incorporate a broad historical view in the Introduction of this manuscript, but we missed this seminal work. To fix this, we have added a brief sentence to the Introduction [lines 105-108] citing Jírovec and Petrů (1968), to acknowledge that bacteria-*Trichomonas* interactions have a historical precedent in the trichomoniasis literature as well as in the *Entamoeba* field.

(iv) Suggested incorporating recent literature on Mycoplasma endosymbionts and microbiota-targeting mechanisms in *T. vaginalis* and related trichomonads.

**Response.** We thank the reviewer for this useful suggestion. We have added brief discussion of Margarita et al. (2022) in the Discussion, where we consider the relevance of Mycoplasma endosymbionts to *T. vaginalis* metabolism and pathophysiology. We have also added Hart et al. (2025) to the Introduction to acknowledge recent comparative evidence that microbiota-targeting mechanisms are conserved more broadly across *Trichomonas* species. These additions help place our findings within the wider context of parasite-bacteria interactions without substantially expanding the manuscript.

(v) Pointed to an incomplete sentence in the Abstract.

**Response.** We are glad the reviewer spotted the missing verb in this sentence. We have corrected the incomplete sentence in the Abstract. It now reads: “Inhibition of polyamine biosynthesis partially impaired the cooperative growth of both microorganisms, further supporting the importance of this metabolic axis.”

(vi) Suggested that Supplementary Tables S1 and S2 and Supplementary Data 1 and 2 could include additional information so that thedifferent sections and sub-tables can be interpreted without referring back to the manuscript.

**Response.** We agree with the reviewer that having the supplementary metabolomics files ‘self-contained’ could help readers. We have revised Supplementary Tables S1 and S2 to include explanatory legends defining the sample abbreviations, timepoints, biological replicate labels, derivatization methods, normalized metabolite responses, and fold-change calculations. We have also expanded the legend/key in S2 Data and corrected the timepoint description. S1 Data already contained the full analytical workflow and processing report, but we have added a brief introductory note as a header on the document, clarifying the contents of the file. Finally, since we have incorporated additional MCF-only DFMO metabolomics data in the manuscript (to answer the comment from another reviewer), we added the processing report and revised the file legend so that the original TMS/MCF time-course analyses and the DFMO-treatment analysis are clearly distinguished in S1 Data.

(vii) Pointed to acronym consistency 2HB / 2-HB

**Response.** We have standardized the acronym as 2HB throughout the manuscript.
