# Supplementary material for: Metabolic cooperation supports mutual growth between the protozoan parasite Trichomonas vaginalis and dysbiotic vaginal bacteria
Source: PLoS Pathog. 2026 Sep 23;22(9):e1014620. doi: 10.1371/journal.ppat.1014620 (PMC13626468; doi:10.1371/journal.ppat.1014620)
Supplement: Peer review history

# Peer review: Decision Letter 0

**DOI:** 10.1371/journal.ppat.1014620.r001

**Authors:** Dominique Soldati-Favre, William Navarre

3 May 2026

Metabolic cooperation supports mutual growth between the protozoan parasite Trichomonas vaginalis and dysbiotic vaginal bacteria

PLOS Pathogens

Dear Dr. Simoes-Barbosa,

Thank you for submitting your manuscript to PLOS Pathogens. After careful consideration, we feel that it has merit but does not fully meet PLOS Pathogens's publication criteria as it currently stands. Therefore, we invite you to submit a revised version of the manuscript that addresses the points raised during the review process.

Please submit your revised manuscript by Jul 02 2026 11:59PM. If you will need more time than this to complete your revisions, please reply to this message or contact the journal office at plospathogens@plos.org. When you're ready to submit your revision, log on to https://www.editorialmanager.com/ppathogens/ and select the 'Submissions Needing Revision' folder to locate your manuscript file.

Please include the following items when submitting your revised manuscript:

* A letter that responds to each point raised by the editor and reviewer(s). You should upload this letter as a separate file labeled 'Response to Reviewers'. This file does not need to include responses to any formatting updates and technical items listed in the 'Journal Requirements' section below.

* A marked-up copy of your manuscript that highlights changes made to the original version. You should upload this as a separate file labeled 'Revised Manuscript with Track Changes'.

* An unmarked version of your revised paper without tracked changes. You should upload this as a separate file labeled 'Manuscript'.

If you would like to make changes to your financial disclosure, competing interests statement, or data availability statement, please make these updates within the submission form at the time of resubmission. Guidelines for resubmitting your figure files are available below the reviewer comments at the end of this letter.

We look forward to receiving your revised manuscript.

Kind regards,

William Navarre

Academic Editor

PLOS Pathogens

Dominique Soldati-Favre

Section Editor

PLOS Pathogens

Sumita Bhaduri-McIntosh

Editor-in-Chief

PLOS Pathogens

orcid.org/0000-0003-2946-9497

Michael Malim

PLOS Pathogens

orcid.org/0000-0002-7699-2064

**Additional Editor Comments:**

Your manuscript was reviewed by three experts in the field. All of the readers suggested several places for improvement but the overall recommendation was positive. Please address the concerns, particularly those of reviewer #2, which may require a few more control experiments and important clarifications.

**Journal Requirements:**

If the reviewer comments include a recommendation to cite specific previously published works, please review and evaluate these publications to determine whether they are relevant and should be cited. There is no requirement to cite these works unless the editor has indicated otherwise.

1) Please ensure that the CRediT author contributions listed for every co-author are completed accurately and in full.

At this stage, the following Authors/Authors require contributions: Augusto Simoes-Barbosa. Please ensure that the full contributions of each author are acknowledged in the "Add/Edit/Remove Authors" section of our submission form.

The list of CRediT author contributions may be found here: https://journals.plos.org/plospathogens/s/authorship#loc-author-contributions

2) We ask that a manuscript source file is provided at Revision. Please upload your manuscript file as a .doc, .docx, .rtf or .tex. If you are providing a .tex file, please upload it under the item type u2018LaTeX Source Fileu2019 and leave your .pdf version as the item type u2018Manuscriptu2019.

3) Please provide an Author Summary. This should appear in your manuscript between the Abstract (if applicable) and the Introduction, and should be 150-200 words long. The aim should be to make your findings accessible to a wide audience that includes both scientists and non-scientists. Sample summaries can be found on our website under Submission Guidelines:

https://journals.plos.org/plospathogens/s/submission-guidelines#loc-parts-of-a-submission

4) We do not publish any copyright or trademark symbols that usually accompany proprietary names, eg ©,  ®, or TM  (e.g. next to drug or reagent names). Therefore please remove all instances of trademark/copyright symbols throughout the text, including:

- TM on pages: 25, and 26.

**Reviewers' Comments:**

Reviewer's Responses to Questions

**Part I - Summary**

Please use this section to discuss strengths/weaknesses of study, novelty/significance, general execution and scholarship.

Reviewer #1: This is an interesting manuscript addressing a particularly complex and fascinating topic, namely the interactions between different pathogens. The results presented suggest that cross-kingdom metabolic cooperation between the flagellated protozoan T. vaginalis and selected bacterial species associated with bacterial vaginosis may actively contribute to both infection and dysbiosis. In particular, the metabolic activities related to energy production and polyamine biosynthesis appear to play an important role.

The manuscript is well written, and the experiments are clearly described.

However, in my opinion, the authors should improve the presentation of the results by framing them more explicitly in terms of modulation of pathogenicity and infection-related processes, in order to make the manuscript more aligned with the scope of the journal. Although the biochemical and metabolic aspects are presented appropriately, greater emphasis should be placed on how these microbial interactions may influence the progression and outcome of infection, rather than simply supporting mutual microbial growth or increasing ATP production.

Reviewer #2: The authors of this manuscript attempt to address an interesting and understudied aspect of T. vaginalis infection, its association with other microbes present during BV. The manuscript focuses on growth of T. vaginalis when co-cultures with three bacterial species associated with BV under nutrient deplete conditions. The authors show that both parasite and bacteria can grow under nutrient deplete conditions when grown in co-culture. The authors characterize metabolic changes during co-culture and identify enhanced ADH pathway activity as a factor in in the mutualistic growth observed during co-culture. They also demonstrate up regulation of key enzymes in this pathway (CK1) during co-culture with BVAB species and chemically inhibit polyamine biosynthesis demonstrating its importance for mutualistic growth.

Strengths:

1. Use of multiple BVAB species provides good evidence that this mutualistic growth is not specific to any one bacterial species. This is important information in a disease state associated with multiple bacteria.

2. Metabolically profiling co-cultures over multiple timepoints is used well to establish early and late changes in the metabolic environment.

3. Despite being unable to isolate a CK knock out parasite line the attempt is greatly appreciated especially in an organism that is as difficult to manipulate genetically as T. vaginalis.

Weaknesses:

1. There is a severe lack of experimental description in the methods section that makes it difficult to assess what exactly was done in experiments described in figures 1-4 and 8. For example viability of parasites is often referenced, but there is no indication of how viability was measured. Flow cytometry analysis is not supported with a figure showing the gating strategy.

2. Bacterial growth is only qualitatively assessed even in instances where only one bacterial species is used in co-culture (Figure 8).

3. Status of the Mycoplasma hominis or Mycoplasma girerdii in parasites used in the experiments is not indicated, but an important control for the interpretation of the findings.

4. Figures and figure legends often lack important information such as what error bars represent, significance values etc.

5. ATP production during co-culture should be quantified especially with previous reports on ADH pathway utilization in Mycoplasma hominis / T. vaginalis studies.

Reviewer #3: This is a very interesting and important study that demonstrates that the protist TV and selected BVAB engage in metabolic cooperation (syntrophy) that enables mutual growth under nutrient-limited conditions where neither can grow alone.

Mechanistically, co-cultures with BVAB (but not with Lactobacillus gasseri) are inducing a metabolic shift toward amino acid catabolism, centred on the ADH pathway and polyamine (e.g., putrescine) production, which together provide energy and growth-supporting metabolites.

Functionally, blocking polyamine synthesis disrupts this cooperation, confirming that this metabolic axis is essential for the interaction. The KO of a key gene in TV relevant metabolic pathway was apparently lethal, which did not allow to further investigate the contribution of TV in the syntrophy. A regulated KO system, which is currently not available for TV, might be able in the future to perform more detailed analyses.

The presented data and their conservative interpretation provide important new insights into TV-bacteria interactions and their potential relevance to the pathobiologies associated with TV and BVAB. The work provides strong experimental evidence that cross-kingdom metabolic cooperation could contribute at stabilising both TV and BVAB dysbiosis in vivo. These data strongly support a model in which TV is not acting as an isolated pathogen but represents an active participant in a metabolically interdependent microbial ecosystem.

This is a very well executed study with no major identified weaknesses (but see earlier comment on the KO phenotype and comments below), which is highly novel and significant in that it combines relevant co-cultures phenotyping with complementary metabolomics, gene expression and perturbation data (drug treatment and KO in TV).

I have only a few minor comments, including a few details relating to data presentation and scholarship that should be considered to improve the manuscript in places.

**********

**Part II – Major Issues: Key Experiments Required for Acceptance**

Please use this section to detail the key new experiments or modifications of existing experiments that should be absolutely required to validate study conclusions.

Generally, there should be no more than 3 such required experiments or major modifications for a "Major Revision" recommendation. If more than 3 experiments are necessary to validate the study conclusions, then you are encouraged to recommend "Reject".

Reviewer #1: The limitations of the study should also be more clearly acknowledged. In particular, the manuscript lacks in vitro co-culture systems including human host cells; since the authors used a protozoan strain that does not harbor any Mycoplasma endosymbiont species, the contribution of these bacteria remains unclear; no in vivo experiments were performed; and the study was conducted using only a single protozoan isolate.

Figures

The quality and resolution of the figures and tables should be improved.

Reviewer #2: 1. There is no mention of the Mycoplasma hominis or Mycoplasma girerdii status of the Trichomonas vaginalis parasites used in this paper. The presence of an additional bacterial species, one also observed to be present during vaginal dysbiosis (M. hominis) may alter the interpretation of these results. At a minimum the authors should test for their presence in the parasites used via PCR.

2. For figure 2-4 how is viability assessed? This is not stated in methods, text, or figure legend. If done via Flow with a viability stain, a gating scheme for analysis of viability should be provided with the appropriate controls (i.e. a dead parasite control for whatever method is being used to determine this is).

3. While the authors state that the three BVAB species cannot be distinguished in their spot plating assays, they should at least quantify total CFU (of all colonies regardless of species) to provide some assessment of bacterial growth during co-culture.

4. An assay to assess overall ATP production such as the one referenced in a previous paper (see reference 29 of manuscript) for coculture with BVAB should be incorporated into the paper to support Mass spec data and the increased utilization of the ADH pathway in co-culture.

5. For Figure 8, since only G. vaginalis is being used, the authors should quantify G. vaginalis levels.

Reviewer #3: There are published evidence that TV can phagocytose some Lactobacillus species (e.g. Rendón-Maldonado et al. (1998). Experimental parasitology 89.2: 241-250). Are there any relevant data for the 3 tested BABV species? It would be of great interest to perform some TEM on these co-cultures to check for this if the logistic to generate such data is not to difficult/onerous.

**********

**Part III – Minor Issues: Editorial and Data Presentation Modifications**

Please use this section for editorial suggestions as well as relatively minor modifications of existing data that would enhance clarity.

Reviewer #1: Several points should be revised:

Introduction

Lines 80–81: This statement requires an appropriate reference.

Results

Lines 137–138: This statement is too vague. The composition of culture media for many microorganisms is often similar regardless of their specific nutritional requirements. In general, media that support microbial growth are highly enriched in nutrients, often in a non-specific manner. This sentence should therefore be rewritten or removed.

Lines 309–310: I could not identify Figure 7C.

Discussion

Line 379: Please correct the word “aammonia”.

Materials and Methods

Line 474: The authors should specify that the reference strain G3 is not infected by any Mycoplasma species. Which dsRNA viruses are present in this strain?

Lines 496–497: Why were bacterial counts not assessed as colony-forming units (CFU)? How reliable is the method used to estimate bacterial load?

Lines 502–503: Was viability monitored, particularly for the protozoa? At high concentrations, T. vaginalis may undergo substantial death after 32 hours of incubation.

Lines 546–547: Why was gene expression analysis performed only at 8 hours of incubation?

Reviewer #2: 1. Line 29-30 Inhibition of polyamine biosynthesis the cooperative growth… There appears to be a word missing between biosynthesis and the. Perhaps should read: “Inhibition of polyamine biosynthesis impaired the cooperative growth …”

2. Line 91 “Despite increasing awareness…” does not make sense here. Should just read: “Historical precedent ….”

3. Line 803 (and other locations) spot platting should be spot plating?

4. Line 159 Please note Figure 1 in parentheses with the p-values.

5. What pH is the DMEM at in these experiments? Please state this in the methods. T. vaginalis is typically grown between pH 5.8-6.2 in TYM media. If the pH for the DMEM experiments is not adjusted this is an interesting observation to note for the co-culture experiments.

6. For experiments involving flow cytometry (Fig. 1…) a supplemental figure showing the gating strategy must be provided. The gating strategy is likely very simple, but it is standard practice to include an example of it.

7. It is not always clear how many bacteria are being added in co-culture experiments. In materials and methods (Line 491) it states “Pre-stained T. vaginalis and bacteria were mixed at a 1:10 ratio” this is slightly unclear regarding the figure legends. For example, Fig 1 legend (Line 802-803) states: “the three BV-associated bacteria (BVAB) P. bivia, A. vaginae and G. vaginalis (square), with an initial inoculum of 2 x 104 protozoal cells per ml…”. Are 2 x105 total bacteria being added to the coculture or 2 x105 bacteria for each BVAB species. Please clarify in the methods or figure legend for figures showing co-culture experiments. Also is there any reason this cannot be stated in a more conventional manner such as CFU?

8. Materials and methods state (Line 490) “only Trichomonas cells were fluorescently stained for flow cytometry analysis”. Please state how they were stained (what dye/concentration). As stated previously a gating scheme for the flow cytometry analysis would be very helpful.

9. Line 812 states “, the number of highly viable protozoal cells”. What does highly viable mean here? It is not stated in the methods how viability is determined or what highly means in this context compared to something like slightly viable.

10. Figure legends should state what the error bars and bars represent and what statistical tests were used.

11. Line 499-500 states: “one-way ANOVA was employed to compare the median T. vaginalis cell numbers between mixed and single cultures”. Please clarify why the median was used and what the error bars represent.

12. Figure 3: Please add either numerical values or symbols indicating p-values for comparisons to no bacteria control on graph.

13. Figure 3 tiff has odd blurring and color scheme obscures some errors bars. Recommend removing fill on bar graphs or changing color of error bars. Also please indicate what error bars represent in figure legend.

14. Figure 4 please indicate what error bars represent in figure legend.

15. Line 173-174 states “As before, despite highly viable, T. vaginalis did not grow axenically in this condition…”. I think a word is missing again should it read “As before, despite being highly viable ….”. Also, no viability data is shown here and as in Figure 2 if this is done via a viability stain using flow cytometry a gating scheme should be included as supplemental with appropriate controls.

16. For Figure 6 adding a label indicating putrescine and ornithine to the box plots would be helpful.

17. Figure 7 and the text (Line 310) reference a 7C. There is no C in the figure. Instead, all the data is represented in 7B. Please update graph or text accordingly.

18. Please state what error bars represent in Figure 8 and add p-values to graph.

Reviewer #3: Additional relevant literature needs to be consider to enrich the manuscript relating to its relevance and importance. The introduction and discussion needs to be enriched, made more precise in places, by incorporating the below issues:

- The introduction should specifically refer to the 3 selected bacteria for co-culture experiments with TV as core/major BV-associated bacteria. Indeed, a key review on the topic is cited in the introduction (ref 2: Bradshaw, et al. (2025). Bacterial vaginosis. Nature Reviews Disease Primers 11.1:43). Make sure to cite it correctly, see repetition for year/volume/issue. Similarly, in lines 42-43, the sentence should specify that a subset of the core/major bacteria characteristic of BV where investigated

- Relevant paper on Trichomonas-bacteria co-cultures experiments illustrating their potential role on Trichomonas growth and pathobiology, covering TV and related species:

Jirovec, O., & Petrů, M. (1968). Trichomonas vaginalis and trichomoniasis. Advances in parasitology, 6: 117-188. This is a very interesting historical review that discusses a number of relevant TV-bacteria interactions. Hence the interesting historical section on Entamoeba should be enriched by highlighting this more directly relevant review for TV.

Margarita, et al. (2022). Two different species of Mycoplasma endosymbionts can influence Trichomonas vaginalis pathophysiology. MBio 13.3: e00918-22.

Hart et al. (2025). An evolutionarily conserved laterally acquired toolkit enables microbiota targeting by Trichomonas Molecular Biology and Evolution 42.11: msaf276.

The supplementary Tables 1 and 2 and Supplementary Data 1 and 2 would need to include additional information to make it easier/straightforward to read the different sub-tables without having to refer to other sections of the manuscript. It will be essential to provide all the relevant details of each sections/sub-tables in these 4 files.

Abstract: the sentence lines 29-30 seems to be incomplete.

“Inhibition of polyamine biosynthesis the cooperative growth of both microorganisms, further supporting the importance of this metabolic axis.”

Acronyms: ensure consistent use across the manuscript: mostly 2HB, but 2-HB in line 460.

**********

PLOS authors have the option to publish the peer review history of their article (what does this mean?). If published, this will include your full peer review and any attached files.

If you choose “no”, your identity will remain anonymous but your review may still be made public.

**Do you want your identity to be public for this peer review?**  For information about this choice, including consent withdrawal, please see our Privacy Policy.

Reviewer #1: **Yes:**  Pier Luigi Fiori

Reviewer #2: No

Reviewer #3: No

**Figure resubmission:**

**Reproducibility:**

-->?>

# Author comment: Author response to Decision Letter 1

**DOI:** 10.1371/journal.ppat.1014620.r002

2 Jul 2026

Attachment Submitted filename: Response to editor and reviewers.docx

# Peer review: Decision Letter 1

**DOI:** 10.1371/journal.ppat.1014620.r003

**Authors:** Dominique Soldati-Favre, William Navarre

31 Aug 2026

Dear Dr. Simoes-Barbosa,

We are pleased to inform that your manuscript, "Metabolic cooperation supports mutual growth between the protozoan parasite Trichomonas vaginalis and dysbiotic vaginal bacteria", has been editorially accepted for publication at PLOS Pathogens.

Before your manuscript can be formally accepted and sent to production, you will need to complete our formatting changes, which you will receive by email within a week. Please note that your manuscript will not be scheduled for publication until you have made the required changes.

IMPORTANT NOTES

(1) Copyediting and Proofreading: The corresponding author will receive a typeset proof for review, to ensure errors have not been introduced during production. Please review the PDF proof of your manuscript carefully, as this is the last chance to correct any errors. Please note that major changes, or those which affect the scientific understanding of the work, will likely cause delays to the publication date of your manuscript.

(2) Appropriate Figure Files: Please remove all name and figure # text from your figure files. Please also take this time to check that your figures are of high resolution, which will improve the readbility of your figures and help expedite your manuscript's publication. Please note that figures must have been originally created at 300dpi or higher. Do not manually increase the resolution of your files. For instructions on how to properly obtain high quality images, please review our Figure Guidelines, with examples at: http://journals.plos.org/plospathogens/s/figures.

We encourage you to use PACE (http://pace.apexcovantage.com/), a figure-checking tool which helps authors ensure all figures meet PLOS requirements so that figures can publish in the highest quality. PACE is a digital diagnostic and conversion tool for figure files. It will provide information about any failed check(s) and, if able, will automatically convert the figure into an acceptable file that passes quality checks.

To use PACE, you must first register as a user. Then, login and navigate to the UPLOAD tab, where you will find detailed instructions on how to use the tool.

(3) Striking Image: Please upload a striking still image to accompany your article if one is available (you can include a new image or an existing one from within your manuscript). Should your paper be accepted, this image will be considered for our monthly issue image and may also appear on our website to feature your article. Please upload this as a separate file, selecting "striking image" as the file type upon upload. Please also include a separate "Other" file with a caption, including credits and any potential copyright information. Please do not include the caption in the main article file. If your image is from someone other than yourself, please ensure that the artist has read and agreed to the terms and conditions of the Creative Commons Attribution License at http://journals.plos.org/plospathogens/s/content-license. Please note that PLOS cannot publish copyrighted images.

(4) Press Release or Related Media: If you or your institution will be preparing press materials for this manuscript, please inform our press team in advance at plospathogens@plos.org as soon as possible. We ask that you contact us within one week to plan ahead of our fast Production schedule. If you need to know your paper's publication date for related media purposes, you must coordinate with our press team, and your manuscript will remain under a strict press embargo until the publication date and time.

(5) Update your Profile Information: Now that your manuscript has been provisionally accepted, please log into Editorial Manager and update your profile, if needed. Go to http://www.editorialmanager.com/ppathogens, log in, and click on the "Update My Information" link at the top of the page. Please update your user information to ensure an efficient production and billing process.

(6) LaTeX users only: Our staff will ask you to upload a TEX file in addition to the PDF before the paper can be sent to typesetting, so please carefully review our Latex Guidelines http://journals.plos.org/plospathogens/s/latex in the meantime.

Thank you again for supporting Open Access publishing; we are looking forward to publishing your work in PLOS Pathogens.

Best regards,

William Navarre

Academic Editor

PLOS Pathogens

Dominique Soldati-Favre

Editor-in-Chief

PLOS Pathogens

Dominique Soldati-Favre

Editor-in-Chief

PLOS Pathogens

***********************************************************

All reviewers found that the manuscript was greatly improved. Thank you for responding to their concerns so thoroughly.

Reviewer's Responses to Questions

**Part I - Summary**

Please use this section to discuss strengths/weaknesses of study, novelty/significance, general execution and scholarship.<br/><br/>

Reviewer #1: The manuscript has been considerably improved with the addition of substantial clarifications. I believe that, in its current state, it does not present any particular weaknesses

Reviewer #2: The authors have addressed the previous major and minor concerns raised in the review.

Reviewer #3: The authors have satisfactorily addressed all the key issues identified in the earlier version of the manuscript, clearly improving the overall execution and scholarship of the study. In particular, they have clarified the mycoplasma status of the parasite and generated relevant additional metabolomics data that complement and strengthen the original findings. Notably, the conclusions are also now presented more clearly and in an appropriate, more conservative manner (e.g. recognising several limitations: absence of vaginal epithelial cells and lack of in vivo data), better reflecting the scope and limitations of the presented data. Overall, these revisions have improved the robustness of the study and the support for its main conclusions.

--------------------

**Part II – Major Issues: Key Experiments Required for Acceptance**

Please use this section to detail the key new experiments or modifications of existing experiments that should be absolutely required to validate study conclusions.<br/><br/>Generally, there should be no more than 3 such required experiments or major modifications for a "Major Revision" recommendation. If more than 3 experiments are necessary to validate the study conclusions, then you are encouraged to recommend "Reject".<br/><br/>

Reviewer #1: It is not necessary to add further experiments

Reviewer #2: The authors have included data showing Mycoplasma status and included a discussion of its potential role in the studies findings.

The authors have added an analysis of DFMO-treated culture supernatants that strengthen findings regarding the ADH/polyamine metabolism.

The authors have addressed concerns regarding the lack of methodological detail sufficiently.

While, the authors have not included CFU quantification for either mixed BVAB or individual species, they have re-emphasized the qualitative nature of the assessment.

Reviewer #3: All key additions/corrections relating to additional experiments were covered in the revised manuscript.

--------------------

**Part III – Minor Issues: Editorial and Data Presentation Modifications**

Please use this section for editorial suggestions as well as relatively minor modifications of existing data that would enhance clarity.<br/><br/>

Reviewer #1: The minor points have also been clarified

Reviewer #2: The authors have addressed minor issues indicated.

Reviewer #3: All key additions/corrections relating to editorial and data presentations were covered in the revised manuscript. One detail, I would clarify in Supplementary Data 1, Figures A2, A5, and A8 the top five components considered to generate that figure. Are they the top 5 listed in the Figure A1/A4/A7, or else? Same with the related figures in part B.

--------------------

PLOS authors have the option to publish the peer review history of their article (what does this mean?). If published, this will include your full peer review and any attached files.

If you choose “no”, your identity will remain anonymous but your review may still be made public.

**Do you want your identity to be public for this peer review?**  For information about this choice, including consent withdrawal, please see our Privacy Policy.

Reviewer #1: Yes: Pier Luigi Fiori

Reviewer #2: No

Reviewer #3: No

# Editor report: Acceptance letter

**DOI:** 10.1371/journal.ppat.1014620.r004

**Authors:** Dominique Soldati-Favre, William Navarre

Dear Dr. Simoes-Barbosa,

We are delighted to inform you that your manuscript, "Metabolic cooperation supports mutual growth between the protozoan parasite Trichomonas vaginalis and dysbiotic vaginal bacteria," has been formally accepted for publication in PLOS Pathogens.

We have now passed your article onto the PLOS Production Department who will complete the rest of the pre-publication process. All authors will receive a confirmation email upon publication.

Thank you again for supporting open-access publishing; we are looking forward to publishing your work in PLOS Pathogens.

Best regards,

Dominique Soldati-Favre

Editor-in-Chief

PLOS Pathogens
